# PrimerSuite: A High-Throughput Web-Based Primer Design Program for Multiplex Bisulfite PCR

**DOI:** 10.1038/srep41328

**Published:** 2017-01-24

**Authors:** Jennifer Lu, Andrew Johnston, Philippe Berichon, Ke-lin Ru, Darren Korbie, Matt Trau

**Affiliations:** 1 Centre for Personalized NanoMedicine, The University of Queensland, St Lucia 4072, QLD, Australia; 2 Australian Institute for Bioengineering and Nanotechnology, The University of Queensland, St Lucia 4072, QLD, Australia; 3 School of Chemistry and Molecular Biosciences, The University of Queensland, St Lucia 4072, QLD, Australia

## Abstract

The analysis of DNA methylation at CpG dinucleotides has become a major research focus due to its regulatory role in numerous biological processes, but the requisite need for assays which amplify bisulfite-converted DNA represents a major bottleneck due to the unique design constraints imposed on bisulfite-PCR primers. Moreover, a review of the literature indicated no available software solutions which accommodated both high-throughput primer design, support for multiplex amplification assays, and primer-dimer prediction. In response, the tri-modular software package *PrimerSuite* was developed to support bisulfite multiplex PCR applications. This software was constructed to (i) design bisulfite primers against multiple regions simultaneously (***PrimerSuite***), (ii) screen for primer-primer dimerizing artefacts (***PrimerDimer***), and (iii) support multiplex PCR assays (***PrimerPlex***). Moreover, a major focus in the development of this software package was the emphasis on extensive empirical validation, and over 1300 unique primer pairs have been successfully designed and screened, with over 94% of them producing amplicons of the expected size, and an average mapping efficiency of 93% when screened using bisulfite multiplex resequencing. The potential use of the software in other bisulfite-based applications such as methylation-specific PCR is under consideration for future updates. This resource is freely available for use at *PrimerSuite* website (www.primer-suite.com).

The methylation of cytosine at the carbon-5 position (5-methylcytosine) is an epigenetic mark associated with the regulation of numerous cellular processes in the mammalian genome such as embryonic development, genomic imprinting, X chromosome inactivation, and preservation stability^1^,^2^, and aberrant patterns of DNA methylation have been implicated in various pathologies such as cancer. Advancements in genome-wide methylation analysis technologies (e.g. Illumina Infinium *HumanMethylation450* Beadchip arrays, whole-genome bisulfite sequencing) have driven research in this area over the past decade, and a key feature of many DNA methylation assays is the use of the bisulfite treatment process. While methylated cytosines are not affected by this chemical treatment, unmethylated cytosines are converted to deoxyuracils and participate in subsequent primer annealing and amplification as thymines, and by comparing the ratio of thymines to cytosines for a locus the overall methylation state of a gene or a genome can be determined^3^,^4^.

While the bisulfite method remains the gold standard for quantitative profiling of the methylation status of target DNA at a single base pair (bp) resolution, applications which utilize PCR primers to amplify the bisulfite-converted DNA templates have additional design constraints unique to this application. For example, the long stretches of thymines and adenines introduced into the template and the potential to have CpG dinucleotides present in the primer can both impact the fidelity of PCR amplification, and may lead to amplification bias or the formation of unwanted dimers during the reactions if not properly controlled for ref. 5. While these considerations can be carefully deliberated when optimizing a single primer pair, they are less manageable when working with a large number of candidate regions. Furthermore, while a protocol for multiplex bisulfite PCR has been published which allows for rapid screening of multiple regions simultaneously^6^, this validation resequencing method is still dependent on the ability to rapidly design bisulfite primers against dozens of DNA sequences. Since many genome-wide epigenetic discovery projects are left with hundreds of differentially methylated regions of statistical significance, effective bisulfite primer design therefore represents a substantial bottleneck in the validation process^7^. Moreover, while a number of automated programs for bisulfite primer design have been created, a review of their features demonstrated that many of them were of limited use; for example, many restricted users to input a single DNA sequence, or failed to consider the likelihood of PCR dimers and off-target effects during amplification. Critically, a review of current literature indicated none of the publically available tools were designed to support multiplex PCR methods (i.e., the amplification of multiple amplicons in a single PCR reaction)^8^,^9^,^10^,^11^.

In response, we present here a custom high-throughput web-based program to support bisulfite PCR and multiplex PCR assays called *PrimerSuite*, which is composed of three modules: PrimerSuite (PS) – for generation of bisulfite and genomic primers from multiple sequences; PrimerDimer (PD) – for prediction of primer dimer formation between single primer pairs or multiple oligonucleotides in a multiplex pool; and PrimerPlex (PP) – for grouping of primers into different pools for multiplex PCR amplification. The design considerations which went into the PrimerSuite software were based on several key criteria for bisulfite PCR that were discovered and optimized during two recently published genome-wide methylated biomarker discovery projects, and which required high-throughput validation and primer design for a large numbers of regions^6^,^7^. Moreover, this software solution represents the first study where the functionalities of a program has undergone extensive iterative empirical testing, with over 1300 primer pairs designed and screened in the lab to date to ensure the efficiency of the software. Although this suite of software was originally scripted to generate primers for multiplex bisulfite PCR applications, its broad features renders it suitable for a number of other applications in future updates such as methylation specific PCR, recombinase-polymerase-amplification assay and ligase chain reactions^12^,^13^,^14^. The *PrimerSuite* software package is freely available and its three modules can be accessed online via www.primer-suite.com (PrimerSuite), www.primer-dimer.com (PrimerDimer) and www.primer-plex.com (PrimerPlex).

## Results and Discussions

### Assessment of publically available bisulfite primer design tools against key criteria

Many methods for DNA methylation analysis require bisulfite-converted DNA as the starting material and use custom primers against this template in an amplification process. While the bisulfite method allows methylated cytosines to be distinguished and quantified, primer design targeting bisulfite-converted templates can be challenging due to the restrictions imposed on both the template and the primers (e.g. the introduction of polyT’s or polyA’s stretches in the template after bisulfite conversion). Our previous work on the discovery and validation of genome-wide methylated biomarkers^6^ identified several key aspects critical for bisulfite PCR primer design, and based on these observations a set of seven criteria were outlined which a bioinformatics solution for high-throughput primer design should possess. These were:

1. It should have the ability to input multiple DNA FASTA sequences for analysis, as genome-wide projects frequently have hundreds of differentially methylated regions of significance which need to be assayed.
2. It should be able to differentiate the position of bases which have undergone bisulfite conversion, and position unconverted bases preferentially at the 3′ end of the primer. This was considered a key parameter since the 3′ end of PCR primers define the amplification fidelity of the overall reaction, and by selecting a series of unconverted bases at the 3′ end an overall increase in the fidelity of template amplification should be observed.
3. The number of CpG dinucleotides in both the primer and the amplicon should be a selectable parameter, allowing users to either include or exclude them from the primer design process. Moreover, for situations where CpG dinucleotides have been included in the PCR primers, users should be able to control whether the CpG(s) should be preferentially positioned towards the 5′ or 3′ end of the oligonucleotide.
4. The long stretches of thymines and adenines introduced into the template as a result of the bisulfite-conversion process should be considered during primer design and reported on, as primers designed against these regions could lack specificity.
5. The program should evaluate and report oligonucleotides designed against both the Watson and Crick strands of DNA (often referred to as the C-to-T verses the G-to-A strands). This is because the bisulfite conversion process renders the two strands of DNA non-complementary, and in many cases primer design against one strand will generate suitable primers when the opposite strand will not.
6. The program should offer an assessment of the likelihood of primer-dimer formation based on free energy (ΔG) calculations, and allow users to apply a cut-off value for optimal ΔG.
7. The program should support multiplex PCR applications, and sort primer pairs into separate pools based on their relative amplification efficiency and predicted likelihood of forming dimers when combined together.

**Figure 1:**
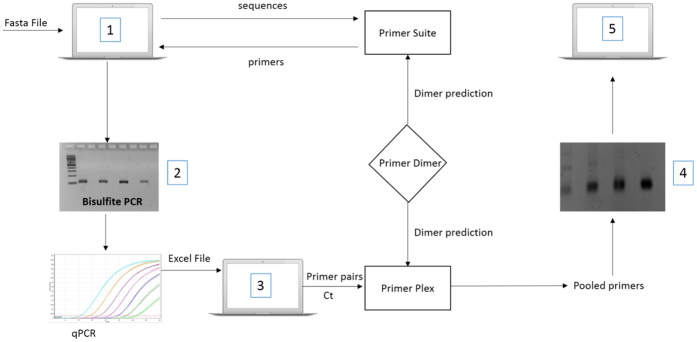
Summary of the PrimerSuite software pipeline. (**1**) Using sequences pasted into the webpage or uploaded as a FASTA file primers are designed according to the user-adjustable parameters; PrimerDimer is embedded to predict possible dimerization between primers. (**2**) Selected primer pairs are validated using bisulfite-PCR and the efficiency of primer pairs are analysed using qPCR. (**3**) Non-overlapping primers pairs are sorted into different pools according to Ct values from qPCR, which are used as a proxy-metric for amplification efficiency. (**4**) Amplicons screened in this way are pooled using PrimerPlex and after amplification are sequenced using next-generation sequencing platforms.

Six publically-available bisulfite primer design programs^8^,^9^,^10^,^11^ including www.epidesigner.com and www.zymoresearch.com were evaluated against the seven key criteria list above, the results of which are listed in Table 1. To evaluate each program, the DNA sequence from the coordinates listed in Table 2 were submitted through each program and each criteria was assessed accordingly. Based on the observations made during this analysis, none of the programs tested fulfilled all seven of the key criteria identified. Although the same parameters were used whenever possible (i.e. Tm of 54 °C, amplicon size between 120–140 bp, zero CpG’s allowed in the primers), different primer pairs were obtained from each program, and some programs failed to report any valid primers for certain CpG-rich regions, which potentially posed a problem for many analyses given that the majority of methylation studies are focused on CpG-rich promoter regions. Additionally, none of the programs tested could process primers into pools for multiplex assays, a critical feature needed to support custom bisulfite multiplex assays^6^, although the program ‘MPprimer’ supported multiplexing of genomic primers^15^. In comparison, when the same analysis was performed with *PrimerSuite*, the program successfully returned primers for all DNA sequences entered, including CpG-rich regions which other programs failed at generating oligonucleotides to. Furthermore, in this review it was also noted that empirical wet lab validation data supporting the robustness of the respective programs was very limited, with the majority of software solutions testing five or less primer pairs to demonstrate their efficacy and utility (Table 1). Therefore, a new pipeline for high-throughput primer design for multiplex bisulfite PCR assays was devised in this study with the major steps highlighted in Fig. 1.

Additionally, the high-throughput primer design tool MSP-HTPrimer^16^ was also analysed using the criteria described above. In contrast to the other programs analysed in Table 1, experimental validation was performed on 66 bisulfite-specific PCR primer pairs of which 63 primer pairs were successfully validated without further optimisation. Although this web-based program was described as a highly efficient program for designing primers for various bisulfite-based assays such as bisulfite specific PCR, methylation specific PCR and pyrosequencing, it does not have the multiplexing capabilities required for bisulfite multiplex PCR resequencing and was not considered further in this study. However, the capabilities of the program to design primers for a number of bisulfite conversion-based PCR assays enables it to be a great tool for other methylation studies.

### Primer Design Algorithm Development

For this study primers were generated against diagnostic and prognostic regions that were found to be hypermethylated in triple negative breast cancer (TNBC)^7^ by uploading a multi-FASTA file and running *PrimerSuite* with the following parameters: 0 CpG’s allowed in the primers, an amplicon size between 120–140 base pairs, primer melting temperature of 54 °C, sodium concentration of 50 mM, a minimum of at least 4 CpGs in the amplicon, and at least 3 consecutive unconverted bases starting from the 3′ end of each primer (referred to as the primer score); these are set as default parameters on the PrimerSuite website. The PrimerSuite module generates all possible primers by ‘walking’ along the region of interest one base at a time, and calculates the melting temperature of the oligonucleotides based on the salt-adjusted melting temperature^17^,^18^,^19^ with each primer pair filtered according to the parameters as specified by the user (e.g. CpG’s in primer, amplicon size etc). Compared to other programs reviewed in this study^8^,^9^,^10^,^11^,^20^,^21^ such as www.epidesigner.com and www.zymoresearch.com, PrimerSuite offers the user more flexibility in their choice of primers as it reports every primer pair that fulfils a set of user-specified criteria. As the PS algorithm looks for primers by ‘walking’ along the sequence one base at a time, it is especially efficient in reporting primer pairs within CpG-rich regions, whereas these regions were found to be especially problematic when processed by other programs (Table 1)^8^,^9^. Furthermore, when multiple DNA regions of 1–3 kilobases are entered the average processing time per sequence was estimated to be 2–3 seconds, as compared to other programs which can only process a single sequence at a time.

### Validation of PrimerSuite-generated primers using bisulfite PCR

To confirm the effectiveness of the PrimerSuite program in generating bisulfite primers, 100 PrimerSuite-designed primer pairs were initially selected for wet-lab screening and validation. Each primer pair was assayed in a single-plex PCR reaction alongside no-template controls (NTC), and results from this experiment demonstrated that each primer pair produced amplicons of the expected size (i.e. 120–140 bp) when visualised on an agarose gel (Supplementary Figure S1). However, the appearance of additional primer dimers in six of the samples (indicated by **#** in Supplementary Figure S1) demonstrated the need for a dimer prediction module, as dimerization between oligonucleotides may decrease the efficiencies of the assays during amplification and reduce sequencing depth in the final library. Therefore a secondary program called PrimerDimer was created to predict the formation of dimer artefacts during PCR using previously published free energy calculations^18^,^22^,^23^,^24^,^25^,^26^,^27^,^28^.

### Prediction of oligonucleotide dimer formation using PrimerDimer

**Figure 2:**
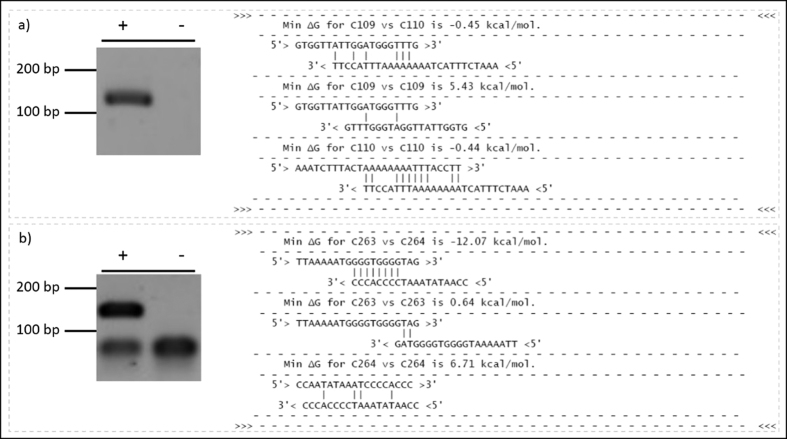
Representative Dimer report of dimerization structures of two primer pairs. To determine if the new PD algorithm can predict the formation of secondary dimer products, gel images of each amplified product (**left**) was compared with the dimerization structures of the primer pairs which were used to amplify the corresponding product (**right**). (**a**) The stability of each combination is reported as the minimum ΔG (kcal/mol) value. A representative primer pair which produced no visible dimer artefacts (**left**) and had no stable 3′ end structures, as determined by the minimum ΔG of each structure (**right**). (**b**) Alternatively, a representative primer pair which produced a strong dimer artefact on the electrophoresis gel (**left**) has a strong structure formed between the 3′ ends of the forward (C263) and reverse (C264) (**right**) primer as evident with the stable minimum ΔG value.

Initial review of the most prominent dimer-forming primer pairs suggested that dimer formation between primers correlated to a high level of complementarity at the 3′ end of the relevant oligonucleotides (Representative data as shown in Fig. 2). Subsequent sequencing analysis of the dimer artefacts (data not shown) validated the hypothesis that dimerization frequently seemed to result from the binding of primers at the 3′ end to its reciprocal mate (heterodimer) and/or to itself (homodimer), along with a concomitant high free-energy at the 3′ end (i.e. primer-primer annealing which is thermodynamically stable enough for extension and subsequent amplification to occur). To control for these events the PrimerDimer module was implemented to identify primer pairs which have the potential to dimerize during amplification, and predicts the likelihood of dimer artefacts by using previously-published free-energy calculations^18^,^22^,^23^,^24^,^25^,^26^,^27^,^28^. To assess the predictive efficiency of the PrimerDimer module, primers from the initial validation of PS were parsed through the program, and the minimum free-energy score of all possible dimer structures was calculated and compared to the intensity of the dimer artefacts on the gel as highlighted in Supplementary Figure S2. A summary of the worst dimer formation of each primer pair from this initial panel can be viewed in **Additional File 1**. Based on these observations, a second panel of PS-generated and PD-screened primers with high free-energy scores (i.e., those with a low probably of forming dimer artefacts) was further screened in the lab (Supplementary Figure S3). Compared to the initial screen, only one primer pair produced prominent dimers (**C443 and C444**) which suggested that the free-energy scoring system implemented in the PD algorithm correctly identified likely dimerization events between primer pairs. Based on this observation, the PD algorithm was then incorporated into the PS primer design pipeline. This improvement provides a free-energy prediction in the final PS-output report which can be used as a proxy for the likelihood of dimerization between primers, which should increase the overall fidelity of assay design.

### Prediction of PCR fidelity by assessing uniqueness of primers in the genome

**Figure 3:**
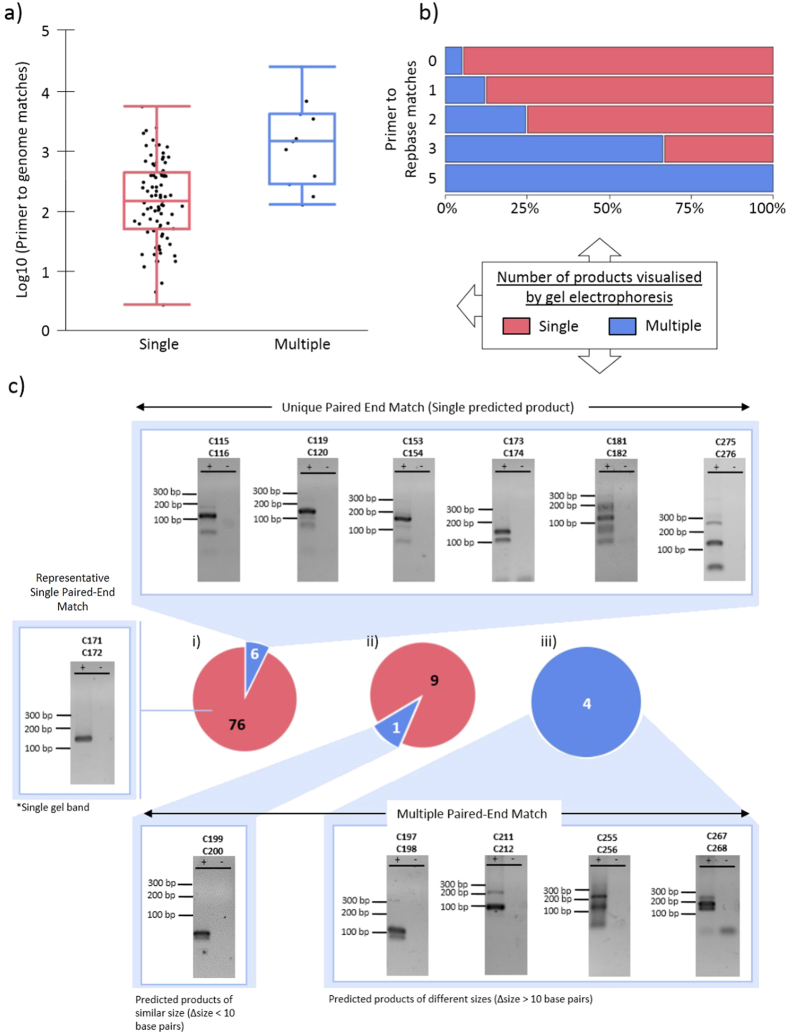
Uniqueness of primers in the genome as a predictor of PCR fidelity. To determine if uniqueness of primers in the genome can be used to predict the fidelity of PCR, primers were mapped to two different reference genomes, both of which were bisulfite converted prior to mapping. (**a**) Primer-to-human-genome (hg19) mapping. Distribution of the number of primer-to-genome matches when mapping with Bowtie1 in single-end mode against hg19, as a function of PCR product as visualized by DNA gel electrophoresis. The box-and-whisker plot shows a correlation between the numbers of primer-to-genome matches to number of gel bands. (**b**) Primer-to-Repbase mapping. The stacked bar chart shows an increase in the number of exact primer-to-reference-matches when mapping with Bowtie1 in single-end mode. Overall the number exact primer-to-reference-matches increased with the number of PCR products visualized on a DNA gel (i.e. single verses multiple products). (**c**) Visualisation of amplicons which produced different sized gel bands. Of the 82 primer pairs which mapped uniquely to the hg19 genome (**i**), 76 produced a single PCR product. Of the 10 primer pairs which mapped to multiple loci (**ii**), only one produced a single PCR product as visualized by DNA gel electrophoresis; whereas all primer pairs which mapped multiple times with a difference in amplicon size between the smallest and largest products of at least 10 base pairs can be seen on gel as multiple bands. Although the target amplicon of approximately 120 base pairs appeared to be present in all the gel images (**iii**), the presence of other PCR products in the gel was hypothesised to be the amplification of repetitive sequences as indicated by alignment of primers against the Repbase (*homo sapian*) repetitive regions library.

Some primer design programs have implemented a feature to screen for ‘uniqueness’ of primers in a reference genome as a method to predict the extent to which a primer pair will accurately amplify the region of interest^20^,^21^. If the number of primer-to-genome-matches was sufficient to predict PCR fidelity, then the primer pairs with the greatest amount of secondary non-dimer product(s) (as shown in Supplementary Figure S1 (*)) should correlate with the highest number of primer-to-genome matches. To determine if this hypothesis was valid and could be used as a predictor of a primer pair’s ability to correctly amplify target amplicons of interest, the 100 primer pairs from the first PS validation (Supplementary Figure S1) were mapped to both the human genome (hg19) and a library of repetitive sequences obtained from Repbase, whereupon both reference genomes were bisulfite converted prior to mapping. Mapping of primer pairs was performed in both paired-end and single-end modes where all valid alignments were reported, after which the total number of exact occurrences of that primer sequence in the reference genome were tallied; the first 18 nucleotides and 10 nucleotides (from the 3′ end) were also mapped and tallied. It was predicted that as the length of the primers decreased, the number of exact occurrences of the oligonucleotide sequence in the genome would increase and result in a decrease in primer uniqueness. It was also hypothesised that paired-end mapping would have more predictive power in determining the fidelity of PCR amplification since both the forward and reverse primers need to be present in order for the reaction to take place successfully. Therefore, a non-parametric Wilcoxon rank test^29^ was employed to examine the correlation between the number of primer-to-genome matches and the appearance of extra gel bands, with the mean (μ) number of maps summarised in Table 3. From this analysis it was observed there was a statistical correlation between the number of primer-to-human-genome matches and the appearance of additional DNA products as visualized by gel electrophoresis, with an overall p value of <0.0022. However, although the increase in total primer-to-human-genome matches between the two datasets met the threshold for significance, a high number of primer-to-human-genome matches was not sufficient to identify which primers would result in off target amplification. As shown in Fig. 3a, a large degree of overlap occurred between the two datasets, and a significant number of “uniquely-mapping” primer pairs were observed to produce multiple products when visualized on a gel (Fig. 3c, **pie chart** (**i**)). Notably, all the primer pairs which mapped multiple times with a size difference of at least 10 base pairs between the smallest and largest predicted products were visualized on a gel as producing multiple DNA products (Fig. 3c, **pie chart** (**iii**)), although in at least one case a multi-mapping primer pair produced a single visible PCR product on a DNA gel, suggesting that visualization of PCR product on a DNA gel is insufficient to verify PCR specificity. Moreover, analysis of the results from the total primer-to-human-genome matches for the shortened primers (i.e., 18 and 10 nucleotides of the total primer length from the 3′ end), demonstrated similar a trend (data not shown).

When the same analysis was performed against a list of repetitive DNA sequences found in the human genome a similar trend was observed, where the overall number of exact primer-to-repeat-matches increased with the number of PCR products visualized on a DNA gel (i.e. single verses multiple products, Fig. 3b). The similar results between mapping primers to the human genome (Fig. 3a) and the repeat library (Fig. 3b) suggested that a repeat library could be sufficient to help identify sub-optimal sequences (i.e. Primers which could prime against repetitive regions such as LINE and Alu elements) while also being more computationally efficient than screening against the entire human genome. Therefore, to help control for primers which may target repetitive sequences, a library of repetitive elements derived from Repbase was incorporated into the PS module as an optional filtering criteria, and was implemented to screen the first 15 bases (from the 3′ end) of the primer to identify sequences which may result in off-target product. If the screening option is selected, then PS will screen the first 15 bases against either the genomic or methylated Repbase templates depending on whether the user requires genomic or bisulfite primers.

### Optimisation of PrimerPlex to combine primers for multiplex bisulfite PCR

**Figure 4:**
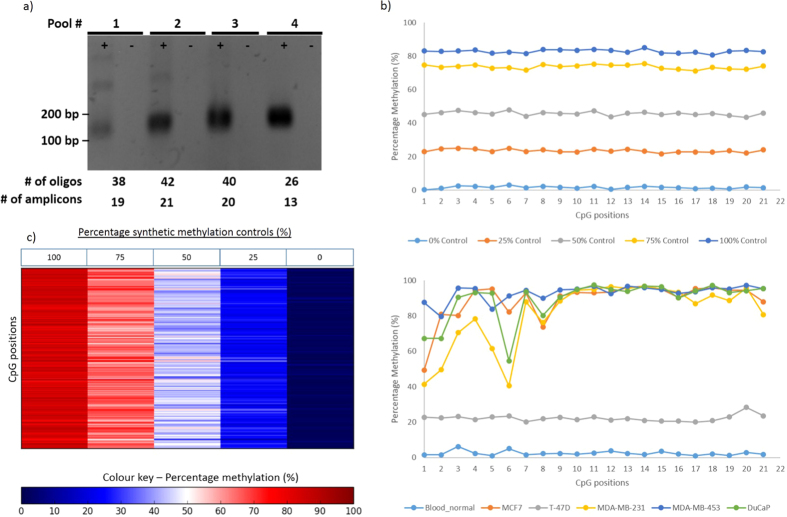
Representative PrimerPlex multiplex PCR libraries. After validation via single-plex bisulfite PCR, validated primer pairs were further analyzed with qPCR to obtain an efficiency score (the Ct value from qPCR) which is used by the PrimerPlex program to combine primers into optimal pools. (**a**) Amplification of the PrimerPlex multiplex assays demonstrated PrimerPlex could successfully pool primers for multiplex amplification with no observable dimer artefact. (**b**) Heat map of the methylation profile of 602 CpG sites of the five methylated controls (100%, 75%, 50%, 25% and 0% methylation controls) across the 178 amplicons assayed. The level of methylation is represented by a colour scale – blue for low level methylation, white for medium, and red for high levels of methylation. Overall, each CpG within the assay had a methylation level which correlated to the expected amount, based on the controls used. (**c**) Representative data for one amplicon with 21 CpG sites. No observable bias within the assay was observed, as demonstrated by the consistent level of methylation across the entire amplicon (**d**) The same amplicon in (**c**) was also assayed against DNA derived from four breast cancer cell lines (MCF7, T-47D, MDA-MB-231, MDA-MB-453), a prostate cancer (DuCaP) cell line, whole blood pool from healthy donors.

We have previously described a method for multiplex bisulfite PCR analysis^6^ and based on the utility of this method an additional design feature for multiplex PCR assays called PrimerPlex was implemented. This module requires users to first screen their well-performing primers using quantitative real-time PCR (qPCR) at a single concentration of template, and use the cycle threshold (Ct) value as a proxy metric for primer efficiency. Next, users upload a file of primer pair sequences and their associated Ct values to the PrimerPlex interface, after which the program then sorts and groups pairs into multiplex pools based on a combination of dimer prediction and similar Ct scores, with an additional sorting feature to ensure amplicons in proximity to each other (i.e. less than 1 kilobase apart) are separated into different pools. To evaluate the robustness of PrimerPlex to design assays for multiplex bisulfite PCR, 178 primer pairs which had been previously screened were selected for multiplex pooling using the PP module; primer pairs which did not fulfil the sorting criteria are excluded from the multiplex analysis and are listed in a separate document. This created 8 pools of 178 pooled primers which were combined and used to amplify bisulfite-converted DNA. Agarose gel electrophoresis of the PCR product demonstrated the ability of PrimerPlex to successfully sort primers into different pools with no spurious PCR dimer product observed; representative gel image of the first 4 pools is shown in Fig. 4a. Next, the same 178 amplicons were assessed against a series of methylated controls (100%, 75%, 50%, 25% and 0%) prepared in duplicate, together with different cell line samples. Samples were then sequenced on an Illumina Miseq and the methylation level of each amplicon determined, as well as the extent to which it successfully targeted the region of interest. To determine the overall performance of the entire multiplex assay, the methylation state for every CpG was determined and a heat map was generated (Fig. 4b). Although a few sites showed unusual methylation patterns, the majority of CpG sites showed a level of methylation corresponding to the correct percentage of the related methylation control. Overall assay performance based on methylated controls indicated consistent performance across virtually all assays, even for amplicons which contained as many as 21 CpG sites (Fig. 4c). As the amplicons targeted regions that were observed to be involved in TNBC^7^, four breast cancer cell lines (MCF7, T-47D, MDA-MB-231 and MDA-MB-453) were also assayed, as well as a prostate cancer cell line (DuCap) and a sample of white blood cell DNA pooled from healthy individuals. As expected, these biological samples presented a complex methylation profile (Fig. 4d), and the complex methylation patterns observed over CpGs 1 to 8 likely represent the actual methylation state of the sample and not an artefact of amplification, as the methylated controls for the same region demonstrated a consistent performance free from bias. Interestingly, amplification of DNA derived from lymphoma B-cell lines (SU-DHL-4 and MINO) using amplicons targeting the same TNBC-associated regions also presented a complex methylation pattern across most of the regions analysed (Supplementary Figure S4), and suggests that these regions which were discovered in a breast-cancer specific biomarker project could have utility in the diagnosis of other cancers^30^,^31^.

**Figure 5:**
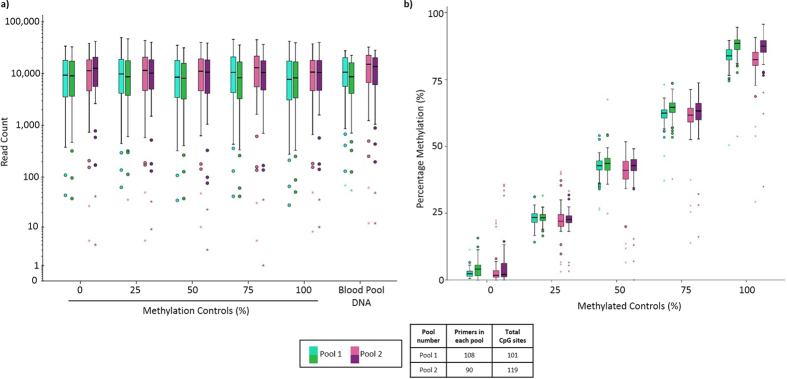
Representative sequencing results. Sequencing results of representative libraries prepared in duplicates for five methylated controls (0%, 25%, 50%, 75% and 100%) sequenced using primers generated and pooled using the PrimerSuite package using bisulfite multiplex PCR resequencing. (**a**) The number of reads per amplicon across each library in the individual pools. Read counts expressed in a log_10_ scale. (**b**) Summary of methylation results of each library across the set of methylated controls. Level of methylation was maintained at a consistent level corresponding to the baseline level of methylation across each methylated control.

Variation in the coverage of all 178 amplicons in the 8 pools was observed in the analysis (representative data shown in Fig. 5) although this issue can be rectified by optimising the concentration of each primer in the pool^6^. Furthermore, sequencing results of representative libraries showed the level of methylation of most CpGs corresponded to the baseline percentage of methylation of the corresponding methylated control sample screened, and presented an average mapping efficiency of 93% which indicated that the primers designed and pooled using the PrimerSuite package was highly robust for bisulfite multiplex PCR. Additionally, although no significant secondary products were observed during the initial multiplexing reaction, it is also possible that different artefacts (e.g. hairpins) may have been present but were not large enough to be visualised, which also could have contributed to the variance in amplicon coverage^32^. To control for this possibility, the PrimerDimer module was integrated into PrimerPlex so that dimer formation between primers in each pool is also considered during the multiplex pooling analysis. Furthermore, this feature has been implemented separately on the PrimerDimer website, to allow users to predict dimer formation between primers in pairs or in a multiplex pool.

### Construction of a validation primer set for the *Infinium HumanMethylation450 BeadChip* arrays

The Illumina *Infinium HumanMethylation450 BeadChip* array is used in epigenetic research to screen over 485,000 CpG sites per sample at a single nucleotide resolution, but many researchers often find it difficult to produce primers for validation assays which target these regions. To demonstrate the utility of the completed software package, a single FASTA file containing sequences covering the complete Illumina *Infinium HumanMethylation450 BeadChip* array was processed using PrimerSuite. Due to the close clustering of the CpGs in these regions and the exclusion of CpGs within the primers (PrimerSuite’s default parameter), PrimerSuite was only able to generate primers to 30% of the sites which are available for download as separate files in the Resources section of the website. However, by altering PrimerSuite’s default parameters users should be able to easily design primers against the remaining problematic regions.

## Conclusions

Identification and characterisation of CpG methylation using bisulfite-modified DNA is still the gold standard for analysis of 5-methlycytosine, but this requires the use of robust bisulfite-specific primers. However, a survey of current resources indicated a lack of automated solutions for high throughput primer design for bisulfite PCR, which prompted the creation of the PrimerSuite software package as detailed in this manuscript.

An interesting conclusion from this study was the statistical correlation between primers with a high number of primer-to-bisulfite-human-genome matches (p-value < 0.0022), which was nonetheless insufficient to accurately predict whether or not a primer pair would amplify specifically. However, mapping of primer pairs in paired-end mode against both the human genome and a reference index composed of repetitive bisulfite-converted sequences successfully identified primer pairs which were either predicted and/or observed to generate products from multiple parts of the genome. Although implementing paired-end mapping for all primers generated by PrimerSuite against the human genome is not a practical option for this web-based application, the use of a repeats library demonstrated suitable efficacy in identifying potentially problematic primers. Therefore, an optional check of primers against this repeat library is now implemented in PrimerSuite.

Finally, preliminary analysis of DNA derived from lymphoma B-cell lines (SU-DHL-4 and MINO) using amplicons associated with triple negative breast cancer presented a complex methylation pattern across most of the regions analysed despite these regions implicated as being specific to breast cancer. This suggests these regions could be used to differentiate between lymphoma and breast cancer, and potentially may have a deeper utility as diagnostic regions for other cancers.

In conclusion, this represents the first high-throughput web-based solution for bisulfite PCR and multiplex bisulfite PCR assays, and comparisons of PrimerSuite performance to other bisulfite primer programs demonstrated that PrimerSuite could successfully return primers for problematic regions which other applications failed at (Table 1). While this manuscript only contains data on the approximately 200 primer pairs, to date we have validated over 1300 different bisulfite-PCR assays (representative data in Fig. 5) in the wet lab to empirically demonstrate and confirm the functionalities of all three modules of the *PrimerSuite* program (PrimerSuite, PrimerDimer and PrimerPlex), and our conclusion is that this new primer design application is capable of generating robust primers for multiplex bisulfite PCR assays, and future improvements will explicitly support other applications such as methylation specific PCR and ligase chain reactions.

## Materials and Methods

### Review of current primer design software solutions

To determine whether each program fulfilled the criteria as outlined in Table 1, sequences from ten triple negative breast cancer (TNBC)-associated regions^7^ were parsed through a selection of currently-available software solutions for bisulfite primer design (Table 2).

### Implementation

The PrimerSuite software was originally written in the Python language (version 3.4+) (https://www.python.org/), and later adapted into a web application using the Django framework (version 1.8+) (https://www.djangoproject.com/), and hosted via Apache2 http server (https://httpd.apache.org/) on the nectar cloud (https://nectar.org.au/).

### Generation of primers for bisulfite PCR

To design primers for bisulfite-modified DNA, users are required to either ‘copy and paste’ their unmodified sequence(s) into the text area or upload a multi-FASTA file into PS. The program will generate two versions of the bisulfite-modified sequence(s), where one is the ‘C-T’ or Watson strand where all C’s are converted into ‘t’s, and all cytosines in a CpG context are marked with ‘N’; while the second strand -‘G-A’ or Crick strand has all non-CpG cytosines converted into ‘a’s. As the DNA strands are non-complementary post-conversion, PS generates and pairs primers from both strands using the salt-adjusted method^19^. Primers are selected based on a list of user-adjustable criteria and published in a downloadable excel workbook. The parameters section is prefilled with empirically-validated criteria as described in a previous study^6^. In this study, primers were designed against regions described in a previous breast cancer discovery study^7^ with the following key parameters: 0 CpG’s within the primers, at least 5 CpGs in the amplicon, amplicon size between 120–140 base pairs, oligonucleotide salt-adjusted melting temperature of 54 °C, and at least three unconverted bases (i.e., DNA bases which were not affected by the bisulfite conversion process) at the 3′ end of each primer. Primer pairs were selected based on the prospect of dimer formation between individual oligonucleotides as predicted by the PD module, which is embedded in the PS module.

### Prediction of Dimer formation between primer pairs

To predict potential primer dimer artefacts between two primers, users can either ‘copy and paste’ their primer sequences into the text area or upload a list of primers to be analysed. Dimerization can be calculated between primer pairs or between each primer in a multiplexed pool. In each case, the formation of dimers are calculated based on the Nearest Neighbour thermodynamics^18^,^22^,^23^,^24^,^25^,^26^,^27^,^28^.

### Prediction of optimal free energy at which dimer artefacts form

An optimal program was implemented in PD where users can predict the optimal free energy (ΔG) in different contexts. For this analysis, users are required to either ‘copy and paste’ their sequence, or upload their primer sequences (in FASTA format). Dimer analysis can be performed on individual primer pairs or multiplexed (where each primer is analysed against every other dimer in the file). The results can be downloaded either in an excel spreadsheet (where only the free energy calculations of each dimer formation is reported), or in a ‘dimer report’ (where the full structure of primer dimers with the most thermodynamically stable ΔG is reported in a text report (Additional File 1).

### Pooling of primers for multiplex bisulfite PCR using PrimerPlex

Independently validated primers were optimized using quantitative PCR (qPCR) using standard conditions. The different cycling threshold values (Ct) were obtained and entered into PP which pools primers pairs with proximal Ct values, and separates primer pairs into different pools based on different regions and the likelihood of primers to form dimers with one another.

### Bisulfite DNA conversions

Bisulfite conversion of template DNA was conducted using either manual protocols^3^, or using commercial kits (Human Genetic Signature MethylEasy Xceed, P/N ME002), as per the manufacturers protocols. For each conversion, DNA was first quantified with the Qubit dsDNA BR Assay Kit and, based on the available sample material, between 100 ng–1 ug of material was bisulfite converted at a time. Conversion took place at 80 °C for 45 minutes, followed by resuspension in low TE buffer (10 mM Tris-CL, pH 8.0, 0.1 mM EDTA).

### Bisulfite PCR Conditions

A PCR mastermix recipe for amplification of bisulfite-converted DNA was made and the final PCR reaction had the following components at the indicated concentrations: 5X Promega GoTaq 5X Green Flexibuffer (1X final, PN M5005), CES 5X, (0.5X final, **N**.**B**. refer to^33^ for CES recipe), MgCl_2_ (4.5 mM final), dNTP’s (200 uM each final), primers (forward and reverse at 100 mM final), Taq (0.025 U/uL final), DNA (variable final concentration, but typically not exceeding 2 ng/uL final concentration). Amplification took place on either an Eppendorf ProS 96 well, or Eppendorf Pro 384 well thermocylcer. For primers with an ‘oligo melting temperature of 54 °C, cycling conditions were: 94 °C, 5 mins; 12 cycles of (95 °C, 20 s; 60 °C, 1 min); 12 cycles of (94 °C, 20 s; 65 °C, 1 min 30 s); 65 °C, 3 min, 10 °C hold. PCR products were evaluated using standard agarose gel electrophoresis techniques with SB buffer.

### Barcoding PCR conditions

The final concentrations were: 1× Promega GoTaq Green Flexi buffer; 0.25 X CES; 4.5 mM MgCl2; 200 uM dNTPs; 0.05 U/uL HotStart Taq; 25 uL of pooled template after Agencourt XP bead cleanup; commercially purchased oligonucleotides with MiSeq sequencing adaptors and barcodes were used in the barcoding reaction (Fluidigm PN FLD-100-3771). Amplification took place on either an Eppendorf ProS 96 well, or Eppendorf Pro 384 well thermocylcer. Cycling conditions were: 94 °C, 5 mins; 9 cycles of (97 °C, 15 s; 60 °C, 30 s; 72 °C, 2 mins); 72 °C, 2 mins; 6 °C, 5 mins.

Sequencing was performed on the Illumina Miseq. MiSeq runs used the MiSeq Reagent Kit v2, 300 cycle; PN MS-102-2002.

### Bioinformatics

Adaptor trimming used Trim galore (options: -length 90). Mapping of sequenced data used the *Bismark*^34^ methylation mapping program running *Bowtie2*^35^ (options: –bowtie2 –N 1 -L 15 –bam -p 2 –score L, −0.6, −0.6 –non_directional;). To reduce computational overhead, sequences were mapped against only those genomic regions under investigation, plus an additional 100 bp of flanking sequence, unless otherwise noted. Graphing and statistical analysis employed SigmaPlot 12.5, JSM, and Excel 2010+ and SPSS.

To determine whether uniqueness of primers in the genome can predict PCR fidelity, the 100 primer pairs from trial one were mapped to a bisulfite-converted hg19 (http://hgdownload.soe.ucsc.edu/) and again to bisulfite-converted *Homo sapian* repeat sequences (http://www.girinst.org/repbase, accessed 18/02/2016). Mapping used Bowtie1 with the following options: -bowtie –n 0 –l 10 –sam –y –all. Statistical analysis was performed using Excel 2010+ and JMP Statistical Discovery platform.

### Availability

The PrimeSuite, PrimerDimer, and PrimerPlex modules are all available online at www.primersuite.com, www.primer-dimer.com, and www.primer-plex.com, respectively. An 80 MB file containing primer sequences designed by PrimerSuite against the regions covered in the *Infinium HumanMethylation450 BeadChip* array is also available for download from the PrimerSuite website through the ‘Help’ page, at www.primer-dimer.com/help/.

## Additional Information

**How to cite this article:** Lu, J. *et al*. PrimerSuite: A High-Throughput Web-Based Primer Design Program for Multiplex Bisulfite PCR. *Sci. Rep.* **7**, 41328; doi: 10.1038/srep41328 (2017).

**Publisher's note:** Springer Nature remains neutral with regard to jurisdictional claims in published maps and institutional affiliations.

## Supplementary Material

Supplementary Figures

Supplementary Additional File

## Figures and Tables

**Table 1:** Review of existing primer design programs.

	Multi-FASTA Input	Different Converted Bases	CpG adjustment		Homopolymer Assessment	Multiplex Pooling	Amplicon Size Adjustment	Selection between C-to-T or G-to-A converted genomes	Results Input	Dimer Predictions	Primers pairs@	Reference
			Primer	Amplicon								
PrimerSuite	Yes	Yes	Yes	Yes	Yes	Yes	Yes	Yes	Excel workbook	Yes	215+	This publication
Bisearch	No	No	No	No	No	No	No	No	Online view	Yes	19	Aranyi *et al*.^21^
Bisulfite Primer Seeker*	No	No	No	No	No	No	No	No	Email	No	2	zymoresearch.com
Epi-Designer#	Yes	No	No	Yes	Yes	No	Yes	Yes	Online view and excel export	No	2	epidesigner.com
BisPrimer	No	Yes	No	No	No	No	No	No	Reported as plain text	No	5	Kovacova *et al*.^10^
Meth-Primer	No	Yes	No	Yes	Yes	No	Yes	No	Online view	No	5	Li *et al*.^8^
Kismeth	Yes	Yes	No	No	No	No	Yes	Yes	Online View	No	3	Gruntman *et al*.^11^

^#^Adjustment of non-CpG cytosines.

^*^Allows 1 CpG in the first 1/3 of the primer.

^@^Number of primer pairs that has undergone empirical validation.

**Table 2:** Regions used for analysis of publically available primer design programs.

Chromosome regions*	Number of primer regions generated by each program						
	PrimerSuite	MethPrimer	Bisulfite Primer Seeker	Bisearch	Epidesigner	Bisprimer#	Kismeth
chr10:23527732-23529409	216	5	5	0	16	0	6
chr1:227635870-227637414	252	5	10	0	11	0	3
chr8:38705060-38705524	18	5	3	2	8	0	4
chr7:150736751-150738166	151	5	10	0	7	0	3
chr1:238321957-238322843	15	5	13	0	8	0	3
chr18:42589839-42591235	8	5	4	0	8	0	2
chr8:140783597-140784422	20	5	4	0	18	0	3
chr1:240753295-240754308	11	5	3	3	18	0	9
chr1:119329995-119332779	866	5	10	5	16	0	11
chr7:158629179-158630121	61	5	1	10	9	0	1

^*^All chromosomal regions extracted from human genome (hg18) for the original analysis.

^#^Bisprimer reported no primer pairs for any of the tested regions.

**Table 3:** Summary of statistical mean using Wilxcon signed rank test.

		Single gel band	Multiple gel bands	p
Human genome^#^	Single end	464.78	4556.18	0.0005
	Paired end	1.32	4.45	0.0022
Human repeats^#^	Single end	0.27	1.82	0.0004
	Paired end	N/A*	N/A*	N/A*

^*^Only one primer pair (C181 + C182) mapped 18 times in paired-end mode, producing amplicons varying in size between 29 bp to 219 bp (Fig. 3c); ^#^Reference genomes were bisulfite converted prior to mapping.
