# Supplementary material for: PrimerSuite: A High-Throughput Web-Based Primer Design Program for Multiplex Bisulfite PCR
Source: Sci Rep. 2017 Jan 24;7:41328. doi: 10.1038/srep41328 (PMC5259761; doi:10.1038/srep41328)
Supplement: Supplementary Figures [file srep41328-s1.pdf]

PrimerSuite: A High-Throughput Web-Based Primer Design Program for Multiplex Bisulfite PCR

Jennifer Lu, Andrew Johnston, Phillipe Berichon, Ke-lin Ru, Darren Korbie, Matt Trau

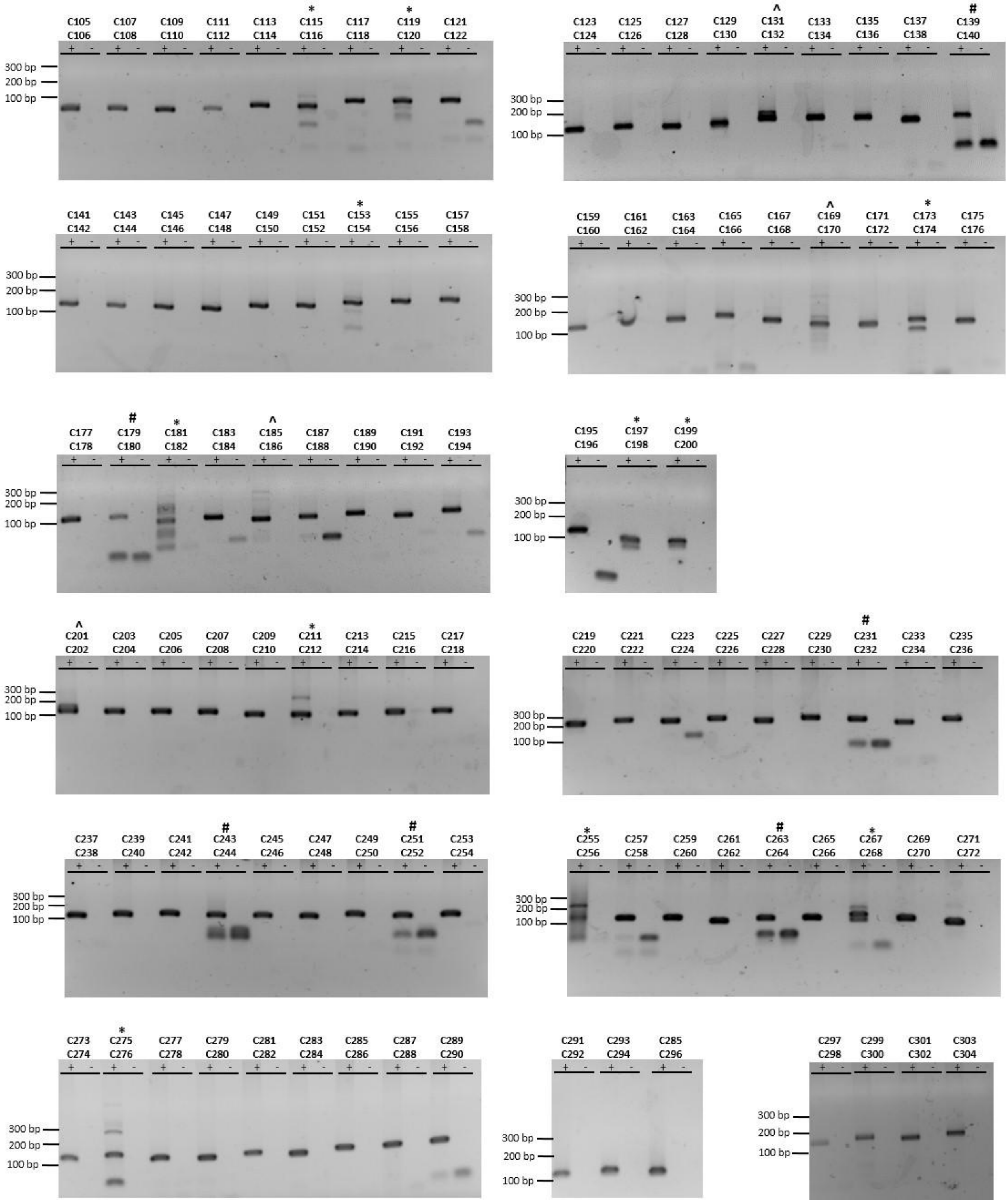

Supplementary Figure S1: Initial PrimerDimer primer pairs analysed using bisulfite PCR. Amplicons produced using PrimerSuite primers were observed to produce bands of the expected size (approximately 110-120base pairs in size), with six samples forming visible dimer products (#) and five with a secondary product (*), when visualised by agarose gel. In this study, dimers are classified where there is a band between 50 to 100 base pairs in size seen in both the positive and negative control. Bands of less than 50 bp in size are considered non-extension primers and does not interfere with amplification reaction.

PrimerSuite: A High-Throughput Web-Based Primer Design Program for Multiplex Bisulfite PCR

Jennifer Lu, Andrew Johnston, Phillipe Berichon, Ke-lin Ru, Darren Korbie, Matt Trau

A)

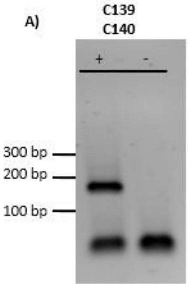

	Min ΔG (kcal/mol)
Heterodimer	-5.08
Top homodimer	7.4
Bottom homodimer	3.29

B)

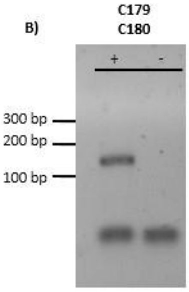

	Min ΔG (kcal/mol)
Heterodimer	-2.96
Top homodimer	3.92
Bottom homodimer	2.08

C)

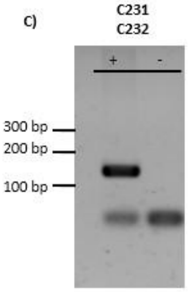

	Min ΔG (kcal/mol)
Heterodimer	-5.89
Top homodimer	4.6
Bottom homodimer	5.44

D)

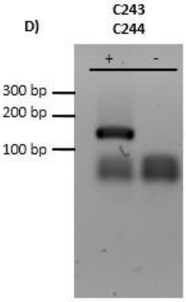

	Min ΔG (kcal/mol)
Heterodimer	-9.73
Top homodimer	6.71
Bottom homodimer	2.4

E)

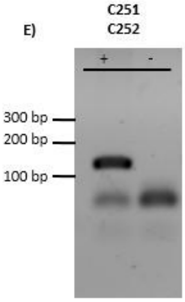

	Min ΔG (kcal/mol)
Heterodimer	-7.73
Top homodimer	4.57
Bottom homodimer	1.69

F)

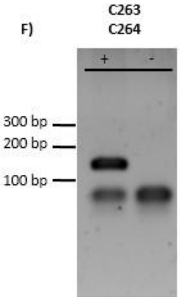

	Min ΔG (kcal/mol)
Heterodimer	-12.07
Top homodimer	0.64
Bottom homodimer	6.71

G)

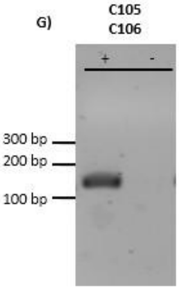

	Min ΔG (kcal/mol)
Heterodimer	-1.47
Top homodimer	0.58
Bottom homodimer	6.67

H)

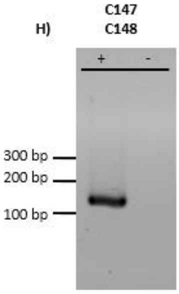

	Min ΔG (kcal/mol)
Heterodimer	-2.25
Top homodimer	4.0
Bottom homodimer	3.28

I)

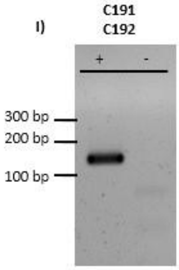

	Min ΔG (kcal/mol)
Heterodimer	-2.15
Top homodimer	5.19
Bottom homodimer	2.4

J)

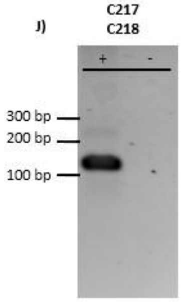

	Min ΔG (kcal/mol)
Heterodimer	-0.77
Top homodimer	1.93
Bottom homodimer	-1.39

K)

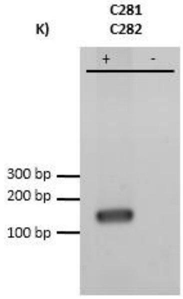

	Min ΔG (kcal/mol)
Heterodimer	-3.15
Top homodimer	-0.7
Bottom homodimer	-0.13

L)

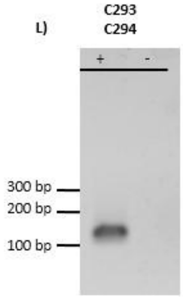

	Min ΔG (kcal/mol)
Heterodimer	-2.06
Top homodimer	1.18
Bottom homodimer	0.84

Supplementary Figure S2: Representative data of dimer formation between the primer and its reciprocal mate at the 3' end. The stability of each combination is reported as ΔG (kcal/mol). The six primer dimers pairs which produced dimers during the first validation of Primers Suite (**A-F**), was compared with six primer pairs which produced clean products (G-L) by examining both the structure of the dimer and the ΔG of each formation. All dimers were predicted using the updated Primer Dimer script. PrimerDimer output predicting the structures of the hetero- and homodimers for the dimer artefacts can be found in **Additional File 1**.

PrimerSuite: A High-Throughput Web-Based Primer Design Program for Multiplex Bisulfite PCR

Jennifer Lu, Andrew Johnston, Phillipe Berichon, Ke-lin Ru, Darren Korbie, Matt Trau

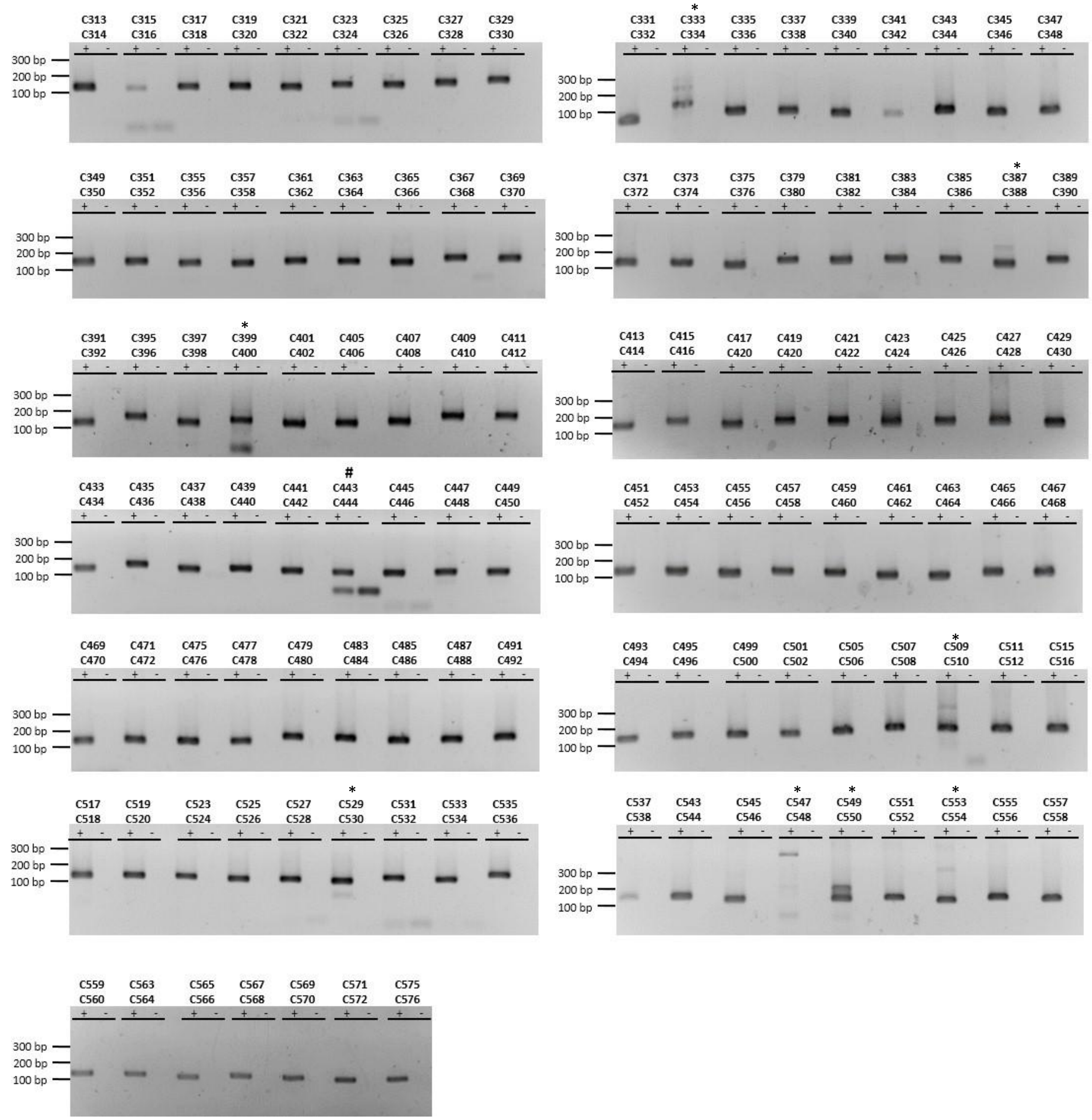

Supplementary Figure S3: Initial PrimerDimer primer pairs analyzed using bisulfite PCR. Amplicons produced using the optimized PrimerSuite primers were observed to produce bands of the expected size (approximately 110-120 base pairs in size), with only one primer pair forming a notable dimer (#). In this study, dimers are classified where there is a band between 50 to 100 base pairs in size seen in both the positive and negative control. Bands of less than 50 bp in size are considered non-extension primers and does not interfere with amplification reaction. Primer pairs which produced multiple products (*) were also excluded from further bisulfite multiplex screening.

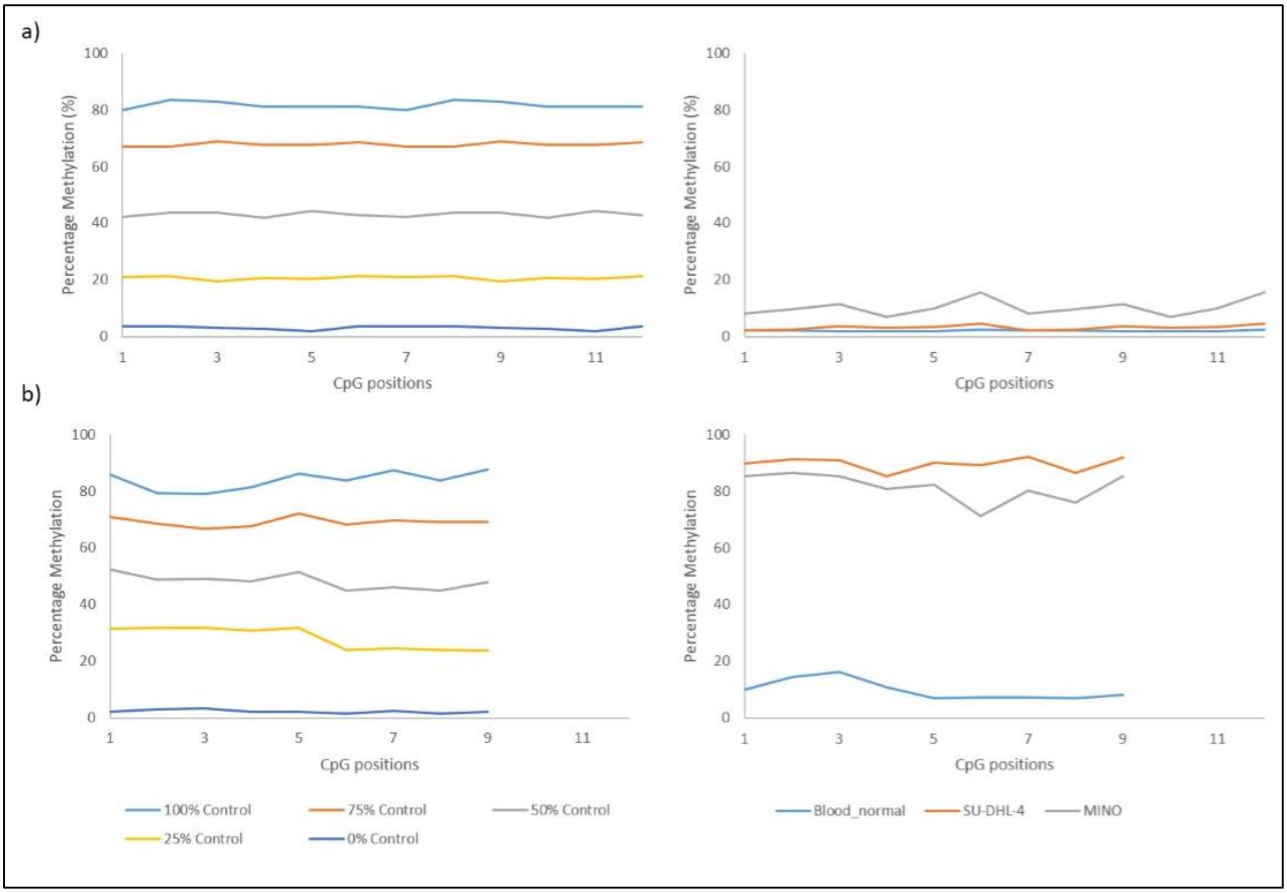

Supplementary Figure S4: Representative methylation profile. Representative result of two amplicons assayed are shown. Two lymphoma B cell lines (SU-DHL-4 and MINO), and blood normal were assayed in replicate, with a set of methylation controls (100 %, 75%, 50% 25% and 0% methylation controls). While amplicon (**a**) targeted a hypomethylated region, amplicon (**b**) targeted a hypermethylated region. In both instances, the level of methylation of the controls (**left**) were observed to be maintained at a consistent level across both the regions of interest, the methylation of the samples (**right**) presented a unique methylation pattern. On closer inspection, amplicon (**a**) covered an exon, while amplicon (**b**) amplified a portion of a CpG-island.

PrimerSuite: A High-Throughput Web-Based Primer Design Program for Multiplex Bisulfite PCR
Jennifer Lu, Andrew Johnston, Phillipe Berichon, Ke-lin Ru, Darren Korbie, Matt Trau

Additional File 1: List of the PrimerDimer output for primers shown Supplementary Figure 1.
