# Supplementary material for: PrimerSuite: A High-Throughput Web-Based Primer Design Program for Multiplex Bisulfite PCR
Source: Sci Rep. 2017 Jan 24;7:41328. doi: 10.1038/srep41328 (PMC5259761; doi:10.1038/srep41328)
Supplement: Supplementary Additional File [file srep41328-s2.pdf]

```
########################################################################################
#                                                                                      #
#                                  Primer Dimer Report                                 #
#                                                                                      #
########################################################################################
>>> - - - - - - - - - - - - - - - - - - - - - - - - - - - - - - - - - - - - - - -  <<<
        Min ΔG for C105 vs C106 is -1.47 kcal/mol.
    - - - - - - - - - - - - - - - - - - - - - - - - - - - - - - - - - - - - - - -
      5'> ATTGTGGGTAGTAAGGTATAAA >3'
             | |||         ||
         3'< CTCCCCAATACATCTAAAACT <5'
   - - - - - - - - - - - - - - - - - - - - - - - - - - - - - - - - - - - - - - -
        Min ΔG for C105 vs C105 is 0.58 kcal/mol.
    - - - - - - - - - - - - - - - - - - - - - - - - - - - - - - - - - - - - - - -
      5'> ATTGTGGGTAGTAAGGTATAAA >3'
                          ||||
                    3'< AAATATGGAATGATGGGTGTTA <5'
   - - - - - - - - - - - - - - - - - - - - - - - - - - - - - - - - - - - - - - -
        Min ΔG for C106 vs C106 is 6.67 kcal/mol.
    - - - - - - - - - - - - - - - - - - - - - - - - - - - - - - - - - - - - - - -
      5'> TCAAAATCTACATAACCCCTC >3'
                |       |
      3'< CTCCCCAATACATCTAAAACT <5'
   - - - - - - - - - - - - - - - - - - - - - - - - - - - - - - - - - - - - - - -
>>> - - - - - - - - - - - - - - - - - - - - - - - - - - - - - - - - - - - - - - -  <<<
****************************************************************************************
>>> - - - - - - - - - - - - - - - - - - - - - - - - - - - - - - - - - - - - - - -  <<<
        Min ΔG for C107 vs C108 is -1.81 kcal/mol.
    - - - - - - - - - - - - - - - - - - - - - - - - - - - - - - - - - - - - - - -
      5'> GTTATTGTTGAGAAATGGATTG >3'
              |||||         | |
        3'< TCAACAAAAAACATATTTAAAAAAACA <5'
   - - - - - - - - - - - - - - - - - - - - - - - - - - - - - - - - - - - - - - -
        Min ΔG for C107 vs C107 is 1.22 kcal/mol.
    - - - - - - - - - - - - - - - - - - - - - - - - - - - - - - - - - - - - - - -
      5'> GTTATTGTTGAGAAATGGATTG >3'
                       |||  |||
                  3'< GTTAGGTAAAGAGTTGTTATTG <5'
   - - - - - - - - - - - - - - - - - - - - - - - - - - - - - - - - - - - - - - -
        Min ΔG for C108 vs C108 is 1.74 kcal/mol.
    - - - - - - - - - - - - - - - - - - - - - - - - - - - - - - - - - - - - - - -
      5'> ACAAAAAAATTTATACAAAAAACAACT >3'
               |   ||| |   | |||     |
          3'< TCAACAAAAAACATATTTAAAAAAACA <5'
   - - - - - - - - - - - - - - - - - - - - - - - - - - - - - - - - - - - - - - -
>>> - - - - - - - - - - - - - - - - - - - - - - - - - - - - - - - - - - - - - - -  <<<
****************************************************************************************
>>> - - - - - - - - - - - - - - - - - - - - - - - - - - - - - - - - - - - - - - -  <<<
        Min ΔG for C109 vs C110 is -0.45 kcal/mol.
    - - - - - - - - - - - - - - - - - - - - - - - - - - - - - - - - - - - - - - -
      5'> GTGGTTATTGGATGGGTTTG >3'
                |  | |    |||
            3'< TTCCATTTAAAAAAAATCATTTCTAAA <5'
   - - - - - - - - - - - - - - - - - - - - - - - - - - - - - - - - - - - - - - -
        Min ΔG for C109 vs C109 is 5.43 kcal/mol.
    - - - - - - - - - - - - - - - - - - - - - - - - - - - - - - - - - - - - - - -
      5'> GTGGTTATTGGATGGGTTTG >3'
                      |    |
              3'< GTTTGGGTAGGTTATTGGTG <5'
   - - - - - - - - - - - - - - - - - - - - - - - - - - - - - - - - - - - - - - -
        Min ΔG for C110 vs C110 is -0.44 kcal/mol.
    - - - - - - - - - - - - - - - - - - - - - - - - - - - - - - - - - - - - - - -
      5'> AAATCTTTACTAAAAAAAATTTACCTT >3'
                     ||   ||||||   ||
                 3'< TTCCATTTAAAAAAAATCATTTCTAAA <5'
   - - - - - - - - - - - - - - - - - - - - - - - - - - - - - - - - - - - - - - -
>>> - - - - - - - - - - - - - - - - - - - - - - - - - - - - - - - - - - - - - - -  <<<
```

```
****************************************************************************************
>>> - - - - - - - - - - - - - - - - - - - - - - - - - - - - - - - - - - - - - - - -  <<<
        Min ΔG for C111 vs C112 is -3.31 kcal/mol.
    - - - - - - - - - - - - - - - - - - - - - - - - - - - - - - - - - - - - - - - -
      5'> ACTACCCTCAACTTCCCAA >3'
            | | ||||||  |||||
      3'< GTAAGTGAGTTGTTGGGTTT <5'
   - - - - - - - - - - - - - - - - - - - - - - - - - - - - - - - - - - - - - - - -
        Min ΔG for C111 vs C111 is 1.8 kcal/mol.
    - - - - - - - - - - - - - - - - - - - - - - - - - - - - - - - - - - - - - - - -
      5'> ACTACCCTCAACTTCCCAA >3'
                      ||   ||
                  3'< AACCCTTCAACTCCCATCA <5'
   - - - - - - - - - - - - - - - - - - - - - - - - - - - - - - - - - - - - - - - -
        Min ΔG for C112 vs C112 is 2.1 kcal/mol.
    - - - - - - - - - - - - - - - - - - - - - - - - - - - - - - - - - - - - - - - -
      5'> TTTGGGTTGTTGAGTGAATG >3'
                           ||
                      3'< GTAAGTGAGTTGTTGGGTTT <5'
    - - - - - - - - - - - - - - - - - - - - - - - - - - - - - - - - - - - - - - - -
>>> - - - - - - - - - - - - - - - - - - - - - - - - - - - - - - - - - - - - - - - -  <<<
****************************************************************************************
>>> - - - - - - - - - - - - - - - - - - - - - - - - - - - - - - - - - - - - - - - -  <<<
        Min ΔG for C113 vs C114 is -2.37 kcal/mol.
    - - - - - - - - - - - - - - - - - - - - - - - - - - - - - - - - - - - - - - - -
      5'> TGTTTTTTTTTTTGTTGTGGGTAA >3'
                           || || |
                       3'< ACTCAATTCATAATCCTAAATATC <5'
   - - - - - - - - - - - - - - - - - - - - - - - - - - - - - - - - - - - - - - - -
        Min ΔG for C113 vs C113 is 1.18 kcal/mol.
    - - - - - - - - - - - - - - - - - - - - - - - - - - - - - - - - - - - - - - - -
      5'> TGTTTTTTTTTTTGTTGTGGGTAA >3'
                               ||
                          3'< AATGGGTGTTGTTTTTTTTTTTGT <5'
   - - - - - - - - - - - - - - - - - - - - - - - - - - - - - - - - - - - - - - - -
        Min ΔG for C114 vs C114 is 2.08 kcal/mol.
    - - - - - - - - - - - - - - - - - - - - - - - - - - - - - - - - - - - - - - - -
      5'> CTATAAATCCTAATACTTAACTCA >3'
                               | |
                           3'< ACTCAATTCATAATCCTAAATATC <5'
    - - - - - - - - - - - - - - - - - - - - - - - - - - - - - - - - - - - - - - - -
>>> - - - - - - - - - - - - - - - - - - - - - - - - - - - - - - - - - - - - - - - -  <<<
****************************************************************************************
>>> - - - - - - - - - - - - - - - - - - - - - - - - - - - - - - - - - - - - - - - -  <<<
        Min ΔG for C115 vs C116 is -3.85 kcal/mol.
    - - - - - - - - - - - - - - - - - - - - - - - - - - - - - - - - - - - - - - - -
      5'> TTTAGAGAAAAGTAGGTTGTGG >3'
                         || || |
                     3'< CATCATCTCCAAAAACCAAAC <5'
   - - - - - - - - - - - - - - - - - - - - - - - - - - - - - - - - - - - - - - - -
        Min ΔG for C115 vs C115 is 2.25 kcal/mol.
    - - - - - - - - - - - - - - - - - - - - - - - - - - - - - - - - - - - - - - - -
      5'> TTTAGAGAAAAGTAGGTTGTGG >3'
               | ||       || |
         3'< GGTGTTGGATGAAAAGAGATTT <5'
   - - - - - - - - - - - - - - - - - - - - - - - - - - - - - - - - - - - - - - - -
        Min ΔG for C116 vs C116 is 3.56 kcal/mol.
    - - - - - - - - - - - - - - - - - - - - - - - - - - - - - - - - - - - - - - - -
      5'> CAAACCAAAAACCTCTACTAC >3'
                            ||
                       3'< CATCATCTCCAAAAACCAAAC <5'
   - - - - - - - - - - - - - - - - - - - - - - - - - - - - - - - - - - - - - - - -
>>> - - - - - - - - - - - - - - - - - - - - - - - - - - - - - - - - - - - - - - - -  <<<
****************************************************************************************
>>> - - - - - - - - - - - - - - - - - - - - - - - - - - - - - - - - - - - - - - - -  <<<
        Min ΔG for C117 vs C118 is -1.89 kcal/mol.
    - - - - - - - - - - - - - - - - - - - - - - - - - - - - - - - - - - - - - - - -
      5'> AACTTTACAATTTTTATTACTACTAC >3'
                                ||| 
                            3'< AATGGGTGGTGGGGAGA <5'
   - - - - - - - - - - - - - - - - - - - - - - - - - - - - - - - - - - - - - - - -
        Min ΔG for C117 vs C117 is 3.56 kcal/mol.
    - - - - - - - - - - - - - - - - - - - - - - - - - - - - - - - - - - - - - - - -
      5'> AACTTTACAATTTTTATTACTACTAC >3'
                                ||
                            3'< CATCATCATTATTTTTAACATTTCAA <5'
   - - - - - - - - - - - - - - - - - - - - - - - - - - - - - - - - - - - - - - - -
        Min ΔG for C118 vs C118 is 1.18 kcal/mol.
    - - - - - - - - - - - - - - - - - - - - - - - - - - - - - - - - - - - - - - - -
      5'> AGAGGGGTGGTGGGTAA >3'
                        ||
                   3'< AATGGGTGGTGGGGAGA <5'
   - - - - - - - - - - - - - - - - - - - - - - - - - - - - - - - - - - - - - - - -
>>> - - - - - - - - - - - - - - - - - - - - - - - - - - - - - - - - - - - - - - - -  <<<
```

```
*****************************************************************************************
>>> - - - - - - - - - - - - - - - - - - - - - - - - - - - - - - - - - - - - - - - -  <<<
        Min ΔG for C119 vs C120 is -3.07 kcal/mol.
    - - - - - - - - - - - - - - - - - - - - - - - - - - - - - - - - - - - - - - - -
      5'> GGTAAGTGTTTAGGATGGTT >3'
                      ||| || |
               3'< TTCCCTCCCCAAAACACTA <5'
   - - - - - - - - - - - - - - - - - - - - - - - - - - - - - - - - - - - - - - - -
        Min ΔG for C119 vs C119 is 0.31 kcal/mol.
    - - - - - - - - - - - - - - - - - - - - - - - - - - - - - - - - - - - - - - - -
      5'> GGTAAGTGTTTAGGATGGTT >3'
             ||   |     |   ||
         3'< TTGGTAGGATTTGTGAATGG <5'
   - - - - - - - - - - - - - - - - - - - - - - - - - - - - - - - - - - - - - - - -
        Min ΔG for C120 vs C120 is 2.87 kcal/mol.
    - - - - - - - - - - - - - - - - - - - - - - - - - - - - - - - - - - - - - - - -
      5'> ATCACAAAACCCCTCCCTT >3'
                ||         ||
            3'< TTCCCTCCCCAAAACACTA <5'
   - - - - - - - - - - - - - - - - - - - - - - - - - - - - - - - - - - - - - - - -
>>> - - - - - - - - - - - - - - - - - - - - - - - - - - - - - - - - - - - - - - - -  <<<
*****************************************************************************************
>>> - - - - - - - - - - - - - - - - - - - - - - - - - - - - - - - - - - - - - - - -  <<<
        Min ΔG for C121 vs C122 is -6.0 kcal/mol.
    - - - - - - - - - - - - - - - - - - - - - - - - - - - - - - - - - - - - - - - -
      5'> AAACCACTTTACTCTTCCCT >3'
                         |||||
                    3'< GAGGGATATAAGTTATGGTTAT <5'
   - - - - - - - - - - - - - - - - - - - - - - - - - - - - - - - - - - - - - - - -
        Min ΔG for C121 vs C121 is 4.7 kcal/mol.
    - - - - - - - - - - - - - - - - - - - - - - - - - - - - - - - - - - - - - - - -
      5'> AAACCACTTTACTCTTCCCT >3'
           |   |         |   |
       3'< TCCCTTCTCATTTCACCAAA <5'
   - - - - - - - - - - - - - - - - - - - - - - - - - - - - - - - - - - - - - - - -
        Min ΔG for C122 vs C122 is 2.01 kcal/mol.
    - - - - - - - - - - - - - - - - - - - - - - - - - - - - - - - - - - - - - - - -
      5'> TATTGGTATTGAATATAGGGAG >3'
            |    |||    |||   |
       3'< GAGGGATATAAGTTATGGTTAT <5'
   - - - - - - - - - - - - - - - - - - - - - - - - - - - - - - - - - - - - - - - -
>>> - - - - - - - - - - - - - - - - - - - - - - - - - - - - - - - - - - - - - - - -  <<<
*****************************************************************************************
>>> - - - - - - - - - - - - - - - - - - - - - - - - - - - - - - - - - - - - - - - -  <<<
        Min ΔG for C123 vs C124 is -2.81 kcal/mol.
    - - - - - - - - - - - - - - - - - - - - - - - - - - - - - - - - - - - - - - - -
      5'> CATCCTTAAAAATACTCTTTCC >3'
             ||| ||| ||  |      |
        3'< GGGAGTTTATAATTGGGTTG <5'
   - - - - - - - - - - - - - - - - - - - - - - - - - - - - - - - - - - - - - - - -
        Min ΔG for C123 vs C123 is 3.37 kcal/mol.
    - - - - - - - - - - - - - - - - - - - - - - - - - - - - - - - - - - - - - - - -
      5'> CATCCTTAAAAATACTCTTTCC >3'
                   ||| | | |||
             3'< CCTTTCTCATAAAAATTCCTAC <5'
   - - - - - - - - - - - - - - - - - - - - - - - - - - - - - - - - - - - - - - - -
        Min ΔG for C124 vs C124 is 2.88 kcal/mol.
    - - - - - - - - - - - - - - - - - - - - - - - - - - - - - - - - - - - - - - - -
      5'> GTTGGGTTAATATTTGAGGG >3'
                | ||    || |
         3'< GGGAGTTTATAATTGGGTTG <5'
   - - - - - - - - - - - - - - - - - - - - - - - - - - - - - - - - - - - - - - - -
>>> - - - - - - - - - - - - - - - - - - - - - - - - - - - - - - - - - - - - - - - -  <<<
*****************************************************************************************
>>> - - - - - - - - - - - - - - - - - - - - - - - - - - - - - - - - - - - - - - - -  <<<
        Min ΔG for C125 vs C126 is -4.31 kcal/mol.
    - - - - - - - - - - - - - - - - - - - - - - - - - - - - - - - - - - - - - - - -
      5'> ATAATCTCTCTAAACTCTCCAA >3'
                       ||| | |||
                  3'< ATGAAATGTTGGTTTTTTTTGGAT <5'
   - - - - - - - - - - - - - - - - - - - - - - - - - - - - - - - - - - - - - - - -
        Min ΔG for C125 vs C125 is 4.4 kcal/mol.
    - - - - - - - - - - - - - - - - - - - - - - - - - - - - - - - - - - - - - - - -
      5'> ATAATCTCTCTAAACTCTCCAA >3'
                           |   |
                       3'< AACCTCTCAAATCTCTCTAATA <5'
   - - - - - - - - - - - - - - - - - - - - - - - - - - - - - - - - - - - - - - - -
        Min ΔG for C126 vs C126 is -0.6 kcal/mol.
    - - - - - - - - - - - - - - - - - - - - - - - - - - - - - - - - - - - - - - - -
      5'> TAGGTTTTTTTTGGTTGTAAAGTA >3'
                                ||
                            3'< ATGAAATGTTGGTTTTTTTTGGAT <5'
   - - - - - - - - - - - - - - - - - - - - - - - - - - - - - - - - - - - - - - - -
>>> - - - - - - - - - - - - - - - - - - - - - - - - - - - - - - - - - - - - - - - -  <<<
```

```
****************************************************************************************
>>> - - - - - - - - - - - - - - - - - - - - - - - - - - - - - - - - - - - - - - - -  <<<
        Min ΔG for C127 vs C128 is -2.41 kcal/mol.
    - - - - - - - - - - - - - - - - - - - - - - - - - - - - - - - - - - - - - - - -
      5'> CCCAAAACACCCCCACT >3'
                     ||  ||
               3'< TTGGAAGAAGAGAAATTGGG <5'
   - - - - - - - - - - - - - - - - - - - - - - - - - - - - - - - - - - - - - - - -
        Min ΔG for C127 vs C127 is 2.24 kcal/mol.
    - - - - - - - - - - - - - - - - - - - - - - - - - - - - - - - - - - - - - - - -
      5'> CCCAAAACACCCCCACT >3'
                        | |
                    3'< TCACCCCCACAAAACCC <5'
   - - - - - - - - - - - - - - - - - - - - - - - - - - - - - - - - - - - - - - - -
        Min ΔG for C128 vs C128 is -2.04 kcal/mol.
    - - - - - - - - - - - - - - - - - - - - - - - - - - - - - - - - - - - - - - - -
      5'> GGGTTAAAGAGAAGAAGGTT >3'
                        ||  ||
                    3'< TTGGAAGAAGAGAAATTGGG <5'
   - - - - - - - - - - - - - - - - - - - - - - - - - - - - - - - - - - - - - - - -
>>> - - - - - - - - - - - - - - - - - - - - - - - - - - - - - - - - - - - - - - - -  <<<
****************************************************************************************
>>> - - - - - - - - - - - - - - - - - - - - - - - - - - - - - - - - - - - - - - - -  <<<
        Min ΔG for C129 vs C130 is -3.49 kcal/mol.
    - - - - - - - - - - - - - - - - - - - - - - - - - - - - - - - - - - - - - - - -
      5'> TAATCACATCTTCTATCACTCA >3'
                          || |||
                    3'< AGGTTAGTTTGTGTGTTGATTT <5'
   - - - - - - - - - - - - - - - - - - - - - - - - - - - - - - - - - - - - - - - -
        Min ΔG for C129 vs C129 is 2.08 kcal/mol.
    - - - - - - - - - - - - - - - - - - - - - - - - - - - - - - - - - - - - - - - -
      5'> TAATCACATCTTCTATCACTCA >3'
                             | |
                         3'< ACTCACTATCTTCTACACTAAT <5'
   - - - - - - - - - - - - - - - - - - - - - - - - - - - - - - - - - - - - - - - -
        Min ΔG for C130 vs C130 is 1.66 kcal/mol.
    - - - - - - - - - - - - - - - - - - - - - - - - - - - - - - - - - - - - - - - -
      5'> TTTAGTTGTGTGTTTGATTGGA >3'
                            |  |
                        3'< AGGTTAGTTTGTGTGTTGATTT <5'
   - - - - - - - - - - - - - - - - - - - - - - - - - - - - - - - - - - - - - - - -
>>> - - - - - - - - - - - - - - - - - - - - - - - - - - - - - - - - - - - - - - - -  <<<
****************************************************************************************
>>> - - - - - - - - - - - - - - - - - - - - - - - - - - - - - - - - - - - - - - - -  <<<
        Min ΔG for C131 vs C132 is 0.36 kcal/mol.
    - - - - - - - - - - - - - - - - - - - - - - - - - - - - - - - - - - - - - - - -
      5'> AAACTATCCAATCAAACACACA >3'
                            |||
                     3'< GAAGTGATGAGATATAGGAG <5'
   - - - - - - - - - - - - - - - - - - - - - - - - - - - - - - - - - - - - - - - -
        Min ΔG for C131 vs C131 is 4.95 kcal/mol.
    - - - - - - - - - - - - - - - - - - - - - - - - - - - - - - - - - - - - - - - -
      5'> AAACTATCCAATCAAACACACA >3'
                |              |
            3'< ACACACAAACTAACCTATCAAA <5'
   - - - - - - - - - - - - - - - - - - - - - - - - - - - - - - - - - - - - - - - -
        Min ΔG for C132 vs C132 is 2.67 kcal/mol.
    - - - - - - - - - - - - - - - - - - - - - - - - - - - - - - - - - - - - - - - -
      5'> GAGGATATAGAGTAGTGAAG >3'
                 |  | || |  |
            3'< GAAGTGATGAGATATAGGAG <5'
   - - - - - - - - - - - - - - - - - - - - - - - - - - - - - - - - - - - - - - - -
>>> - - - - - - - - - - - - - - - - - - - - - - - - - - - - - - - - - - - - - - - -  <<<
****************************************************************************************
>>> - - - - - - - - - - - - - - - - - - - - - - - - - - - - - - - - - - - - - - - -  <<<
        Min ΔG for C133 vs C134 is -4.98 kcal/mol.
    - - - - - - - - - - - - - - - - - - - - - - - - - - - - - - - - - - - - - - - -
      5'> GGAAATTAGGGATTATGAGG >3'
                   || ||  ||||
              3'< TCCCAACCCTCCCAAAAAT <5'
   - - - - - - - - - - - - - - - - - - - - - - - - - - - - - - - - - - - - - - - -
        Min ΔG for C133 vs C133 is 2.72 kcal/mol.
    - - - - - - - - - - - - - - - - - - - - - - - - - - - - - - - - - - - - - - - -
      5'> GGAAATTAGGGATTATGAGG >3'
                      | || |
                3'< GGAGTATTAGGGATTAAAGG <5'
   - - - - - - - - - - - - - - - - - - - - - - - - - - - - - - - - - - - - - - - -
        Min ΔG for C134 vs C134 is 3.91 kcal/mol.
    - - - - - - - - - - - - - - - - - - - - - - - - - - - - - - - - - - - - - - - -
      5'> TAAAAACCCTCCCAACCCT >3'
                        |   |
                    3'< TCCCAACCCTCCCAAAAAT <5'
   - - - - - - - - - - - - - - - - - - - - - - - - - - - - - - - - - - - - - - - -
>>> - - - - - - - - - - - - - - - - - - - - - - - - - - - - - - - - - - - - - - - -  <<<
```

```
****************************************************************************************
>>> - - - - - - - - - - - - - - - - - - - - - - - - - - - - - - - - - - - - - - - -  <<<
        Min ΔG for C135 vs C136 is -3.65 kcal/mol.
    - - - - - - - - - - - - - - - - - - - - - - - - - - - - - - - - - - - - - - -
      5'> AATAGGTAGATTTTTTTAGGAGAG >3'
                          || |||
                      3'< TCTTCTAAACTACACATCCC <5'
   - - - - - - - - - - - - - - - - - - - - - - - - - - - - - - - - - - - - - - -
        Min ΔG for C135 vs C135 is 3.9 kcal/mol.
    - - - - - - - - - - - - - - - - - - - - - - - - - - - - - - - - - - - - - - -
      5'> AATAGGTAGATTTTTTTAGGAGAG >3'
                     | |  ||  | |
               3'< GAGAGGATTTTTTTAGATGGATAA <5'
   - - - - - - - - - - - - - - - - - - - - - - - - - - - - - - - - - - - - - - -
        Min ΔG for C136 vs C136 is 3.27 kcal/mol.
    - - - - - - - - - - - - - - - - - - - - - - - - - - - - - - - - - - - - - - -
      5'> CCCTACACATCAAATCTTCT >3'
                  |  | || |  |
              3'< TCTTCTAAACTACACATCCC <5'
   - - - - - - - - - - - - - - - - - - - - - - - - - - - - - - - - - - - - - - -
>>> - - - - - - - - - - - - - - - - - - - - - - - - - - - - - - - - - - - - - - - -  <<<
****************************************************************************************
>>> - - - - - - - - - - - - - - - - - - - - - - - - - - - - - - - - - - - - - - - -  <<<
        Min ΔG for C137 vs C138 is -2.77 kcal/mol.
    - - - - - - - - - - - - - - - - - - - - - - - - - - - - - - - - - - - - - - -
      5'> TCAAACTCCTTACTCTATTCTC >3'
                  |   |     || ||
          3'< AAGGATGTTTTGAAATAGGATTTA <5'
   - - - - - - - - - - - - - - - - - - - - - - - - - - - - - - - - - - - - - - -
        Min ΔG for C137 vs C137 is 4.62 kcal/mol.
    - - - - - - - - - - - - - - - - - - - - - - - - - - - - - - - - - - - - - - -
      5'> TCAAACTCCTTACTCTATTCTC >3'
                          ||
                  3'< CTCTTATCTCATTCCTCAAACT <5'
   - - - - - - - - - - - - - - - - - - - - - - - - - - - - - - - - - - - - - - -
        Min ΔG for C138 vs C138 is -0.76 kcal/mol.
    - - - - - - - - - - - - - - - - - - - - - - - - - - - - - - - - - - - - - - -
      5'> ATTTAGGATAAAGTTTTGTAGGAA >3'
                        ||  ||  ||
                    3'< AAGGATGTTTTGAAATAGGATTTA <5'
   - - - - - - - - - - - - - - - - - - - - - - - - - - - - - - - - - - - - - - -
>>> - - - - - - - - - - - - - - - - - - - - - - - - - - - - - - - - - - - - - - - -  <<<
****************************************************************************************
>>> - - - - - - - - - - - - - - - - - - - - - - - - - - - - - - - - - - - - - - - -  <<<
        Min ΔG for C139 vs C140 is -5.08 kcal/mol.
    - - - - - - - - - - - - - - - - - - - - - - - - - - - - - - - - - - - - - - -
      5'> ACTCCCTCCCCACCC >3'
                  |||||
              3'< GGGTGTTTGTTTTTTAAGAATG <5'
   - - - - - - - - - - - - - - - - - - - - - - - - - - - - - - - - - - - - - - -
        Min ΔG for C139 vs C139 is 7.4 kcal/mol.
    - - - - - - - - - - - - - - - - - - - - - - - - - - - - - - - - - - - - - - -
      5'> ACTCCCTCCCCACCC >3'
                |    |
         3'< CCCACCCCTCCCTCA <5'
   - - - - - - - - - - - - - - - - - - - - - - - - - - - - - - - - - - - - - - -
        Min ΔG for C140 vs C140 is 3.29 kcal/mol.
    - - - - - - - - - - - - - - - - - - - - - - - - - - - - - - - - - - - - - - -
      5'> GTAAGAATTTTTTGTTTGTGGG >3'
             | ||         || |
      3'< GGGTGTTTGTTTTTTAAGAATG <5'
   - - - - - - - - - - - - - - - - - - - - - - - - - - - - - - - - - - - - - - -
>>> - - - - - - - - - - - - - - - - - - - - - - - - - - - - - - - - - - - - - - - -  <<<
****************************************************************************************
>>> - - - - - - - - - - - - - - - - - - - - - - - - - - - - - - - - - - - - - - - -  <<<
        Min ΔG for C141 vs C142 is -4.41 kcal/mol.
    - - - - - - - - - - - - - - - - - - - - - - - - - - - - - - - - - - - - - - -
      5'> TATTCTAAATTCTTCCTTTTCCC >3'
                      ||   ||| ||
                 3'< TAATATAAATGGATTTTTTAGTTTTTG <5'
   - - - - - - - - - - - - - - - - - - - - - - - - - - - - - - - - - - - - - - -
        Min ΔG for C141 vs C141 is 2.78 kcal/mol.
    - - - - - - - - - - - - - - - - - - - - - - - - - - - - - - - - - - - - - - -
      5'> TATTCTAAATTCTTCCTTTTCCC >3'
                |||       |||
        3'< CCCTTTTCCTTCTTAAATCTTAT <5'
   - - - - - - - - - - - - - - - - - - - - - - - - - - - - - - - - - - - - - - -
        Min ΔG for C142 vs C142 is -1.39 kcal/mol.
    - - - - - - - - - - - - - - - - - - - - - - - - - - - - - - - - - - - - - - -
      5'> GTTTTTGATTTTTTAGGTAAATATAAT >3'
                                   ||
                               3'< TAATATAAATGGATTTTTTAGTTTTTG <5'
   - - - - - - - - - - - - - - - - - - - - - - - - - - - - - - - - - - - - - - -
>>> - - - - - - - - - - - - - - - - - - - - - - - - - - - - - - - - - - - - - - - -  <<<
```

```
****************************************************************************************
>>> - - - - - - - - - - - - - - - - - - - - - - - - - - - - - - - - - - - - - - - -  <<<
        Min ΔG for C143 vs C144 is -2.3 kcal/mol.
    - - - - - - - - - - - - - - - - - - - - - - - - - - - - - - - - - - - - - - - -
      5'> TTAGAGTTATTTTTTTTAGTTAGGA >3'
            ||||| | ||  || | |
      3'< CTTCTCATTCAATCAACTTACA <5'
   - - - - - - - - - - - - - - - - - - - - - - - - - - - - - - - - - - - - - - - -
        Min ΔG for C143 vs C143 is 2.3 kcal/mol.
    - - - - - - - - - - - - - - - - - - - - - - - - - - - - - - - - - - - - - - - -
      5'> TTAGAGTTATTTTTTTTAGTTAGGA >3'
                          |  | |  |
                      3'< AGGATTGATTTTTTTTATTGAGATT <5'
   - - - - - - - - - - - - - - - - - - - - - - - - - - - - - - - - - - - - - - - -
        Min ΔG for C144 vs C144 is 5.42 kcal/mol.
    - - - - - - - - - - - - - - - - - - - - - - - - - - - - - - - - - - - - - - - -
      5'> ACATTCAACTAACTTACTCTTC >3'
                    ||       ||
               3'< CTTCTCATTCAATCAACTTACA <5'
   - - - - - - - - - - - - - - - - - - - - - - - - - - - - - - - - - - - - - - - -
>>> - - - - - - - - - - - - - - - - - - - - - - - - - - - - - - - - - - - - - - - -  <<<
****************************************************************************************
>>> - - - - - - - - - - - - - - - - - - - - - - - - - - - - - - - - - - - - - - - -  <<<
        Min ΔG for C145 vs C146 is -1.53 kcal/mol.
    - - - - - - - - - - - - - - - - - - - - - - - - - - - - - - - - - - - - - - - -
      5'> GAAGTTAAATTTAATATGGATTTGG >3'
                     |||||  | || ||
                 3'< ATTATTACCAACCCAACCTC <5'
   - - - - - - - - - - - - - - - - - - - - - - - - - - - - - - - - - - - - - - - -
        Min ΔG for C145 vs C145 is 0.23 kcal/mol.
    - - - - - - - - - - - - - - - - - - - - - - - - - - - - - - - - - - - - - - - -
      5'> GAAGTTAAATTTAATATGGATTTGG >3'
                ||||  |   |  ||||
          3'< GGTTTAGGTATAATTTAAATTGAAG <5'
   - - - - - - - - - - - - - - - - - - - - - - - - - - - - - - - - - - - - - - - -
        Min ΔG for C146 vs C146 is -0.7 kcal/mol.
    - - - - - - - - - - - - - - - - - - - - - - - - - - - - - - - - - - - - - - - -
      5'> CTCCAACCCAACCATTATTA >3'
                            ||
                        3'< ATTATTACCAACCCAACCTC <5'
   - - - - - - - - - - - - - - - - - - - - - - - - - - - - - - - - - - - - - - - -
>>> - - - - - - - - - - - - - - - - - - - - - - - - - - - - - - - - - - - - - - - -  <<<
****************************************************************************************
>>> - - - - - - - - - - - - - - - - - - - - - - - - - - - - - - - - - - - - - - - -  <<<
        Min ΔG for C147 vs C148 is -2.25 kcal/mol.
    - - - - - - - - - - - - - - - - - - - - - - - - - - - - - - - - - - - - - - - -
      5'> CACTCAAATCACCCACTCT >3'
                         | ||
                     3'< GGGATGTTATAGGGGATGT <5'
   - - - - - - - - - - - - - - - - - - - - - - - - - - - - - - - - - - - - - - - -
        Min ΔG for C147 vs C147 is 4.0 kcal/mol.
    - - - - - - - - - - - - - - - - - - - - - - - - - - - - - - - - - - - - - - - -
      5'> CACTCAAATCACCCACTCT >3'
                        |   |
                    3'< TCTCACCCACTAAACTCAC <5'
   - - - - - - - - - - - - - - - - - - - - - - - - - - - - - - - - - - - - - - - -
        Min ΔG for C148 vs C148 is 3.28 kcal/mol.
    - - - - - - - - - - - - - - - - - - - - - - - - - - - - - - - - - - - - - - - -
      5'> TGTAGGGGATATTGTAGGG >3'
                        ||
                 3'< GGGATGTTATAGGGGATGT <5'
   - - - - - - - - - - - - - - - - - - - - - - - - - - - - - - - - - - - - - - - -
>>> - - - - - - - - - - - - - - - - - - - - - - - - - - - - - - - - - - - - - - - -  <<<
****************************************************************************************
>>> - - - - - - - - - - - - - - - - - - - - - - - - - - - - - - - - - - - - - - - -  <<<
        Min ΔG for C149 vs C150 is -2.2 kcal/mol.
    - - - - - - - - - - - - - - - - - - - - - - - - - - - - - - - - - - - - - - - -
      5'> GTAGGATTTATAGAAGTGAGG >3'
                         ||| |
                     3'< CACCCACATCTTTCCTCTA <5'
   - - - - - - - - - - - - - - - - - - - - - - - - - - - - - - - - - - - - - - - -
        Min ΔG for C149 vs C149 is 3.88 kcal/mol.
    - - - - - - - - - - - - - - - - - - - - - - - - - - - - - - - - - - - - - - - -
      5'> GTAGGATTTATAGAAGTGAGG >3'
                          | |
                    3'< GGAGTGAAGATATTTAGGATG <5'
   - - - - - - - - - - - - - - - - - - - - - - - - - - - - - - - - - - - - - - - -
        Min ΔG for C150 vs C150 is 6.41 kcal/mol.
    - - - - - - - - - - - - - - - - - - - - - - - - - - - - - - - - - - - - - - - -
      5'> ATCTCCTTTCTACACCCAC >3'
                 |         |
            3'< CACCCACATCTTTCCTCTA <5'
   - - - - - - - - - - - - - - - - - - - - - - - - - - - - - - - - - - - - - - - -
>>> - - - - - - - - - - - - - - - - - - - - - - - - - - - - - - - - - - - - - - - -  <<<
```

```
****************************************************************************************
>>> - - - - - - - - - - - - - - - - - - - - - - - - - - - - - - - - - - - - - - - -  <<<
        Min ΔG for C151 vs C152 is -2.2 kcal/mol.
    - - - - - - - - - - - - - - - - - - - - - - - - - - - - - - - - - - - - - - - -
      5'> CCCTTTCCTCTCCCTCT >3'
                     || ||
                 3'< GGAAGTTTTATTTTTGTTTATTTTTTG <5'
   - - - - - - - - - - - - - - - - - - - - - - - - - - - - - - - - - - - - - - - -
    - - - - - - - - - - - - - - - - - - - - - - - - - - - - - - - - - - - - - - - -
       No dimers found for C151 vs C151
    - - - - - - - - - - - - - - - - - - - - - - - - - - - - - - - - - - - - - - - -
        Min ΔG for C152 vs C152 is 1.93 kcal/mol.
    - - - - - - - - - - - - - - - - - - - - - - - - - - - - - - - - - - - - - - - -
      5'> GTTTTTTATTTGTTTTTATTTTGAAGG >3'
                              || ||
                        3'< GGAAGTTTTATTTTTGTTTATTTTTTG <5'
   - - - - - - - - - - - - - - - - - - - - - - - - - - - - - - - - - - - - - - - -
>>> - - - - - - - - - - - - - - - - - - - - - - - - - - - - - - - - - - - - - - - -  <<<
****************************************************************************************
>>> - - - - - - - - - - - - - - - - - - - - - - - - - - - - - - - - - - - - - - - -  <<<
        Min ΔG for C153 vs C154 is -3.48 kcal/mol.
    - - - - - - - - - - - - - - - - - - - - - - - - - - - - - - - - - - - - - - - -
      5'> CAAATAAAAAACTTCCACCAAC >3'
                           || ||
                       3'< GGGTGAGGGGAGGGA <5'
   - - - - - - - - - - - - - - - - - - - - - - - - - - - - - - - - - - - - - - - -
        Min ΔG for C153 vs C153 is 5.5 kcal/mol.
    - - - - - - - - - - - - - - - - - - - - - - - - - - - - - - - - - - - - - - - -
      5'> CAAATAAAAAACTTCCACCAAC >3'
                      ||      ||
                 3'< CAACCACCTTCAAAAAATAAAC <5'
   - - - - - - - - - - - - - - - - - - - - - - - - - - - - - - - - - - - - - - - -
        Min ΔG for C154 vs C154 is 3.88 kcal/mol.
    - - - - - - - - - - - - - - - - - - - - - - - - - - - - - - - - - - - - - - - -
      5'> AGGGAGGGGAGTGGG >3'
                    | |
            3'< GGGTGAGGGGAGGGA <5'
   - - - - - - - - - - - - - - - - - - - - - - - - - - - - - - - - - - - - - - - -
>>> - - - - - - - - - - - - - - - - - - - - - - - - - - - - - - - - - - - - - - - -  <<<
****************************************************************************************
>>> - - - - - - - - - - - - - - - - - - - - - - - - - - - - - - - - - - - - - - - -  <<<
        Min ΔG for C155 vs C156 is -2.2 kcal/mol.
    - - - - - - - - - - - - - - - - - - - - - - - - - - - - - - - - - - - - - - - -
      5'> TTTGAGAAAAGGAAATAGAGGG >3'
                 ||    | |||||
            3'< ATTAAATCTATCTTTACAAACCTC <5'
   - - - - - - - - - - - - - - - - - - - - - - - - - - - - - - - - - - - - - - - -
        Min ΔG for C155 vs C155 is 4.44 kcal/mol.
    - - - - - - - - - - - - - - - - - - - - - - - - - - - - - - - - - - - - - - - -
      5'> TTTGAGAAAAGGAAATAGAGGG >3'
                         ||
                3'< GGGAGATAAAGGAAAAGAGTTT <5'
   - - - - - - - - - - - - - - - - - - - - - - - - - - - - - - - - - - - - - - - -
        Min ΔG for C156 vs C156 is -2.04 kcal/mol.
    - - - - - - - - - - - - - - - - - - - - - - - - - - - - - - - - - - - - - - - -
      5'> CTCCAAACATTTCTATCTAAATTA >3'
                           ||| |||
                       3'< ATTAAATCTATCTTTACAAACCTC <5'
   - - - - - - - - - - - - - - - - - - - - - - - - - - - - - - - - - - - - - - - -
>>> - - - - - - - - - - - - - - - - - - - - - - - - - - - - - - - - - - - - - - - -  <<<
****************************************************************************************
>>> - - - - - - - - - - - - - - - - - - - - - - - - - - - - - - - - - - - - - - - -  <<<
        Min ΔG for C157 vs C158 is -3.67 kcal/mol.
    - - - - - - - - - - - - - - - - - - - - - - - - - - - - - - - - - - - - - - - -
      5'> TTTTTTAATTTTGTAAGAAGGGATT >3'
                      | || |||||  |
                  3'< CCTTTTTCCCCCACAATCT <5'
   - - - - - - - - - - - - - - - - - - - - - - - - - - - - - - - - - - - - - - - -
        Min ΔG for C157 vs C157 is 0.02 kcal/mol.
    - - - - - - - - - - - - - - - - - - - - - - - - - - - - - - - - - - - - - - - -
      5'> TTTTTTAATTTTGTAAGAAGGGATT >3'
                           ||    ||
                       3'< TTAGGGAAGAATGTTTTAATTTTTT <5'
   - - - - - - - - - - - - - - - - - - - - - - - - - - - - - - - - - - - - - - - -
        Min ΔG for C158 vs C158 is 4.31 kcal/mol.
    - - - - - - - - - - - - - - - - - - - - - - - - - - - - - - - - - - - - - - - -
      5'> TCTAACACCCCCTTTTTCC >3'
             || |     | ||
      3'< CCTTTTTCCCCCACAATCT <5'
   - - - - - - - - - - - - - - - - - - - - - - - - - - - - - - - - - - - - - - - -
>>> - - - - - - - - - - - - - - - - - - - - - - - - - - - - - - - - - - - - - - - -  <<<
```

```
**************************************************************************************
>>> - - - - - - - - - - - - - - - - - - - - - - - - - - - - - - - - - - - - - - -  <<<
        Min ΔG for C159 vs C160 is -2.91 kcal/mol.
    - - - - - - - - - - - - - - - - - - - - - - - - - - - - - - - - - - - - - - -
      5'> AAATAATAAACAATTAAAATATCTTCTT >3'
                               ||   |||
                          3'< AGTGGAAGGTTGGATTAGG <5'
   - - - - - - - - - - - - - - - - - - - - - - - - - - - - - - - - - - - - - - -
        Min ΔG for C159 vs C159 is 0.71 kcal/mol.
    - - - - - - - - - - - - - - - - - - - - - - - - - - - - - - - - - - - - - - -
      5'> AAATAATAAACAATTAAAATATCTTCTT >3'
              || ||   |||  |||    || ||
          3'< TTCTTCTATAAAATTAACAAATAATAAA <5'
   - - - - - - - - - - - - - - - - - - - - - - - - - - - - - - - - - - - - - - -
        Min ΔG for C160 vs C160 is -0.13 kcal/mol.
    - - - - - - - - - - - - - - - - - - - - - - - - - - - - - - - - - - - - - - -
      5'> GGATTAGGTTGGAAGGTGA >3'
             | |  ||   ||  | |
         3'< AGTGGAAGGTTGGATTAGG <5'
   - - - - - - - - - - - - - - - - - - - - - - - - - - - - - - - - - - - - - - -
>>> - - - - - - - - - - - - - - - - - - - - - - - - - - - - - - - - - - - - - - -  <<<
**************************************************************************************
>>> - - - - - - - - - - - - - - - - - - - - - - - - - - - - - - - - - - - - - - -  <<<
        Min ΔG for C161 vs C162 is 0.78 kcal/mol.
    - - - - - - - - - - - - - - - - - - - - - - - - - - - - - - - - - - - - - - -
      5'> ACCCCCCAAAAACCTCAC >3'
                  ||||    |
            3'< AATTTTATTTTTTGGGGATTTGATT <5'
   - - - - - - - - - - - - - - - - - - - - - - - - - - - - - - - - - - - - - - -
        Min ΔG for C161 vs C161 is 6.26 kcal/mol.
    - - - - - - - - - - - - - - - - - - - - - - - - - - - - - - - - - - - - - - -
      5'> ACCCCCCAAAAACCTCAC >3'
                        | |
                   3'< CACTCCAAAAACCCCCCA <5'
   - - - - - - - - - - - - - - - - - - - - - - - - - - - - - - - - - - - - - - -
        Min ΔG for C162 vs C162 is -2.74 kcal/mol.
    - - - - - - - - - - - - - - - - - - - - - - - - - - - - - - - - - - - - - - -
      5'> TTAGTTTAGGGGTTTTTTATTTTAA >3'
                               ||||
                          3'< AATTTTATTTTTTGGGGATTTGATT <5'
   - - - - - - - - - - - - - - - - - - - - - - - - - - - - - - - - - - - - - - -
>>> - - - - - - - - - - - - - - - - - - - - - - - - - - - - - - - - - - - - - - -  <<<
**************************************************************************************
>>> - - - - - - - - - - - - - - - - - - - - - - - - - - - - - - - - - - - - - - -  <<<
        Min ΔG for C163 vs C164 is -1.31 kcal/mol.
    - - - - - - - - - - - - - - - - - - - - - - - - - - - - - - - - - - - - - - -
      5'> TTAAAAAACCACCACTTCCAAT >3'
                 ||      |  ||||
           3'< AGTGTATTAAATAGTTAATGTTGTT <5'
   - - - - - - - - - - - - - - - - - - - - - - - - - - - - - - - - - - - - - - -
        Min ΔG for C163 vs C163 is -1.39 kcal/mol.
    - - - - - - - - - - - - - - - - - - - - - - - - - - - - - - - - - - - - - - -
      5'> TTAAAAAACCACCACTTCCAAT >3'
                              ||
                         3'< TAACCTTCACCACCAAAAAATT <5'
   - - - - - - - - - - - - - - - - - - - - - - - - - - - - - - - - - - - - - - -
        Min ΔG for C164 vs C164 is 0.88 kcal/mol.
    - - - - - - - - - - - - - - - - - - - - - - - - - - - - - - - - - - - - - - -
      5'> TTGTTGTAATTGATAAATTATGTGA >3'
                                | |
                           3'< AGTGTATTAAATAGTTAATGTTGTT <5'
   - - - - - - - - - - - - - - - - - - - - - - - - - - - - - - - - - - - - - - -
>>> - - - - - - - - - - - - - - - - - - - - - - - - - - - - - - - - - - - - - - -  <<<
**************************************************************************************
>>> - - - - - - - - - - - - - - - - - - - - - - - - - - - - - - - - - - - - - - -  <<<
        Min ΔG for C165 vs C166 is -2.23 kcal/mol.
    - - - - - - - - - - - - - - - - - - - - - - - - - - - - - - - - - - - - - - -
      5'> CCTTAAAAAACAACTCTCAACC >3'
                       || |||
                   3'< GATAGTGAAATTTGTATTTTTAAATTA <5'
   - - - - - - - - - - - - - - - - - - - - - - - - - - - - - - - - - - - - - - -
        Min ΔG for C165 vs C165 is 5.82 kcal/mol.
    - - - - - - - - - - - - - - - - - - - - - - - - - - - - - - - - - - - - - - -
      5'> CCTTAAAAAACAACTCTCAACC >3'
           || | |      | | ||
      3'< CCAACTCTCAACAAAAAATTCC <5'
   - - - - - - - - - - - - - - - - - - - - - - - - - - - - - - - - - - - - - - -
        Min ΔG for C166 vs C166 is 0.94 kcal/mol.
    - - - - - - - - - - - - - - - - - - - - - - - - - - - - - - - - - - - - - - -
      5'> ATTAAATTTTTATGTTTAAAGTGATAG >3'
                                  ||
                            3'< GATAGTGAAATTTGTATTTTTAAATTA <5'
   - - - - - - - - - - - - - - - - - - - - - - - - - - - - - - - - - - - - - - -
>>> - - - - - - - - - - - - - - - - - - - - - - - - - - - - - - - - - - - - - - -  <<<
```

```
*****************************************************************************************
>>> - - - - - - - - - - - - - - - - - - - - - - - - - - - - - - - - - - - - - - - -  <<<
        Min ΔG for C167 vs C168 is -7.01 kcal/mol.
    - - - - - - - - - - - - - - - - - - - - - - - - - - - - - - - - - - - - - - - -
      5'> GTTTAGGTATAGGAGGGAG >3'
                |  | ||| ||||
           3'< ACCCAACCTTCCTCTCC <5'
   - - - - - - - - - - - - - - - - - - - - - - - - - - - - - - - - - - - - - - - -
        Min ΔG for C167 vs C167 is 4.68 kcal/mol.
    - - - - - - - - - - - - - - - - - - - - - - - - - - - - - - - - - - - - - - - -
      5'> GTTTAGGTATAGGAGGGAG >3'
            |   |     |   |
        3'< GAGGGAGGATATGGATTTG <5'
   - - - - - - - - - - - - - - - - - - - - - - - - - - - - - - - - - - - - - - - -
        Min ΔG for C168 vs C168 is 5.13 kcal/mol.
    - - - - - - - - - - - - - - - - - - - - - - - - - - - - - - - - - - - - - - - -
      5'> CCTCTCCTTCCAACCCA >3'
                |        |
             3'< ACCCAACCTTCCTCTCC <5'
   - - - - - - - - - - - - - - - - - - - - - - - - - - - - - - - - - - - - - - - -
>>> - - - - - - - - - - - - - - - - - - - - - - - - - - - - - - - - - - - - - - - -  <<<
*****************************************************************************************
>>> - - - - - - - - - - - - - - - - - - - - - - - - - - - - - - - - - - - - - - - -  <<<
        Min ΔG for C169 vs C170 is -4.09 kcal/mol.
    - - - - - - - - - - - - - - - - - - - - - - - - - - - - - - - - - - - - - - - -
      5'> TTTGGGTTGGAAGGAGAGG >3'
                      |||| ||
               3'< ACCCCTCACCCACATCA <5'
   - - - - - - - - - - - - - - - - - - - - - - - - - - - - - - - - - - - - - - - -
        Min ΔG for C169 vs C169 is 5.23 kcal/mol.
    - - - - - - - - - - - - - - - - - - - - - - - - - - - - - - - - - - - - - - - -
      5'> TTTGGGTTGGAAGGAGAGG >3'
              |        |
        3'< GGAGAGGAAGGTTGGGTTT <5'
   - - - - - - - - - - - - - - - - - - - - - - - - - - - - - - - - - - - - - - - -
        Min ΔG for C170 vs C170 is 4.72 kcal/mol.
    - - - - - - - - - - - - - - - - - - - - - - - - - - - - - - - - - - - - - - - -
      5'> ACTACACCCACTCCCCA >3'
                    |    |
                 3'< ACCCCTCACCCACATCA <5'
   - - - - - - - - - - - - - - - - - - - - - - - - - - - - - - - - - - - - - - - -
>>> - - - - - - - - - - - - - - - - - - - - - - - - - - - - - - - - - - - - - - - -  <<<
```

```
****************************************************************************************
>>> - - - - - - - - - - - - - - - - - - - - - - - - - - - - - - - - - - - - - - - -  <<<
        Min ΔG for C171 vs C172 is -3.07 kcal/mol.
    - - - - - - - - - - - - - - - - - - - - - - - - - - - - - - - - - - - - - - - -
      5'> GGTAAGTGTTTAGGATGGTT >3'
                      ||| || |
               3'< TTCCCTCCCCAAAACACTA <5'
   - - - - - - - - - - - - - - - - - - - - - - - - - - - - - - - - - - - - - - - -
        Min ΔG for C171 vs C171 is 0.31 kcal/mol.
    - - - - - - - - - - - - - - - - - - - - - - - - - - - - - - - - - - - - - - - -
      5'> GGTAAGTGTTTAGGATGGTT >3'
             ||   |      |    ||
         3'< TTGGTAGGATTTGTGAATGG <5'
   - - - - - - - - - - - - - - - - - - - - - - - - - - - - - - - - - - - - - - - -
        Min ΔG for C172 vs C172 is 2.87 kcal/mol.
    - - - - - - - - - - - - - - - - - - - - - - - - - - - - - - - - - - - - - - - -
      5'> ATCACAAAACCCCTCCCTT >3'
                ||         ||
            3'< TTCCCTCCCCAAAACACTA <5'
   - - - - - - - - - - - - - - - - - - - - - - - - - - - - - - - - - - - - - - - -
>>> - - - - - - - - - - - - - - - - - - - - - - - - - - - - - - - - - - - - - - - -  <<<
****************************************************************************************
>>> - - - - - - - - - - - - - - - - - - - - - - - - - - - - - - - - - - - - - - - -  <<<
        Min ΔG for C173 vs C174 is -1.95 kcal/mol.
    - - - - - - - - - - - - - - - - - - - - - - - - - - - - - - - - - - - - - - - -
      5'> AAAATAGGTAGTTTTTTGTGATGA >3'
                || |||      | |
            3'< CCCTCATCCTCCCCAC <5'
   - - - - - - - - - - - - - - - - - - - - - - - - - - - - - - - - - - - - - - - -
        Min ΔG for C173 vs C173 is 0.38 kcal/mol.
    - - - - - - - - - - - - - - - - - - - - - - - - - - - - - - - - - - - - - - - -
      5'> AAAATAGGTAGTTTTTTGTGATGA >3'
                               | |
                           3'< AGTAGTGTTTTTTGATGGATAAAA <5'
   - - - - - - - - - - - - - - - - - - - - - - - - - - - - - - - - - - - - - - - -
        Min ΔG for C174 vs C174 is 5.75 kcal/mol.
    - - - - - - - - - - - - - - - - - - - - - - - - - - - - - - - - - - - - - - - -
      5'> CACCCCTCCTACTCCC >3'
                   ||
          3'< CCCTCATCCTCCCCAC <5'
   - - - - - - - - - - - - - - - - - - - - - - - - - - - - - - - - - - - - - - - -
>>> - - - - - - - - - - - - - - - - - - - - - - - - - - - - - - - - - - - - - - - -  <<<
****************************************************************************************
>>> - - - - - - - - - - - - - - - - - - - - - - - - - - - - - - - - - - - - - - - -  <<<
        Min ΔG for C175 vs C176 is -2.84 kcal/mol.
    - - - - - - - - - - - - - - - - - - - - - - - - - - - - - - - - - - - - - - - -
      5'> TTCACTCCTCCCCCATC >3'
                      ||||
                  3'< GGTAAAAATTTTTTTTTTTTTTTTTTTAG <5'
   - - - - - - - - - - - - - - - - - - - - - - - - - - - - - - - - - - - - - - - -
        Min ΔG for C175 vs C175 is 2.72 kcal/mol.
    - - - - - - - - - - - - - - - - - - - - - - - - - - - - - - - - - - - - - - - -
      5'> TTCACTCCTCCCCCATC >3'
                        ||
                   3'< CTACCCCCTCCTCACTT <5'
   - - - - - - - - - - - - - - - - - - - - - - - - - - - - - - - - - - - - - - - -
        Min ΔG for C176 vs C176 is -0.13 kcal/mol.
    - - - - - - - - - - - - - - - - - - - - - - - - - - - - - - - - - - - - - - - -
      5'> GATTTTTTTTTTTTTTTTTTTAAAAATGG >3'
             |||||            |||||
      3'< GGTAAAAATTTTTTTTTTTTTTTTTTTAG <5'
   - - - - - - - - - - - - - - - - - - - - - - - - - - - - - - - - - - - - - - - -
>>> - - - - - - - - - - - - - - - - - - - - - - - - - - - - - - - - - - - - - - - -  <<<
****************************************************************************************
>>> - - - - - - - - - - - - - - - - - - - - - - - - - - - - - - - - - - - - - - - -  <<<
        Min ΔG for C177 vs C178 is -3.65 kcal/mol.
    - - - - - - - - - - - - - - - - - - - - - - - - - - - - - - - - - - - - - - - -
      5'> AGGGTTTGGTATGTAGAGAG >3'
            | ||||| | |  |
       3'< ACTAAACCTTCCCACCAATT <5'
   - - - - - - - - - - - - - - - - - - - - - - - - - - - - - - - - - - - - - - - -
        Min ΔG for C177 vs C177 is 1.52 kcal/mol.
    - - - - - - - - - - - - - - - - - - - - - - - - - - - - - - - - - - - - - - - -
      5'> AGGGTTTGGTATGTAGAGAG >3'
                   | | || | |
              3'< GAGAGATGTATGGTTTGGGA <5'
   - - - - - - - - - - - - - - - - - - - - - - - - - - - - - - - - - - - - - - - -
        Min ΔG for C178 vs C178 is 1.6 kcal/mol.
    - - - - - - - - - - - - - - - - - - - - - - - - - - - - - - - - - - - - - - - -
      5'> TTAACCACCCTTCCAAATCA >3'
                               | |
                           3'< ACTAAACCTTCCCACCAATT <5'
   - - - - - - - - - - - - - - - - - - - - - - - - - - - - - - - - - - - - - - - -
>>> - - - - - - - - - - - - - - - - - - - - - - - - - - - - - - - - - - - - - - - -  <<<
```

```
*****************************************************************************************
>>> - - - - - - - - - - - - - - - - - - - - - - - - - - - - - - - - - - - - - - - -  <<<
        Min ΔG for C179 vs C180 is -2.96 kcal/mol.
    - - - - - - - - - - - - - - - - - - - - - - - - - - - - - - - - - - - - - - - -
      5'> TATATATATATATATATAGTTGTTTGTT >3'
                     |    ||   |||| ||
               3'< ACTCCTCTTCCTCAAAAAAC <5'
   - - - - - - - - - - - - - - - - - - - - - - - - - - - - - - - - - - - - - - - -
        Min ΔG for C179 vs C179 is 3.92 kcal/mol.
    - - - - - - - - - - - - - - - - - - - - - - - - - - - - - - - - - - - - - - - -
      5'> TATATATATATATATATAGTTGTTTGTT >3'
                       | | |     | | |
                  3'< TTGTTTGTTGATATATATATATATATAT <5'
   - - - - - - - - - - - - - - - - - - - - - - - - - - - - - - - - - - - - - - - -
        Min ΔG for C180 vs C180 is 2.08 kcal/mol.
    - - - - - - - - - - - - - - - - - - - - - - - - - - - - - - - - - - - - - - - -
      5'> CAAAAAACTCCTTCTCCTCA >3'
                           | |
                       3'< ACTCCTCTTCCTCAAAAAAC <5'
   - - - - - - - - - - - - - - - - - - - - - - - - - - - - - - - - - - - - - - - -
>>> - - - - - - - - - - - - - - - - - - - - - - - - - - - - - - - - - - - - - - - -  <<<
*****************************************************************************************
>>> - - - - - - - - - - - - - - - - - - - - - - - - - - - - - - - - - - - - - - - -  <<<
        Min ΔG for C181 vs C182 is -4.9 kcal/mol.
    - - - - - - - - - - - - - - - - - - - - - - - - - - - - - - - - - - - - - - - -
      5'> GGGAGAGGGTTGTGGG >3'
           ||||  ||  | | |
       3'< CCTCCTCCTCCCCACT <5'
   - - - - - - - - - - - - - - - - - - - - - - - - - - - - - - - - - - - - - - - -
        Min ΔG for C181 vs C181 is 4.72 kcal/mol.
    - - - - - - - - - - - - - - - - - - - - - - - - - - - - - - - - - - - - - - - -
      5'> GGGAGAGGGTTGTGGG >3'
             | |    | |
      3'< GGGTGTTGGGAGAGGG <5'
   - - - - - - - - - - - - - - - - - - - - - - - - - - - - - - - - - - - - - - - -
        Min ΔG for C182 vs C182 is 7.4 kcal/mol.
    - - - - - - - - - - - - - - - - - - - - - - - - - - - - - - - - - - - - - - - -
      5'> TCACCCCTCCTCCTCC >3'
            |          |
      3'< CCTCCTCCTCCCCACT <5'
   - - - - - - - - - - - - - - - - - - - - - - - - - - - - - - - - - - - - - - - -
>>> - - - - - - - - - - - - - - - - - - - - - - - - - - - - - - - - - - - - - - - -  <<<
*****************************************************************************************
>>> - - - - - - - - - - - - - - - - - - - - - - - - - - - - - - - - - - - - - - - -  <<<
        Min ΔG for C183 vs C184 is -2.44 kcal/mol.
    - - - - - - - - - - - - - - - - - - - - - - - - - - - - - - - - - - - - - - - -
      5'> TTTTAGTTTATGGTGAGTGTGG >3'
                                ||
                          3'< CCTCAAAATCCATTCAAATCC <5'
   - - - - - - - - - - - - - - - - - - - - - - - - - - - - - - - - - - - - - - - -
        Min ΔG for C183 vs C183 is 4.31 kcal/mol.
    - - - - - - - - - - - - - - - - - - - - - - - - - - - - - - - - - - - - - - - -
      5'> TTTTAGTTTATGGTGAGTGTGG >3'
                | |        | |
      3'< GGTGTGAGTGGTATTTGATTTT <5'
   - - - - - - - - - - - - - - - - - - - - - - - - - - - - - - - - - - - - - - - -
        Min ΔG for C184 vs C184 is 5.33 kcal/mol.
    - - - - - - - - - - - - - - - - - - - - - - - - - - - - - - - - - - - - - - - -
      5'> CCTAAACTTACCTAAAACTCC >3'
               |  ||       ||  |
        3'< CCTCAAAATCCATTCAAATCC <5'
   - - - - - - - - - - - - - - - - - - - - - - - - - - - - - - - - - - - - - - - -
>>> - - - - - - - - - - - - - - - - - - - - - - - - - - - - - - - - - - - - - - - -  <<<
*****************************************************************************************
>>> - - - - - - - - - - - - - - - - - - - - - - - - - - - - - - - - - - - - - - - -  <<<
        Min ΔG for C185 vs C186 is -2.79 kcal/mol.
    - - - - - - - - - - - - - - - - - - - - - - - - - - - - - - - - - - - - - - - -
      5'> GAGTGTGGTTGGGGGTG >3'
                     || ||
                 3'< CCTCAAAATCCATTCAAATCC <5'
   - - - - - - - - - - - - - - - - - - - - - - - - - - - - - - - - - - - - - - - -
        Min ΔG for C185 vs C185 is 5.33 kcal/mol.
    - - - - - - - - - - - - - - - - - - - - - - - - - - - - - - - - - - - - - - - -
      5'> GAGTGTGGTTGGGGGTG >3'
           |             |
      3'< GTGGGGGTTGGTGTGAG <5'
   - - - - - - - - - - - - - - - - - - - - - - - - - - - - - - - - - - - - - - - -
        Min ΔG for C186 vs C186 is 5.33 kcal/mol.
    - - - - - - - - - - - - - - - - - - - - - - - - - - - - - - - - - - - - - - - -
      5'> CCTAAACTTACCTAAAACTCC >3'
                |  ||      ||  |
        3'< CCTCAAAATCCATTCAAATCC <5'
   - - - - - - - - - - - - - - - - - - - - - - - - - - - - - - - - - - - - - - - -
>>> - - - - - - - - - - - - - - - - - - - - - - - - - - - - - - - - - - - - - - - -  <<<
```

```
*************************************************************************************
>>> - - - - - - - - - - - - - - - - - - - - - - - - - - - - - - - - - - - - - -  <<<
        Min ΔG for C187 vs C188 is -4.89 kcal/mol.
    - - - - - - - - - - - - - - - - - - - - - - - - - - - - - - - - - - - - - - -
      5'> TTTTTTTTTTTTTTATAGGTTATAGGTT >3'
                                  ||||
                              3'< CCAAATCCCCCAAATCTTAC <5'
   - - - - - - - - - - - - - - - - - - - - - - - - - - - - - - - - - - - - - - -
        Min ΔG for C187 vs C187 is 0.39 kcal/mol.
    - - - - - - - - - - - - - - - - - - - - - - - - - - - - - - - - - - - - - - -
      5'> TTTTTTTTTTTTTTATAGGTTATAGGTT >3'
                          |   ||||   |
                      3'< TTGGATATTGGATATTTTTTTTTTTTTT <5'
   - - - - - - - - - - - - - - - - - - - - - - - - - - - - - - - - - - - - - - -
        Min ΔG for C188 vs C188 is 5.22 kcal/mol.
    - - - - - - - - - - - - - - - - - - - - - - - - - - - - - - - - - - - - - - -
      5'> CATTCTAAACCCCCTAAACC >3'
            ||              ||
      3'< CCAAATCCCCCAAATCTTAC <5'
   - - - - - - - - - - - - - - - - - - - - - - - - - - - - - - - - - - - - - - -
>>> - - - - - - - - - - - - - - - - - - - - - - - - - - - - - - - - - - - - - -  <<<
*************************************************************************************
>>> - - - - - - - - - - - - - - - - - - - - - - - - - - - - - - - - - - - - - -  <<<
        Min ΔG for C189 vs C190 is -1.91 kcal/mol.
    - - - - - - - - - - - - - - - - - - - - - - - - - - - - - - - - - - - - - - -
      5'> TAAATGTGTATATGGAGGGAG >3'
                              ||
                          3'< TCTAAAATAACTAAACCTTAAACTA <5'
   - - - - - - - - - - - - - - - - - - - - - - - - - - - - - - - - - - - - - - -
        Min ΔG for C189 vs C189 is 4.01 kcal/mol.
    - - - - - - - - - - - - - - - - - - - - - - - - - - - - - - - - - - - - - - -
      5'> TAAATGTGTATATGGAGGGAG >3'
                |   |  ||  |   |
         3'< GAGGGAGGTATATGTGTAAAT <5'
   - - - - - - - - - - - - - - - - - - - - - - - - - - - - - - - - - - - - - - -
        Min ΔG for C190 vs C190 is 1.83 kcal/mol.
    - - - - - - - - - - - - - - - - - - - - - - - - - - - - - - - - - - - - - - -
      5'> ATCAAATTCCAAATCAATAAAATCT >3'
                                |  |
                            3'< TCTAAAATAACTAAACCTTAAACTA <5'
   - - - - - - - - - - - - - - - - - - - - - - - - - - - - - - - - - - - - - - -
>>> - - - - - - - - - - - - - - - - - - - - - - - - - - - - - - - - - - - - - -  <<<
*************************************************************************************
>>> - - - - - - - - - - - - - - - - - - - - - - - - - - - - - - - - - - - - - -  <<<
        Min ΔG for C191 vs C192 is -2.15 kcal/mol.
    - - - - - - - - - - - - - - - - - - - - - - - - - - - - - - - - - - - - - - -
      5'> AAAAACCTAAACTTCATCCCTC >3'
                             |||
                         3'< GGATGGAATTGATTTTGTTTTTTA <5'
   - - - - - - - - - - - - - - - - - - - - - - - - - - - - - - - - - - - - - - -
        Min ΔG for C191 vs C191 is 5.19 kcal/mol.
    - - - - - - - - - - - - - - - - - - - - - - - - - - - - - - - - - - - - - - -
      5'> AAAAACCTAAACTTCATCCCTC >3'
                          ||
                 3'< CTCCCTACTTCAAATCCAAAAA <5'
   - - - - - - - - - - - - - - - - - - - - - - - - - - - - - - - - - - - - - - -
        Min ΔG for C192 vs C192 is 2.4 kcal/mol.
    - - - - - - - - - - - - - - - - - - - - - - - - - - - - - - - - - - - - - - -
      5'> ATTTTTTGTTTTAGTTAAGGTAGG >3'
                               ||
                         3'< GGATGGAATTGATTTTGTTTTTTA <5'
   - - - - - - - - - - - - - - - - - - - - - - - - - - - - - - - - - - - - - - -
>>> - - - - - - - - - - - - - - - - - - - - - - - - - - - - - - - - - - - - - -  <<<
*************************************************************************************
>>> - - - - - - - - - - - - - - - - - - - - - - - - - - - - - - - - - - - - - -  <<<
        Min ΔG for C193 vs C194 is -3.43 kcal/mol.
    - - - - - - - - - - - - - - - - - - - - - - - - - - - - - - - - - - - - - - -
      5'> TTTTTGGGGTTGGTAGTAAATT >3'
                             ||||
                         3'< TTAACAAACCAAAAACCACATAAA <5'
   - - - - - - - - - - - - - - - - - - - - - - - - - - - - - - - - - - - - - - -
        Min ΔG for C193 vs C193 is -3.43 kcal/mol.
    - - - - - - - - - - - - - - - - - - - - - - - - - - - - - - - - - - - - - - -
      5'> TTTTTGGGGTTGGTAGTAAATT >3'
                             ||||
                         3'< TTAAATGATGGTTGGGGTTTTT <5'
   - - - - - - - - - - - - - - - - - - - - - - - - - - - - - - - - - - - - - - -
        Min ΔG for C194 vs C194 is -3.34 kcal/mol.
    - - - - - - - - - - - - - - - - - - - - - - - - - - - - - - - - - - - - - - -
      5'> AAATACACCAAAAACCAAACAATT >3'
                               ||||
                           3'< TTAACAAACCAAAAACCACATAAA <5'
   - - - - - - - - - - - - - - - - - - - - - - - - - - - - - - - - - - - - - - -
>>> - - - - - - - - - - - - - - - - - - - - - - - - - - - - - - - - - - - - - -  <<<
```

```
************************************************************************************************
>>> - - - - - - - - - - - - - - - - - - - - - - - - - - - - - - - - - - - - - - - - - - - -  <<<
        Min ΔG for C195 vs C196 is -3.48 kcal/mol.
    - - - - - - - - - - - - - - - - - - - - - - - - - - - - - - - - - - - - - - - - - - - -
      5'> AGATTTATAGTTAATATGTTAGGTG >3'
                                |||
                            3'< CACCCTTCACCAACACC <5'
   - - - - - - - - - - - - - - - - - - - - - - - - - - - - - - - - - - - - - - - - - - - -
        Min ΔG for C195 vs C195 is 1.77 kcal/mol.
    - - - - - - - - - - - - - - - - - - - - - - - - - - - - - - - - - - - - - - - - - - - -
      5'> AGATTTATAGTTAATATGTTAGGTG >3'
                  |  ||| || |||  |
             3'< GTGGATTGTATAATTGATATTTAGA <5'
   - - - - - - - - - - - - - - - - - - - - - - - - - - - - - - - - - - - - - - - - - - - -
        Min ΔG for C196 vs C196 is 6.64 kcal/mol.
    - - - - - - - - - - - - - - - - - - - - - - - - - - - - - - - - - - - - - - - - - - - -
      5'> CCACAACCACTTCCCAC >3'
                     |   |
                3'< CACCCTTCACCAACACC <5'
   - - - - - - - - - - - - - - - - - - - - - - - - - - - - - - - - - - - - - - - - - - - -
>>> - - - - - - - - - - - - - - - - - - - - - - - - - - - - - - - - - - - - - - - - - - - -  <<<
************************************************************************************************
>>> - - - - - - - - - - - - - - - - - - - - - - - - - - - - - - - - - - - - - - - - - - - -  <<<
        Min ΔG for C197 vs C198 is -2.46 kcal/mol.
    - - - - - - - - - - - - - - - - - - - - - - - - - - - - - - - - - - - - - - - - - - - -
      5'> TTTTAGTTTTTTTTAGTTATAAGATGG >3'
            |  |||||  || |   |
        3'< ACACAAAACCAAACCTCAAAAA <5'
   - - - - - - - - - - - - - - - - - - - - - - - - - - - - - - - - - - - - - - - - - - - -
        Min ΔG for C197 vs C197 is 3.26 kcal/mol.
    - - - - - - - - - - - - - - - - - - - - - - - - - - - - - - - - - - - - - - - - - - - -
      5'> TTTTAGTTTTTTTTAGTTATAAGATGG >3'
                                 ||
                           3'< GGTAGAATATTGATTTTTTTTGATTTT <5'
   - - - - - - - - - - - - - - - - - - - - - - - - - - - - - - - - - - - - - - - - - - - -
        Min ΔG for C198 vs C198 is 5.43 kcal/mol.
    - - - - - - - - - - - - - - - - - - - - - - - - - - - - - - - - - - - - - - - - - - - -
      5'> AAAAACTCCAAACCAAAACACA >3'
                |              |
            3'< ACACAAAACCAAACCTCAAAAA <5'
   - - - - - - - - - - - - - - - - - - - - - - - - - - - - - - - - - - - - - - - - - - - -
>>> - - - - - - - - - - - - - - - - - - - - - - - - - - - - - - - - - - - - - - - - - - - -  <<<
************************************************************************************************
>>> - - - - - - - - - - - - - - - - - - - - - - - - - - - - - - - - - - - - - - - - - - - -  <<<
        Min ΔG for C199 vs C200 is -3.49 kcal/mol.
    - - - - - - - - - - - - - - - - - - - - - - - - - - - - - - - - - - - - - - - - - - - -
      5'> TAATCACATCTTCTATCACTCA >3'
                          || |||
                    3'< AGGTTAGTTTGTGTGTTGATTT <5'
   - - - - - - - - - - - - - - - - - - - - - - - - - - - - - - - - - - - - - - - - - - - -
        Min ΔG for C199 vs C199 is 2.08 kcal/mol.
    - - - - - - - - - - - - - - - - - - - - - - - - - - - - - - - - - - - - - - - - - - - -
      5'> TAATCACATCTTCTATCACTCA >3'
                             | |
                         3'< ACTCACTATCTTCTACACTAAT <5'
   - - - - - - - - - - - - - - - - - - - - - - - - - - - - - - - - - - - - - - - - - - - -
        Min ΔG for C200 vs C200 is 1.66 kcal/mol.
    - - - - - - - - - - - - - - - - - - - - - - - - - - - - - - - - - - - - - - - - - - - -
      5'> TTTAGTTGTGTGTTTGATTGGA >3'
                            |  |
                        3'< AGGTTAGTTTGTGTGTTGATTT <5'
   - - - - - - - - - - - - - - - - - - - - - - - - - - - - - - - - - - - - - - - - - - - -
>>> - - - - - - - - - - - - - - - - - - - - - - - - - - - - - - - - - - - - - - - - - - - -  <<<
************************************************************************************************
>>> - - - - - - - - - - - - - - - - - - - - - - - - - - - - - - - - - - - - - - - - - - - -  <<<
        Min ΔG for C201 vs C202 is 0.36 kcal/mol.
    - - - - - - - - - - - - - - - - - - - - - - - - - - - - - - - - - - - - - - - - - - - -
      5'> AAACTATCCAATCAAACACACA >3'
                            |||
                     3'< GAAGTGATGAGATATAGGAG <5'
   - - - - - - - - - - - - - - - - - - - - - - - - - - - - - - - - - - - - - - - - - - - -
        Min ΔG for C201 vs C201 is 4.95 kcal/mol.
    - - - - - - - - - - - - - - - - - - - - - - - - - - - - - - - - - - - - - - - - - - - -
      5'> AAACTATCCAATCAAACACACA >3'
                |              |
            3'< ACACACAAACTAACCTATCAAA <5'
   - - - - - - - - - - - - - - - - - - - - - - - - - - - - - - - - - - - - - - - - - - - -
        Min ΔG for C202 vs C202 is 2.67 kcal/mol.
    - - - - - - - - - - - - - - - - - - - - - - - - - - - - - - - - - - - - - - - - - - - -
      5'> GAGGATATAGAGTAGTGAAG >3'
                 |  | || |  |
            3'< GAAGTGATGAGATATAGGAG <5'
   - - - - - - - - - - - - - - - - - - - - - - - - - - - - - - - - - - - - - - - - - - - -
>>> - - - - - - - - - - - - - - - - - - - - - - - - - - - - - - - - - - - - - - - - - - - -  <<<
```

```
****************************************************************************************
>>> - - - - - - - - - - - - - - - - - - - - - - - - - - - - - - - - - - - - - - - -  <<<
        Min ΔG for C203 vs C204 is -3.48 kcal/mol.
    - - - - - - - - - - - - - - - - - - - - - - - - - - - - - - - - - - - - - - - -
      5'> GGGTTTTTTGGGAATTGTAG >3'
                     | |  ||||
               3'< TTCCTCCCATCTCTAACAAA <5'
   - - - - - - - - - - - - - - - - - - - - - - - - - - - - - - - - - - - - - - - -
        Min ΔG for C203 vs C203 is 0.64 kcal/mol.
    - - - - - - - - - - - - - - - - - - - - - - - - - - - - - - - - - - - - - - - -
      5'> GGGTTTTTTGGGAATTGTAG >3'
                           ||
                      3'< GATGTTAAGGGTTTTTTGGG <5'
   - - - - - - - - - - - - - - - - - - - - - - - - - - - - - - - - - - - - - - - -
        Min ΔG for C204 vs C204 is 2.81 kcal/mol.
    - - - - - - - - - - - - - - - - - - - - - - - - - - - - - - - - - - - - - - - -
      5'> AAACAATCTCTACCCTCCTT >3'
              ||            ||
          3'< TTCCTCCCATCTCTAACAAA <5'
   - - - - - - - - - - - - - - - - - - - - - - - - - - - - - - - - - - - - - - - -
>>> - - - - - - - - - - - - - - - - - - - - - - - - - - - - - - - - - - - - - - - -  <<<
****************************************************************************************
>>> - - - - - - - - - - - - - - - - - - - - - - - - - - - - - - - - - - - - - - - -  <<<
        Min ΔG for C205 vs C206 is -2.38 kcal/mol.
    - - - - - - - - - - - - - - - - - - - - - - - - - - - - - - - - - - - - - - - -
      5'> TCTATCCCCTCTATCCCTC >3'
                        || ||
                    3'< GGTAGTATTGGTTGGATAGT <5'
   - - - - - - - - - - - - - - - - - - - - - - - - - - - - - - - - - - - - - - - -
        Min ΔG for C205 vs C205 is 5.11 kcal/mol.
    - - - - - - - - - - - - - - - - - - - - - - - - - - - - - - - - - - - - - - - -
      5'> TCTATCCCCTCTATCCCTC >3'
                      ||
             3'< CTCCCTATCTCCCCTATCT <5'
   - - - - - - - - - - - - - - - - - - - - - - - - - - - - - - - - - - - - - - - -
        Min ΔG for C206 vs C206 is 1.93 kcal/mol.
    - - - - - - - - - - - - - - - - - - - - - - - - - - - - - - - - - - - - - - - -
      5'> TGATAGGTTGGTTATGATGG >3'
                       || ||
                 3'< GGTAGTATTGGTTGGATAGT <5'
   - - - - - - - - - - - - - - - - - - - - - - - - - - - - - - - - - - - - - - - -
>>> - - - - - - - - - - - - - - - - - - - - - - - - - - - - - - - - - - - - - - - -  <<<
****************************************************************************************
>>> - - - - - - - - - - - - - - - - - - - - - - - - - - - - - - - - - - - - - - - -  <<<
        Min ΔG for C207 vs C208 is -1.38 kcal/mol.
    - - - - - - - - - - - - - - - - - - - - - - - - - - - - - - - - - - - - - - - -
      5'> TTTTGAGTTTTTATTGGTTTAGTAG >3'
                           ||| |||
                 3'< CCCTAAAAAACATAATTAAAAAACC <5'
   - - - - - - - - - - - - - - - - - - - - - - - - - - - - - - - - - - - - - - - -
        Min ΔG for C207 vs C207 is 0.64 kcal/mol.
    - - - - - - - - - - - - - - - - - - - - - - - - - - - - - - - - - - - - - - - -
      5'> TTTTGAGTTTTTATTGGTTTAGTAG >3'
                                ||
                           3'< GATGATTTGGTTATTTTTGAGTTTT <5'
   - - - - - - - - - - - - - - - - - - - - - - - - - - - - - - - - - - - - - - - -
        Min ΔG for C208 vs C208 is 3.38 kcal/mol.
    - - - - - - - - - - - - - - - - - - - - - - - - - - - - - - - - - - - - - - - -
      5'> CCAAAAAATTAATACAAAAAATCCC >3'
                 |||  |   |  |||
          3'< CCCTAAAAAACATAATTAAAAAACC <5'
   - - - - - - - - - - - - - - - - - - - - - - - - - - - - - - - - - - - - - - - -
>>> - - - - - - - - - - - - - - - - - - - - - - - - - - - - - - - - - - - - - - - -  <<<
****************************************************************************************
>>> - - - - - - - - - - - - - - - - - - - - - - - - - - - - - - - - - - - - - - - -  <<<
        Min ΔG for C209 vs C210 is -4.12 kcal/mol.
    - - - - - - - - - - - - - - - - - - - - - - - - - - - - - - - - - - - - - - - -
      5'> ATCCCCACCCCAAACTAAT >3'
           ||   ||||   || ||||
      3'< AAGTTTTGGGAGTTAATTAGAG <5'
   - - - - - - - - - - - - - - - - - - - - - - - - - - - - - - - - - - - - - - - -
        Min ΔG for C209 vs C209 is -1.39 kcal/mol.
    - - - - - - - - - - - - - - - - - - - - - - - - - - - - - - - - - - - - - - - -
      5'> ATCCCCACCCCAAACTAAT >3'
                           ||
                       3'< TAATCAAACCCCACCCCTA <5'
   - - - - - - - - - - - - - - - - - - - - - - - - - - - - - - - - - - - - - - - -
        Min ΔG for C210 vs C210 is -1.26 kcal/mol.
    - - - - - - - - - - - - - - - - - - - - - - - - - - - - - - - - - - - - - - - -
      5'> GAGATTAATTGAGGGTTTTGAA >3'
                           || ||
                       3'< AAGTTTTGGGAGTTAATTAGAG <5'
   - - - - - - - - - - - - - - - - - - - - - - - - - - - - - - - - - - - - - - - -
>>> - - - - - - - - - - - - - - - - - - - - - - - - - - - - - - - - - - - - - - - -  <<<
```

```
*****************************************************************************************
>>> - - - - - - - - - - - - - - - - - - - - - - - - - - - - - - - - - - - - - - - -  <<<
        Min ΔG for C211 vs C212 is -2.34 kcal/mol.
    - - - - - - - - - - - - - - - - - - - - - - - - - - - - - - - - - - - - - - - -
      5'> ATAAAAACCCTATCTCCCAAAT >3'
                          ||| |
                      3'< GGTGTGGGTGGGTGTG <5'
   - - - - - - - - - - - - - - - - - - - - - - - - - - - - - - - - - - - - - - - -
        Min ΔG for C211 vs C211 is -1.39 kcal/mol.
    - - - - - - - - - - - - - - - - - - - - - - - - - - - - - - - - - - - - - - - -
      5'> ATAAAAACCCTATCTCCCAAAT >3'
                              ||
                          3'< TAAACCCTCTATCCCAAAAATA <5'
   - - - - - - - - - - - - - - - - - - - - - - - - - - - - - - - - - - - - - - - -
    - - - - - - - - - - - - - - - - - - - - - - - - - - - - - - - - - - - - - - - -
       No dimers found for C212 vs C212
    - - - - - - - - - - - - - - - - - - - - - - - - - - - - - - - - - - - - - - - -
>>> - - - - - - - - - - - - - - - - - - - - - - - - - - - - - - - - - - - - - - - -  <<<
*****************************************************************************************
>>> - - - - - - - - - - - - - - - - - - - - - - - - - - - - - - - - - - - - - - - -  <<<
        Min ΔG for C213 vs C214 is -1.68 kcal/mol.
    - - - - - - - - - - - - - - - - - - - - - - - - - - - - - - - - - - - - - - - -
      5'> AAAAACAACCTAACAAAAACAAAAT >3'
                 |||   |    | |
             3'< TGGGGAGAGGATTTGGG <5'
   - - - - - - - - - - - - - - - - - - - - - - - - - - - - - - - - - - - - - - - -
        Min ΔG for C213 vs C213 is -1.39 kcal/mol.
    - - - - - - - - - - - - - - - - - - - - - - - - - - - - - - - - - - - - - - - -
      5'> AAAAACAACCTAACAAAAACAAAAT >3'
                                 ||
                             3'< TAAAACAAAAACAATCCAACAAAAA <5'
   - - - - - - - - - - - - - - - - - - - - - - - - - - - - - - - - - - - - - - - -
        Min ΔG for C214 vs C214 is 1.76 kcal/mol.
    - - - - - - - - - - - - - - - - - - - - - - - - - - - - - - - - - - - - - - - -
      5'> GGGTTTAGGAGAGGGGT >3'
                     |    |
                 3'< TGGGGAGAGGATTTGGG <5'
   - - - - - - - - - - - - - - - - - - - - - - - - - - - - - - - - - - - - - - - -
>>> - - - - - - - - - - - - - - - - - - - - - - - - - - - - - - - - - - - - - - - -  <<<
*****************************************************************************************
>>> - - - - - - - - - - - - - - - - - - - - - - - - - - - - - - - - - - - - - - - -  <<<
        Min ΔG for C215 vs C216 is -5.99 kcal/mol.
    - - - - - - - - - - - - - - - - - - - - - - - - - - - - - - - - - - - - - - - -
      5'> TATAGGTTTGTGGGTAGAGT >3'
             | ||  | ||||  |||
         3'< TTCATCCTCCCAATTCAACA <5'
   - - - - - - - - - - - - - - - - - - - - - - - - - - - - - - - - - - - - - - - -
        Min ΔG for C215 vs C215 is -0.88 kcal/mol.
    - - - - - - - - - - - - - - - - - - - - - - - - - - - - - - - - - - - - - - - -
      5'> TATAGGTTTGTGGGTAGAGT >3'
                           | |
                       3'< TGAGATGGGTGTTTGGATAT <5'
   - - - - - - - - - - - - - - - - - - - - - - - - - - - - - - - - - - - - - - - -
        Min ΔG for C216 vs C216 is 1.45 kcal/mol.
    - - - - - - - - - - - - - - - - - - - - - - - - - - - - - - - - - - - - - - - -
      5'> ACAACTTAACCCTCCTACTT >3'
                          |  |
                      3'< TTCATCCTCCCAATTCAACA <5'
   - - - - - - - - - - - - - - - - - - - - - - - - - - - - - - - - - - - - - - - -
>>> - - - - - - - - - - - - - - - - - - - - - - - - - - - - - - - - - - - - - - - -  <<<
*****************************************************************************************
>>> - - - - - - - - - - - - - - - - - - - - - - - - - - - - - - - - - - - - - - - -  <<<
        Min ΔG for C217 vs C218 is -0.77 kcal/mol.
    - - - - - - - - - - - - - - - - - - - - - - - - - - - - - - - - - - - - - - - -
      5'> TTTCTAAAATAACCATATCATAACT >3'
                |  |    || |  |||
            3'< TAAAGATAATGGAGATGAATGAT <5'
   - - - - - - - - - - - - - - - - - - - - - - - - - - - - - - - - - - - - - - - -
        Min ΔG for C217 vs C217 is 1.93 kcal/mol.
    - - - - - - - - - - - - - - - - - - - - - - - - - - - - - - - - - - - - - - - -
      5'> TTTCTAAAATAACCATATCATAACT >3'
                               |  |
                           3'< TCAATACTATACCAATAAAATCTTT <5'
   - - - - - - - - - - - - - - - - - - - - - - - - - - - - - - - - - - - - - - - -
        Min ΔG for C218 vs C218 is -1.39 kcal/mol.
    - - - - - - - - - - - - - - - - - - - - - - - - - - - - - - - - - - - - - - - -
      5'> TAGTAAGTAGAGGTAATAGAAAAT >3'
                                ||
                            3'< TAAAAGATAATGGAGATGAATGAT <5'
   - - - - - - - - - - - - - - - - - - - - - - - - - - - - - - - - - - - - - - - -
>>> - - - - - - - - - - - - - - - - - - - - - - - - - - - - - - - - - - - - - - - -  <<<
```

```
****************************************************************************************
>>> - - - - - - - - - - - - - - - - - - - - - - - - - - - - - - - - - - - - - - - -  <<<
        Min ΔG for C219 vs C220 is -3.01 kcal/mol.
    - - - - - - - - - - - - - - - - - - - - - - - - - - - - - - - - - - - - - - - -
      5'> CTCCATCTCTACCTCCC >3'
                   |||||
              3'< TATGGATTTGGTTTTTAAAGAAGA <5'
   - - - - - - - - - - - - - - - - - - - - - - - - - - - - - - - - - - - - - - - -
        Min ΔG for C219 vs C219 is 6.63 kcal/mol.
    - - - - - - - - - - - - - - - - - - - - - - - - - - - - - - - - - - - - - - - -
      5'> CTCCATCTCTACCTCCC >3'
                   ||
         3'< CCCTCCATCTCTACCTC <5'
   - - - - - - - - - - - - - - - - - - - - - - - - - - - - - - - - - - - - - - - -
        Min ΔG for C220 vs C220 is -1.59 kcal/mol.
    - - - - - - - - - - - - - - - - - - - - - - - - - - - - - - - - - - - - - - - -
      5'> AGAAGAAATTTTTGGTTTAGGTAT >3'
                                ||
                            3'< TATGGATTTGGTTTTTAAAGAAGA <5'
   - - - - - - - - - - - - - - - - - - - - - - - - - - - - - - - - - - - - - - - -
>>> - - - - - - - - - - - - - - - - - - - - - - - - - - - - - - - - - - - - - - - -  <<<
****************************************************************************************
>>> - - - - - - - - - - - - - - - - - - - - - - - - - - - - - - - - - - - - - - - -  <<<
        Min ΔG for C221 vs C222 is -3.42 kcal/mol.
    - - - - - - - - - - - - - - - - - - - - - - - - - - - - - - - - - - - - - - - -
      5'> GTTGTTTTTGGGAAGATAAGTG >3'
             ||||||   |   | | |
        3'< CCAAAAAAAATAAAAATAAATCAATAC <5'
   - - - - - - - - - - - - - - - - - - - - - - - - - - - - - - - - - - - - - - - -
        Min ΔG for C221 vs C221 is 2.72 kcal/mol.
    - - - - - - - - - - - - - - - - - - - - - - - - - - - - - - - - - - - - - - - -
      5'> GTTGTTTTTGGGAAGATAAGTG >3'
                            | |
                       3'< GTGAATAGAAGGGTTTTTGTTG <5'
   - - - - - - - - - - - - - - - - - - - - - - - - - - - - - - - - - - - - - - - -
        Min ΔG for C222 vs C222 is 6.32 kcal/mol.
    - - - - - - - - - - - - - - - - - - - - - - - - - - - - - - - - - - - - - - - -
      5'> CATAACTAAATAAAAATAAAAAAAACC >3'
                    |    ||    |
           3'< CCAAAAAAAATAAAAATAAATCAATAC <5'
   - - - - - - - - - - - - - - - - - - - - - - - - - - - - - - - - - - - - - - - -
>>> - - - - - - - - - - - - - - - - - - - - - - - - - - - - - - - - - - - - - - - -  <<<
****************************************************************************************
>>> - - - - - - - - - - - - - - - - - - - - - - - - - - - - - - - - - - - - - - - -  <<<
        Min ΔG for C223 vs C224 is -4.78 kcal/mol.
    - - - - - - - - - - - - - - - - - - - - - - - - - - - - - - - - - - - - - - - -
      5'> ATTAGTTTTTTTTTAGGTTTAGGG >3'
                            ||| ||
                        3'< AATACCAAAAAAATTCCAAAAACC <5'
   - - - - - - - - - - - - - - - - - - - - - - - - - - - - - - - - - - - - - - - -
        Min ΔG for C223 vs C223 is 4.96 kcal/mol.
    - - - - - - - - - - - - - - - - - - - - - - - - - - - - - - - - - - - - - - - -
      5'> ATTAGTTTTTTTTTAGGTTTAGGG >3'
                       ||    ||
                3'< GGGATTTGGATTTTTTTTTGATTA <5'
   - - - - - - - - - - - - - - - - - - - - - - - - - - - - - - - - - - - - - - - -
        Min ΔG for C224 vs C224 is 0.88 kcal/mol.
    - - - - - - - - - - - - - - - - - - - - - - - - - - - - - - - - - - - - - - - -
      5'> CCAAAAACCTTAAAAAAACCATAA >3'
                               | |
                           3'< AATACCAAAAAAATTCCAAAAACC <5'
   - - - - - - - - - - - - - - - - - - - - - - - - - - - - - - - - - - - - - - - -
>>> - - - - - - - - - - - - - - - - - - - - - - - - - - - - - - - - - - - - - - - -  <<<
****************************************************************************************
>>> - - - - - - - - - - - - - - - - - - - - - - - - - - - - - - - - - - - - - - - -  <<<
        Min ΔG for C225 vs C226 is -4.16 kcal/mol.
    - - - - - - - - - - - - - - - - - - - - - - - - - - - - - - - - - - - - - - - -
      5'> AAAATTCCTAAACTCTAAACATCA >3'
              |||||   |  ||  |
          3'< AAGGAGGGGGAATAGGG <5'
   - - - - - - - - - - - - - - - - - - - - - - - - - - - - - - - - - - - - - - - -
        Min ΔG for C225 vs C225 is 1.6 kcal/mol.
    - - - - - - - - - - - - - - - - - - - - - - - - - - - - - - - - - - - - - - - -
      5'> AAAATTCCTAAACTCTAAACATCA >3'
                               | |
                           3'< ACTACAAATCTCAAATCCTTAAAA <5'
   - - - - - - - - - - - - - - - - - - - - - - - - - - - - - - - - - - - - - - - -
        Min ΔG for C226 vs C226 is 4.59 kcal/mol.
    - - - - - - - - - - - - - - - - - - - - - - - - - - - - - - - - - - - - - - - -
      5'> GGGATAAGGGGGAGGAA >3'
              |           |
          3'< AAGGAGGGGGAATAGGG <5'
   - - - - - - - - - - - - - - - - - - - - - - - - - - - - - - - - - - - - - - - -
>>> - - - - - - - - - - - - - - - - - - - - - - - - - - - - - - - - - - - - - - - -  <<<
```

```
****************************************************************************************
>>> - - - - - - - - - - - - - - - - - - - - - - - - - - - - - - - - - - - - - - - -  <<<
        Min ΔG for C227 vs C228 is -2.82 kcal/mol.
    - - - - - - - - - - - - - - - - - - - - - - - - - - - - - - - - - - - - - - - -
      5'> GTTATATTGGTGAGGAGGG >3'
                 ||||      |
              3'< CCACAAAAACAATTAAAATCAAAAA <5'
   - - - - - - - - - - - - - - - - - - - - - - - - - - - - - - - - - - - - - - - -
        Min ΔG for C227 vs C227 is 5.16 kcal/mol.
    - - - - - - - - - - - - - - - - - - - - - - - - - - - - - - - - - - - - - - - -
      5'> GTTATATTGGTGAGGAGGG >3'
                 |   | | |
          3'< GGGAGGAGTGGTTATATTG <5'
   - - - - - - - - - - - - - - - - - - - - - - - - - - - - - - - - - - - - - - - -
        Min ΔG for C228 vs C228 is 4.58 kcal/mol.
    - - - - - - - - - - - - - - - - - - - - - - - - - - - - - - - - - - - - - - - -
      5'> AAAAACTAAAATTAACAAAAACACC >3'
                     ||      ||
             3'< CCACAAAAACAATTAAAATCAAAAA <5'
   - - - - - - - - - - - - - - - - - - - - - - - - - - - - - - - - - - - - - - - -
>>> - - - - - - - - - - - - - - - - - - - - - - - - - - - - - - - - - - - - - - - -  <<<
****************************************************************************************
>>> - - - - - - - - - - - - - - - - - - - - - - - - - - - - - - - - - - - - - - - -  <<<
        Min ΔG for C229 vs C230 is -5.26 kcal/mol.
    - - - - - - - - - - - - - - - - - - - - - - - - - - - - - - - - - - - - - - - -
      5'> GAAGGAAGTATTTATTGTTTATAG >3'
                     ||   |||| |||
               3'< CCAAACCCAAAAATCTAAAACA <5'
   - - - - - - - - - - - - - - - - - - - - - - - - - - - - - - - - - - - - - - - -
        Min ΔG for C229 vs C229 is 0.84 kcal/mol.
    - - - - - - - - - - - - - - - - - - - - - - - - - - - - - - - - - - - - - - - -
      5'> GAAGGAAGTATTTATTGTTTATAG >3'
                             ||||
                        3'< GATATTTGTTATTTATGAAGGAAG <5'
   - - - - - - - - - - - - - - - - - - - - - - - - - - - - - - - - - - - - - - - -
        Min ΔG for C230 vs C230 is 6.59 kcal/mol.
    - - - - - - - - - - - - - - - - - - - - - - - - - - - - - - - - - - - - - - - -
      5'> ACAAAATCTAAAAACCCAAACC >3'
                  |         |
           3'< CCAAACCCAAAAATCTAAAACA <5'
   - - - - - - - - - - - - - - - - - - - - - - - - - - - - - - - - - - - - - - - -
>>> - - - - - - - - - - - - - - - - - - - - - - - - - - - - - - - - - - - - - - - -  <<<
****************************************************************************************
>>> - - - - - - - - - - - - - - - - - - - - - - - - - - - - - - - - - - - - - - - -  <<<
        Min ΔG for C231 vs C232 is -5.89 kcal/mol.
    - - - - - - - - - - - - - - - - - - - - - - - - - - - - - - - - - - - - - - - -
      5'> GAAGAGTTTGTAGAGGAGG >3'
                      ||| |||
                  3'< CTCTTCCAATACAACCTATC <5'
   - - - - - - - - - - - - - - - - - - - - - - - - - - - - - - - - - - - - - - - -
        Min ΔG for C231 vs C231 is 4.6 kcal/mol.
    - - - - - - - - - - - - - - - - - - - - - - - - - - - - - - - - - - - - - - - -
      5'> GAAGAGTTTGTAGAGGAGG >3'
                 |  |  |  |
           3'< GGAGGAGATGTTTGAGAAG <5'
   - - - - - - - - - - - - - - - - - - - - - - - - - - - - - - - - - - - - - - - -
        Min ΔG for C232 vs C232 is 5.44 kcal/mol.
    - - - - - - - - - - - - - - - - - - - - - - - - - - - - - - - - - - - - - - - -
      5'> CTATCCAACATAACCTTCTC >3'
                   | ||  || |
              3'< CTCTTCCAATACAACCTATC <5'
   - - - - - - - - - - - - - - - - - - - - - - - - - - - - - - - - - - - - - - - -
>>> - - - - - - - - - - - - - - - - - - - - - - - - - - - - - - - - - - - - - - - -  <<<
****************************************************************************************
>>> - - - - - - - - - - - - - - - - - - - - - - - - - - - - - - - - - - - - - - - -  <<<
        Min ΔG for C233 vs C234 is -2.79 kcal/mol.
    - - - - - - - - - - - - - - - - - - - - - - - - - - - - - - - - - - - - - - - -
      5'> TACTTTCTCTAACACCTCCC >3'
                        | | ||
                    3'< GTATGGATAGTGGTTGGGA <5'
   - - - - - - - - - - - - - - - - - - - - - - - - - - - - - - - - - - - - - - - -
        Min ΔG for C233 vs C233 is 6.78 kcal/mol.
    - - - - - - - - - - - - - - - - - - - - - - - - - - - - - - - - - - - - - - - -
      5'> TACTTTCTCTAACACCTCCC >3'
                   ||
      3'< CCCTCCACAATCTCTTTCAT <5'
   - - - - - - - - - - - - - - - - - - - - - - - - - - - - - - - - - - - - - - - -
        Min ΔG for C234 vs C234 is 0.48 kcal/mol.
    - - - - - - - - - - - - - - - - - - - - - - - - - - - - - - - - - - - - - - - -
      5'> AGGGTTGGTGATAGGTATG >3'
                    |||  |||
               3'< GTATGGATAGTGGTTGGGA <5'
   - - - - - - - - - - - - - - - - - - - - - - - - - - - - - - - - - - - - - - - -
>>> - - - - - - - - - - - - - - - - - - - - - - - - - - - - - - - - - - - - - - - -  <<<
```

```
****************************************************************************************
>>> - - - - - - - - - - - - - - - - - - - - - - - - - - - - - - - - - - - - - - - -  <<<
        Min ΔG for C235 vs C236 is 0.02 kcal/mol.
    - - - - - - - - - - - - - - - - - - - - - - - - - - - - - - - - - - - - - - -
      5'> GGATAGAGAGAGAAAAGAAG >3'
                            |
                        3'< TACACTAATAAACAAATTCCAATAT <5'
   - - - - - - - - - - - - - - - - - - - - - - - - - - - - - - - - - - - - - - - -
        Min ΔG for C235 vs C235 is 5.73 kcal/mol.
    - - - - - - - - - - - - - - - - - - - - - - - - - - - - - - - - - - - - - - -
      5'> GGATAGAGAGAGAAAAGAAG >3'
             |              |
        3'< GAAGAAAAGAGAGAGATAGG <5'
   - - - - - - - - - - - - - - - - - - - - - - - - - - - - - - - - - - - - - - - -
        Min ΔG for C236 vs C236 is -1.3 kcal/mol.
    - - - - - - - - - - - - - - - - - - - - - - - - - - - - - - - - - - - - - - -
      5'> TATAACCTTAAACAAATAATCACAT >3'
                                 ||
                             3'< TACACTAATAAACAAATTCCAATAT <5'
   - - - - - - - - - - - - - - - - - - - - - - - - - - - - - - - - - - - - - - - -
>>> - - - - - - - - - - - - - - - - - - - - - - - - - - - - - - - - - - - - - - - -  <<<
****************************************************************************************
>>> - - - - - - - - - - - - - - - - - - - - - - - - - - - - - - - - - - - - - - - -  <<<
        Min ΔG for C237 vs C238 is -5.16 kcal/mol.
    - - - - - - - - - - - - - - - - - - - - - - - - - - - - - - - - - - - - - - -
      5'> TTTCCAACCCAATTTTCCAAC >3'
              ||    || | |||||
          3'< GTGAATTTGAGAGGTTTTGTAAAT <5'
   - - - - - - - - - - - - - - - - - - - - - - - - - - - - - - - - - - - - - - - -
        Min ΔG for C237 vs C237 is 3.08 kcal/mol.
    - - - - - - - - - - - - - - - - - - - - - - - - - - - - - - - - - - - - - - -
      5'> TTTCCAACCCAATTTTCCAAC >3'
           ||  ||       ||  ||
      3'< CAACCTTTTAACCCAACCTTT <5'
   - - - - - - - - - - - - - - - - - - - - - - - - - - - - - - - - - - - - - - - -
        Min ΔG for C238 vs C238 is 1.0 kcal/mol.
    - - - - - - - - - - - - - - - - - - - - - - - - - - - - - - - - - - - - - - -
      5'> TAAATGTTTTGGAGAGTTTAAGTG >3'
                        | || || |
                   3'< GTGAATTTGAGAGGTTTTGTAAAT <5'
   - - - - - - - - - - - - - - - - - - - - - - - - - - - - - - - - - - - - - - - -
>>> - - - - - - - - - - - - - - - - - - - - - - - - - - - - - - - - - - - - - - - -  <<<
****************************************************************************************
>>> - - - - - - - - - - - - - - - - - - - - - - - - - - - - - - - - - - - - - - - -  <<<
        Min ΔG for C239 vs C240 is -2.42 kcal/mol.
    - - - - - - - - - - - - - - - - - - - - - - - - - - - - - - - - - - - - - - -
      5'> CAACTATTACTATAAAATAAACATTAA >3'
                               |  |||
                           3'< GAGATTGGTTGATTTGATTGG <5'
   - - - - - - - - - - - - - - - - - - - - - - - - - - - - - - - - - - - - - - - -
        Min ΔG for C239 vs C239 is -3.14 kcal/mol.
    - - - - - - - - - - - - - - - - - - - - - - - - - - - - - - - - - - - - - - -
      5'> CAACTATTACTATAAAATAAACATTAA >3'
                                 ||||
                             3'< AATTACAAATAAAATATCATTATCAAC <5'
   - - - - - - - - - - - - - - - - - - - - - - - - - - - - - - - - - - - - - - - -
        Min ΔG for C240 vs C240 is 3.41 kcal/mol.
    - - - - - - - - - - - - - - - - - - - - - - - - - - - - - - - - - - - - - - -
      5'> GGTTAGTTTAGTTGGTTAGAG >3'
               | ||       || |
           3'< GAGATTGGTTGATTTGATTGG <5'
   - - - - - - - - - - - - - - - - - - - - - - - - - - - - - - - - - - - - - - - -
>>> - - - - - - - - - - - - - - - - - - - - - - - - - - - - - - - - - - - - - - - -  <<<
****************************************************************************************
>>> - - - - - - - - - - - - - - - - - - - - - - - - - - - - - - - - - - - - - - - -  <<<
        Min ΔG for C241 vs C242 is -1.42 kcal/mol.
    - - - - - - - - - - - - - - - - - - - - - - - - - - - - - - - - - - - - - - -
      5'> CACTCCTTTCTACTTTACTTC >3'
                             ||
                         3'< AGTTTTGGTTATATTTAGGGGT <5'
   - - - - - - - - - - - - - - - - - - - - - - - - - - - - - - - - - - - - - - - -
        Min ΔG for C241 vs C241 is 4.82 kcal/mol.
    - - - - - - - - - - - - - - - - - - - - - - - - - - - - - - - - - - - - - - -
      5'> CACTCCTTTCTACTTTACTTC >3'
                         ||
                 3'< CTTCATTTCATCTTTCCTCAC <5'
   - - - - - - - - - - - - - - - - - - - - - - - - - - - - - - - - - - - - - - - -
        Min ΔG for C242 vs C242 is 0.78 kcal/mol.
    - - - - - - - - - - - - - - - - - - - - - - - - - - - - - - - - - - - - - - -
      5'> TGGGGATTTATATTGGTTTTGA >3'
                             | |
                         3'< AGTTTTGGTTATATTTAGGGGT <5'
   - - - - - - - - - - - - - - - - - - - - - - - - - - - - - - - - - - - - - - - -
>>> - - - - - - - - - - - - - - - - - - - - - - - - - - - - - - - - - - - - - - - -  <<<
```

```
****************************************************************************************
>>> - - - - - - - - - - - - - - - - - - - - - - - - - - - - - - - - - - - - - - - -  <<<
        Min ΔG for C243 vs C244 is -9.73 kcal/mol.
    - - - - - - - - - - - - - - - - - - - - - - - - - - - - - - - - - - - - - - - -
      5'> ACCAATATAAATCCCCACCC >3'
                      ||||||||
              3'< GGATGGGGTGGGGTAAAAA <5'
   - - - - - - - - - - - - - - - - - - - - - - - - - - - - - - - - - - - - - - - -
        Min ΔG for C243 vs C243 is 6.71 kcal/mol.
    - - - - - - - - - - - - - - - - - - - - - - - - - - - - - - - - - - - - - - - -
      5'> ACCAATATAAATCCCCACCC >3'
               |    ||    |
        3'< CCCACCCCTAAATATAACCA <5'
   - - - - - - - - - - - - - - - - - - - - - - - - - - - - - - - - - - - - - - - -
        Min ΔG for C244 vs C244 is 2.4 kcal/mol.
    - - - - - - - - - - - - - - - - - - - - - - - - - - - - - - - - - - - - - - - -
      5'> AAAAATGGGGTGGGGTAGG >3'
                         ||
                   3'< GGATGGGGTGGGGTAAAAA <5'
   - - - - - - - - - - - - - - - - - - - - - - - - - - - - - - - - - - - - - - - -
>>> - - - - - - - - - - - - - - - - - - - - - - - - - - - - - - - - - - - - - - - -  <<<
****************************************************************************************
>>> - - - - - - - - - - - - - - - - - - - - - - - - - - - - - - - - - - - - - - - -  <<<
        Min ΔG for C245 vs C246 is -2.88 kcal/mol.
    - - - - - - - - - - - - - - - - - - - - - - - - - - - - - - - - - - - - - - - -
      5'> TAACTTCTCTTTTTAACTTCTCTA >3'
                  ||| ||| | |
              3'< GAAGAATAGTATTTTAATTGAAGG <5'
   - - - - - - - - - - - - - - - - - - - - - - - - - - - - - - - - - - - - - - - -
        Min ΔG for C245 vs C245 is -0.62 kcal/mol.
    - - - - - - - - - - - - - - - - - - - - - - - - - - - - - - - - - - - - - - - -
      5'> TAACTTCTCTTTTTAACTTCTCTA >3'
                                ||
                            3'< ATCTCTTCAATTTTTCTCTTCAAT <5'
   - - - - - - - - - - - - - - - - - - - - - - - - - - - - - - - - - - - - - - - -
        Min ΔG for C246 vs C246 is 1.54 kcal/mol.
    - - - - - - - - - - - - - - - - - - - - - - - - - - - - - - - - - - - - - - - -
      5'> GGAAGTTAATTTTATGATAAGAAG >3'
                   | |||| |||| |
             3'< GAAGAATAGTATTTTAATTGAAGG <5'
   - - - - - - - - - - - - - - - - - - - - - - - - - - - - - - - - - - - - - - - -
>>> - - - - - - - - - - - - - - - - - - - - - - - - - - - - - - - - - - - - - - - -  <<<
****************************************************************************************
>>> - - - - - - - - - - - - - - - - - - - - - - - - - - - - - - - - - - - - - - - -  <<<
        Min ΔG for C247 vs C248 is -4.08 kcal/mol.
    - - - - - - - - - - - - - - - - - - - - - - - - - - - - - - - - - - - - - - - -
      5'> TCTTATCATAAAATTAACTTCCCT >3'
                           | || ||
                       3'< GTAGAGATTTTTTTTTTATTGGTG <5'
   - - - - - - - - - - - - - - - - - - - - - - - - - - - - - - - - - - - - - - - -
        Min ΔG for C247 vs C247 is 3.11 kcal/mol.
    - - - - - - - - - - - - - - - - - - - - - - - - - - - - - - - - - - - - - - - -
      5'> TCTTATCATAAAATTAACTTCCCT >3'
                 |   || | | ||   |
             3'< TCCCTTCAATTAAAATACTATTCT <5'
   - - - - - - - - - - - - - - - - - - - - - - - - - - - - - - - - - - - - - - - -
        Min ΔG for C248 vs C248 is 1.5 kcal/mol.
    - - - - - - - - - - - - - - - - - - - - - - - - - - - - - - - - - - - - - - - -
      5'> GTGGTTATTTTTTTTTTAGAGATG >3'
                               ||
                          3'< GTAGAGATTTTTTTTTTATTGGTG <5'
   - - - - - - - - - - - - - - - - - - - - - - - - - - - - - - - - - - - - - - - -
>>> - - - - - - - - - - - - - - - - - - - - - - - - - - - - - - - - - - - - - - - -  <<<
****************************************************************************************
>>> - - - - - - - - - - - - - - - - - - - - - - - - - - - - - - - - - - - - - - - -  <<<
        Min ΔG for C249 vs C250 is -4.08 kcal/mol.
    - - - - - - - - - - - - - - - - - - - - - - - - - - - - - - - - - - - - - - - -
      5'> TATATCTATCCCAAAAAAAAACCT >3'
                               |||
                           3'< GGAAAGGAGAAAAAGAAAGAATAA <5'
   - - - - - - - - - - - - - - - - - - - - - - - - - - - - - - - - - - - - - - - -
        Min ΔG for C249 vs C249 is 3.01 kcal/mol.
    - - - - - - - - - - - - - - - - - - - - - - - - - - - - - - - - - - - - - - - -
      5'> TATATCTATCCCAAAAAAAAACCT >3'
                             |   |
                         3'< TCCAAAAAAAAACCCTATCTATAT <5'
   - - - - - - - - - - - - - - - - - - - - - - - - - - - - - - - - - - - - - - - -
        Min ΔG for C250 vs C250 is 5.73 kcal/mol.
    - - - - - - - - - - - - - - - - - - - - - - - - - - - - - - - - - - - - - - - -
      5'> AATAAGAAAGAAAAAGAGGAAAGG >3'
            |                  |
      3'< GGAAAGGAGAAAAAGAAAGAATAA <5'
   - - - - - - - - - - - - - - - - - - - - - - - - - - - - - - - - - - - - - - - -
>>> - - - - - - - - - - - - - - - - - - - - - - - - - - - - - - - - - - - - - - - -  <<<
```

```
****************************************************************************************
>>> - - - - - - - - - - - - - - - - - - - - - - - - - - - - - - - - - - - - - - - -  <<<
        Min ΔG for C251 vs C252 is -7.73 kcal/mol.
    - - - - - - - - - - - - - - - - - - - - - - - - - - - - - - - - - - - - - - - -
      5'> CTAAACTTTTTAACCCTTTCCT >3'
                       || |||||
                  3'< AAGGGAAGGAAGTGAGTTTT <5'
   - - - - - - - - - - - - - - - - - - - - - - - - - - - - - - - - - - - - - - - -
        Min ΔG for C251 vs C251 is 4.57 kcal/mol.
    - - - - - - - - - - - - - - - - - - - - - - - - - - - - - - - - - - - - - - - -
      5'> CTAAACTTTTTAACCCTTTCCT >3'
            |                  |
        3'< TCCTTTCCCAATTTTTCAAATC <5'
   - - - - - - - - - - - - - - - - - - - - - - - - - - - - - - - - - - - - - - - -
        Min ΔG for C252 vs C252 is 1.69 kcal/mol.
    - - - - - - - - - - - - - - - - - - - - - - - - - - - - - - - - - - - - - - - -
      5'> TTTTGAGTGAAGGAAGGGAA >3'
            ||   |      |   ||
        3'< AAGGGAAGGAAGTGAGTTTT <5'
   - - - - - - - - - - - - - - - - - - - - - - - - - - - - - - - - - - - - - - - -
>>> - - - - - - - - - - - - - - - - - - - - - - - - - - - - - - - - - - - - - - - -  <<<
****************************************************************************************
>>> - - - - - - - - - - - - - - - - - - - - - - - - - - - - - - - - - - - - - - - -  <<<
        Min ΔG for C253 vs C254 is -1.96 kcal/mol.
    - - - - - - - - - - - - - - - - - - - - - - - - - - - - - - - - - - - - - - - -
      5'> GTTTTTTTTTTTTATTTAGGATGGT >3'
                               | ||
                           3'< AACATATCTTCCAAAAACCATC <5'
   - - - - - - - - - - - - - - - - - - - - - - - - - - - - - - - - - - - - - - - -
        Min ΔG for C253 vs C253 is 0.92 kcal/mol.
    - - - - - - - - - - - - - - - - - - - - - - - - - - - - - - - - - - - - - - - -
      5'> GTTTTTTTTTTTTATTTAGGATGGT >3'
                           |  ||  |
                       3'< TGGTAGGATTTATTTTTTTTTTTTG <5'
   - - - - - - - - - - - - - - - - - - - - - - - - - - - - - - - - - - - - - - - -
        Min ΔG for C254 vs C254 is -0.74 kcal/mol.
    - - - - - - - - - - - - - - - - - - - - - - - - - - - - - - - - - - - - - - - -
      5'> CTACCAAAAACCTTCTATACAA >3'
                      || |||| ||
                  3'< AACATATCTTCCAAAAACCATC <5'
   - - - - - - - - - - - - - - - - - - - - - - - - - - - - - - - - - - - - - - - -
>>> - - - - - - - - - - - - - - - - - - - - - - - - - - - - - - - - - - - - - - - -  <<<
****************************************************************************************
>>> - - - - - - - - - - - - - - - - - - - - - - - - - - - - - - - - - - - - - - - -  <<<
        Min ΔG for C255 vs C256 is -2.52 kcal/mol.
    - - - - - - - - - - - - - - - - - - - - - - - - - - - - - - - - - - - - - - - -
      5'> GGTGTGTGTGTGTGTGTG >3'
                   |   || |||
              3'< CCCAACCCACCAAATATTAA <5'
   - - - - - - - - - - - - - - - - - - - - - - - - - - - - - - - - - - - - - - - -
    - - - - - - - - - - - - - - - - - - - - - - - - - - - - - - - - - - - - - - - -
       No dimers found for C255 vs C255
    - - - - - - - - - - - - - - - - - - - - - - - - - - - - - - - - - - - - - - - -
        Min ΔG for C256 vs C256 is 6.35 kcal/mol.
    - - - - - - - - - - - - - - - - - - - - - - - - - - - - - - - - - - - - - - - -
      5'> AATTATAAACCACCCAACCC >3'
             |            |
      3'< CCCAACCCACCAAATATTAA <5'
   - - - - - - - - - - - - - - - - - - - - - - - - - - - - - - - - - - - - - - - -
>>> - - - - - - - - - - - - - - - - - - - - - - - - - - - - - - - - - - - - - - - -  <<<
****************************************************************************************
>>> - - - - - - - - - - - - - - - - - - - - - - - - - - - - - - - - - - - - - - - -  <<<
        Min ΔG for C257 vs C258 is -5.33 kcal/mol.
    - - - - - - - - - - - - - - - - - - - - - - - - - - - - - - - - - - - - - - - -
      5'> TAGTTGTTTTTTTAAAAAAAAGTGG >3'
                               ||||
                           3'< CACCCCTCTATTCATCTCAT <5'
   - - - - - - - - - - - - - - - - - - - - - - - - - - - - - - - - - - - - - - - -
        Min ΔG for C257 vs C257 is 0.95 kcal/mol.
    - - - - - - - - - - - - - - - - - - - - - - - - - - - - - - - - - - - - - - - -
      5'> TAGTTGTTTTTTTAAAAAAAAGTGG >3'
                |||||||  |||||||
        3'< GGTGAAAAAAAATTTTTTTGTTGAT <5'
   - - - - - - - - - - - - - - - - - - - - - - - - - - - - - - - - - - - - - - - -
        Min ΔG for C258 vs C258 is 6.58 kcal/mol.
    - - - - - - - - - - - - - - - - - - - - - - - - - - - - - - - - - - - - - - - -
      5'> TACTCTACTTATCTCCCCAC >3'
                   |        |
              3'< CACCCCTCTATTCATCTCAT <5'
   - - - - - - - - - - - - - - - - - - - - - - - - - - - - - - - - - - - - - - - -
>>> - - - - - - - - - - - - - - - - - - - - - - - - - - - - - - - - - - - - - - - -  <<<
```

```
****************************************************************************************
>>> - - - - - - - - - - - - - - - - - - - - - - - - - - - - - - - - - - - - - - - -  <<<
        Min ΔG for C259 vs C260 is -5.12 kcal/mol.
    - - - - - - - - - - - - - - - - - - - - - - - - - - - - - - - - - - - - - - - -
      5'> TATTTAAATAAACAAACTAAACCCA >3'
                               ||||
                          3'< GGGGTGTGATAGATGTTTG <5'
   - - - - - - - - - - - - - - - - - - - - - - - - - - - - - - - - - - - - - - - -
        Min ΔG for C259 vs C259 is 3.78 kcal/mol.
    - - - - - - - - - - - - - - - - - - - - - - - - - - - - - - - - - - - - - - - -
      5'> TATTTAAATAAACAAACTAAACCCA >3'
            |      ||      ||     |
        3'< ACCCAAATCAAACAAATAAATTTAT <5'
   - - - - - - - - - - - - - - - - - - - - - - - - - - - - - - - - - - - - - - - -
        Min ΔG for C260 vs C260 is 4.88 kcal/mol.
    - - - - - - - - - - - - - - - - - - - - - - - - - - - - - - - - - - - - - - - -
      5'> GTTTGTAGATAGTGTGGGG >3'
                | |   | |
        3'< GGGGTGTGATAGATGTTTG <5'
   - - - - - - - - - - - - - - - - - - - - - - - - - - - - - - - - - - - - - - - -
>>> - - - - - - - - - - - - - - - - - - - - - - - - - - - - - - - - - - - - - - - -  <<<
****************************************************************************************
>>> - - - - - - - - - - - - - - - - - - - - - - - - - - - - - - - - - - - - - - - -  <<<
        Min ΔG for C261 vs C262 is -3.34 kcal/mol.
    - - - - - - - - - - - - - - - - - - - - - - - - - - - - - - - - - - - - - - - -
      5'> GTAGGGAAATAGTTTTGTTTGG >3'
                         |||||
                     3'< ACAAAAAATTAAATTTCCAATACTC <5'
   - - - - - - - - - - - - - - - - - - - - - - - - - - - - - - - - - - - - - - - -
        Min ΔG for C261 vs C261 is 1.28 kcal/mol.
    - - - - - - - - - - - - - - - - - - - - - - - - - - - - - - - - - - - - - - - -
      5'> GTAGGGAAATAGTTTTGTTTGG >3'
                |||  |    | |||
          3'< GGTTTGTTTTGATAAAGGGATG <5'
   - - - - - - - - - - - - - - - - - - - - - - - - - - - - - - - - - - - - - - - -
        Min ΔG for C262 vs C262 is 1.9 kcal/mol.
    - - - - - - - - - - - - - - - - - - - - - - - - - - - - - - - - - - - - - - - -
      5'> CTCATAACCTTTAAATTAAAAAACA >3'
              |    ||||| |||||    |
          3'< ACAAAAAATTAAATTTCCAATACTC <5'
   - - - - - - - - - - - - - - - - - - - - - - - - - - - - - - - - - - - - - - - -
>>> - - - - - - - - - - - - - - - - - - - - - - - - - - - - - - - - - - - - - - - -  <<<
****************************************************************************************
>>> - - - - - - - - - - - - - - - - - - - - - - - - - - - - - - - - - - - - - - - -  <<<
        Min ΔG for C263 vs C264 is -12.07 kcal/mol.
    - - - - - - - - - - - - - - - - - - - - - - - - - - - - - - - - - - - - - - - -
      5'> TTAAAAATGGGGTGGGGTAG >3'
                   ||||||||
               3'< CCCACCCCTAAATATAACC <5'
   - - - - - - - - - - - - - - - - - - - - - - - - - - - - - - - - - - - - - - - -
        Min ΔG for C263 vs C263 is 0.64 kcal/mol.
    - - - - - - - - - - - - - - - - - - - - - - - - - - - - - - - - - - - - - - - -
      5'> TTAAAAATGGGGTGGGGTAG >3'
                           ||
                      3'< GATGGGGTGGGGTAAAAATT <5'
   - - - - - - - - - - - - - - - - - - - - - - - - - - - - - - - - - - - - - - - -
        Min ΔG for C264 vs C264 is 6.71 kcal/mol.
    - - - - - - - - - - - - - - - - - - - - - - - - - - - - - - - - - - - - - - - -
      5'> CCAATATAAATCCCCACCC >3'
             |     ||    |
       3'< CCCACCCCTAAATATAACC <5'
   - - - - - - - - - - - - - - - - - - - - - - - - - - - - - - - - - - - - - - - -
>>> - - - - - - - - - - - - - - - - - - - - - - - - - - - - - - - - - - - - - - - -  <<<
****************************************************************************************
>>> - - - - - - - - - - - - - - - - - - - - - - - - - - - - - - - - - - - - - - - -  <<<
        Min ΔG for C265 vs C266 is -1.93 kcal/mol.
    - - - - - - - - - - - - - - - - - - - - - - - - - - - - - - - - - - - - - - - -
      5'> TGGGGATTTATATTGGTTTTGA >3'
                              ||
                          3'< CTTCATTTCATCTTTCCTCAC <5'
   - - - - - - - - - - - - - - - - - - - - - - - - - - - - - - - - - - - - - - - -
        Min ΔG for C265 vs C265 is 0.78 kcal/mol.
    - - - - - - - - - - - - - - - - - - - - - - - - - - - - - - - - - - - - - - - -
      5'> TGGGGATTTATATTGGTTTTGA >3'
                             | |
                         3'< AGTTTTGGTTATATTTAGGGGT <5'
   - - - - - - - - - - - - - - - - - - - - - - - - - - - - - - - - - - - - - - - -
        Min ΔG for C266 vs C266 is 4.82 kcal/mol.
    - - - - - - - - - - - - - - - - - - - - - - - - - - - - - - - - - - - - - - - -
      5'> CACTCCTTTCTACTTTACTTC >3'
                         ||
                 3'< CTTCATTTCATCTTTCCTCAC <5'
   - - - - - - - - - - - - - - - - - - - - - - - - - - - - - - - - - - - - - - - -
>>> - - - - - - - - - - - - - - - - - - - - - - - - - - - - - - - - - - - - - - - -  <<<
```

```
****************************************************************************************
>>> - - - - - - - - - - - - - - - - - - - - - - - - - - - - - - - - - - - - - - - -  <<<
        Min ΔG for C267 vs C268 is -1.75 kcal/mol.
    - - - - - - - - - - - - - - - - - - - - - - - - - - - - - - - - - - - - - - - -
      5'> GGTTAGTTTAGTTGGTTAGAG >3'
                 |   |  ||| ||
          3'< ATTACAAATAAAATATCATTATTAATAC <5'
   - - - - - - - - - - - - - - - - - - - - - - - - - - - - - - - - - - - - - - - -
        Min ΔG for C267 vs C267 is 3.41 kcal/mol.
    - - - - - - - - - - - - - - - - - - - - - - - - - - - - - - - - - - - - - - - -
      5'> GGTTAGTTTAGTTGGTTAGAG >3'
               | ||       || |
           3'< GAGATTGGTTGATTTGATTGG <5'
   - - - - - - - - - - - - - - - - - - - - - - - - - - - - - - - - - - - - - - - -
        Min ΔG for C268 vs C268 is -1.78 kcal/mol.
    - - - - - - - - - - - - - - - - - - - - - - - - - - - - - - - - - - - - - - - -
      5'> CATAATTATTACTATAAAATAAACATTA >3'
            ||||  ||| | || | |||  ||||
        3'< ATTACAAATAAAATATCATTATTAATAC <5'
   - - - - - - - - - - - - - - - - - - - - - - - - - - - - - - - - - - - - - - - -
>>> - - - - - - - - - - - - - - - - - - - - - - - - - - - - - - - - - - - - - - - -  <<<
****************************************************************************************
>>> - - - - - - - - - - - - - - - - - - - - - - - - - - - - - - - - - - - - - - - -  <<<
        Min ΔG for C269 vs C270 is -3.47 kcal/mol.
    - - - - - - - - - - - - - - - - - - - - - - - - - - - - - - - - - - - - - - - -
      5'> AAAAAAACTTTTCTTCCCAAACAA >3'
                         |||  ||
                     3'< GGGGGTGGAATGTGAGA <5'
   - - - - - - - - - - - - - - - - - - - - - - - - - - - - - - - - - - - - - - - -
        Min ΔG for C269 vs C269 is 1.42 kcal/mol.
    - - - - - - - - - - - - - - - - - - - - - - - - - - - - - - - - - - - - - - - -
      5'> AAAAAAACTTTTCTTCCCAAACAA >3'
                   ||  ||   ||  ||
               3'< AACAAACCCTTCTTTTCAAAAAAA <5'
   - - - - - - - - - - - - - - - - - - - - - - - - - - - - - - - - - - - - - - - -
        Min ΔG for C270 vs C270 is 5.59 kcal/mol.
    - - - - - - - - - - - - - - - - - - - - - - - - - - - - - - - - - - - - - - - -
      5'> AGAGTGTAAGGTGGGGG >3'
                 |   |
        3'< GGGGGTGGAATGTGAGA <5'
   - - - - - - - - - - - - - - - - - - - - - - - - - - - - - - - - - - - - - - - -
>>> - - - - - - - - - - - - - - - - - - - - - - - - - - - - - - - - - - - - - - - -  <<<
****************************************************************************************
>>> - - - - - - - - - - - - - - - - - - - - - - - - - - - - - - - - - - - - - - - -  <<<
        Min ΔG for C271 vs C272 is -1.06 kcal/mol.
    - - - - - - - - - - - - - - - - - - - - - - - - - - - - - - - - - - - - - - - -
      5'> ATTAGGTAGAGGTGGGTGG >3'
            |||| | |||
        3'< ATCCCTTTCCCTAACAAAAAAA <5'
   - - - - - - - - - - - - - - - - - - - - - - - - - - - - - - - - - - - - - - - -
        Min ΔG for C271 vs C271 is 4.87 kcal/mol.
    - - - - - - - - - - - - - - - - - - - - - - - - - - - - - - - - - - - - - - - -
      5'> ATTAGGTAGAGGTGGGTGG >3'
             |   |    |   |
       3'< GGTGGGTGGAGATGGATTA <5'
   - - - - - - - - - - - - - - - - - - - - - - - - - - - - - - - - - - - - - - - -
        Min ΔG for C272 vs C272 is -0.62 kcal/mol.
    - - - - - - - - - - - - - - - - - - - - - - - - - - - - - - - - - - - - - - - -
      5'> AAAAAAACAATCCCTTTCCCTA >3'
                              ||
                          3'< ATCCCTTTCCCTAACAAAAAAA <5'
   - - - - - - - - - - - - - - - - - - - - - - - - - - - - - - - - - - - - - - - -
>>> - - - - - - - - - - - - - - - - - - - - - - - - - - - - - - - - - - - - - - - -  <<<
****************************************************************************************
>>> - - - - - - - - - - - - - - - - - - - - - - - - - - - - - - - - - - - - - - - -  <<<
        Min ΔG for C273 vs C274 is -0.58 kcal/mol.
    - - - - - - - - - - - - - - - - - - - - - - - - - - - - - - - - - - - - - - - -
      5'> TTGTTTTTTAGGAAAGAGAGAG >3'
                               |
                           3'< CCTACTAAATATAAAACCCTTATT <5'
   - - - - - - - - - - - - - - - - - - - - - - - - - - - - - - - - - - - - - - - -
        Min ΔG for C273 vs C273 is 3.81 kcal/mol.
    - - - - - - - - - - - - - - - - - - - - - - - - - - - - - - - - - - - - - - - -
      5'> TTGTTTTTTAGGAAAGAGAGAG >3'
             | | |        | | |
        3'< GAGAGAGAAAGGATTTTTTGTT <5'
   - - - - - - - - - - - - - - - - - - - - - - - - - - - - - - - - - - - - - - - -
        Min ΔG for C274 vs C274 is 4.05 kcal/mol.
    - - - - - - - - - - - - - - - - - - - - - - - - - - - - - - - - - - - - - - - -
      5'> TTATTCCCAAAATATAAATCATCC >3'
                           || ||
                     3'< CCTACTAAATATAAAACCCTTATT <5'
   - - - - - - - - - - - - - - - - - - - - - - - - - - - - - - - - - - - - - - - -
>>> - - - - - - - - - - - - - - - - - - - - - - - - - - - - - - - - - - - - - - - -  <<<
```

```
****************************************************************************************
>>> - - - - - - - - - - - - - - - - - - - - - - - - - - - - - - - - - - - - - - - -  <<<
        Min ΔG for C275 vs C276 is -2.31 kcal/mol.
    - - - - - - - - - - - - - - - - - - - - - - - - - - - - - - - - - - - - - - - -
      5'> GGTGGAGTTTATTAAGGTTG >3'
                         || ||
                    3'< CCCCACACCAATATCCC <5'
   - - - - - - - - - - - - - - - - - - - - - - - - - - - - - - - - - - - - - - - -
        Min ΔG for C275 vs C275 is 0.51 kcal/mol.
    - - - - - - - - - - - - - - - - - - - - - - - - - - - - - - - - - - - - - - - -
      5'> GGTGGAGTTTATTAAGGTTG >3'
                       ||  ||
                  3'< GTTGGAATTATTTGAGGTGG <5'
   - - - - - - - - - - - - - - - - - - - - - - - - - - - - - - - - - - - - - - - -
        Min ΔG for C276 vs C276 is 6.71 kcal/mol.
    - - - - - - - - - - - - - - - - - - - - - - - - - - - - - - - - - - - - - - - -
      5'> CCCTATAACCACACCCC >3'
               |      |
       3'< CCCCACACCAATATCCC <5'
   - - - - - - - - - - - - - - - - - - - - - - - - - - - - - - - - - - - - - - - -
>>> - - - - - - - - - - - - - - - - - - - - - - - - - - - - - - - - - - - - - - - -  <<<
****************************************************************************************
>>> - - - - - - - - - - - - - - - - - - - - - - - - - - - - - - - - - - - - - - - -  <<<
        Min ΔG for C277 vs C278 is -4.6 kcal/mol.
    - - - - - - - - - - - - - - - - - - - - - - - - - - - - - - - - - - - - - - - -
      5'> TGATTAATAAGTGTTTTTTGTTGTG >3'
                          ||| |||||
                    3'< CCAAATAACACCATTCAACTC <5'
   - - - - - - - - - - - - - - - - - - - - - - - - - - - - - - - - - - - - - - - -
        Min ΔG for C277 vs C277 is 2.15 kcal/mol.
    - - - - - - - - - - - - - - - - - - - - - - - - - - - - - - - - - - - - - - - -
      5'> TGATTAATAAGTGTTTTTTGTTGTG >3'
               || ||       || ||
        3'< GTGTTGTTTTTTGTGAATAATTAGT <5'
   - - - - - - - - - - - - - - - - - - - - - - - - - - - - - - - - - - - - - - - -
        Min ΔG for C278 vs C278 is 3.29 kcal/mol.
    - - - - - - - - - - - - - - - - - - - - - - - - - - - - - - - - - - - - - - - -
      5'> CTCAACTTACCACAATAAACC >3'
                |||      |||
         3'< CCAAATAACACCATTCAACTC <5'
   - - - - - - - - - - - - - - - - - - - - - - - - - - - - - - - - - - - - - - - -
>>> - - - - - - - - - - - - - - - - - - - - - - - - - - - - - - - - - - - - - - - -  <<<
****************************************************************************************
>>> - - - - - - - - - - - - - - - - - - - - - - - - - - - - - - - - - - - - - - - -  <<<
        Min ΔG for C279 vs C280 is -2.92 kcal/mol.
    - - - - - - - - - - - - - - - - - - - - - - - - - - - - - - - - - - - - - - - -
      5'> ATAAATGTTTAAGGTGAAATGTGG >3'
                            | ||||
                      3'< CCTTCACCACTCAATATAACAA <5'
   - - - - - - - - - - - - - - - - - - - - - - - - - - - - - - - - - - - - - - - -
        Min ΔG for C279 vs C279 is 2.1 kcal/mol.
    - - - - - - - - - - - - - - - - - - - - - - - - - - - - - - - - - - - - - - - -
      5'> ATAAATGTTTAAGGTGAAATGTGG >3'
              |  ||| |  | |||  |
        3'< GGTGTAAAGTGGAATTTGTAAATA <5'
   - - - - - - - - - - - - - - - - - - - - - - - - - - - - - - - - - - - - - - - -
        Min ΔG for C280 vs C280 is 4.9 kcal/mol.
    - - - - - - - - - - - - - - - - - - - - - - - - - - - - - - - - - - - - - - - -
      5'> AACAATATAACTCACCACTTCC >3'
             ||             ||
       3'< CCTTCACCACTCAATATAACAA <5'
   - - - - - - - - - - - - - - - - - - - - - - - - - - - - - - - - - - - - - - - -
>>> - - - - - - - - - - - - - - - - - - - - - - - - - - - - - - - - - - - - - - - -  <<<
****************************************************************************************
>>> - - - - - - - - - - - - - - - - - - - - - - - - - - - - - - - - - - - - - - - -  <<<
        Min ΔG for C281 vs C282 is -3.15 kcal/mol.
    - - - - - - - - - - - - - - - - - - - - - - - - - - - - - - - - - - - - - - - -
      5'> GAAGTGGTGAGTTATATTGTTA >3'
                         | ||||
                     3'< TTACAAAAACTTATTCAAAACAATC <5'
   - - - - - - - - - - - - - - - - - - - - - - - - - - - - - - - - - - - - - - - -
        Min ΔG for C281 vs C281 is -0.7 kcal/mol.
    - - - - - - - - - - - - - - - - - - - - - - - - - - - - - - - - - - - - - - - -
      5'> GAAGTGGTGAGTTATATTGTTA >3'
                              ||
                          3'< ATTGTTATATTGAGTGGTGAAG <5'
   - - - - - - - - - - - - - - - - - - - - - - - - - - - - - - - - - - - - - - - -
        Min ΔG for C282 vs C282 is -0.13 kcal/mol.
    - - - - - - - - - - - - - - - - - - - - - - - - - - - - - - - - - - - - - - - -
      5'> CTAACAAAACTTATTCAAAAACATT >3'
                             ||  ||
                         3'< TTACAAAAACTTATTCAAAACAATC <5'
   - - - - - - - - - - - - - - - - - - - - - - - - - - - - - - - - - - - - - - - -
>>> - - - - - - - - - - - - - - - - - - - - - - - - - - - - - - - - - - - - - - - -  <<<
```

```
*****************************************************************************************
>>> - - - - - - - - - - - - - - - - - - - - - - - - - - - - - - - - - - - - - - - -  <<<
        Min ΔG for C283 vs C284 is -4.15 kcal/mol.
    - - - - - - - - - - - - - - - - - - - - - - - - - - - - - - - - - - - - - - - -
      5'> CCCTTAATTTTCAAAACTTATTCT >3'
           || |       | ||  | ||||
       3'< GGTAGAGTTTATATTTTAGAAGAA <5'
   - - - - - - - - - - - - - - - - - - - - - - - - - - - - - - - - - - - - - - - -
        Min ΔG for C283 vs C283 is 2.51 kcal/mol.
    - - - - - - - - - - - - - - - - - - - - - - - - - - - - - - - - - - - - - - - -
      5'> CCCTTAATTTTCAAAACTTATTCT >3'
                             |   |
                         3'< TCTTATTCAAAACTTTTAATTCCC <5'
   - - - - - - - - - - - - - - - - - - - - - - - - - - - - - - - - - - - - - - - -
        Min ΔG for C284 vs C284 is 2.71 kcal/mol.
    - - - - - - - - - - - - - - - - - - - - - - - - - - - - - - - - - - - - - - - -
      5'> AAGAAGATTTTATATTTGAGATGG >3'
                     || |   | ||
               3'< GGTAGAGTTTATATTTTAGAAGAA <5'
   - - - - - - - - - - - - - - - - - - - - - - - - - - - - - - - - - - - - - - - -
>>> - - - - - - - - - - - - - - - - - - - - - - - - - - - - - - - - - - - - - - - -  <<<
*****************************************************************************************
>>> - - - - - - - - - - - - - - - - - - - - - - - - - - - - - - - - - - - - - - - -  <<<
        Min ΔG for C285 vs C286 is -2.7 kcal/mol.
    - - - - - - - - - - - - - - - - - - - - - - - - - - - - - - - - - - - - - - - -
      5'> ATACTTCTTAAAAAAATAACCTTCT >3'
                 || ||||   ||
             3'< AAATTTTGGTTTTTGGATAAAAGAA <5'
   - - - - - - - - - - - - - - - - - - - - - - - - - - - - - - - - - - - - - - - -
        Min ΔG for C285 vs C285 is 2.69 kcal/mol.
    - - - - - - - - - - - - - - - - - - - - - - - - - - - - - - - - - - - - - - - -
      5'> ATACTTCTTAAAAAAATAACCTTCT >3'
                         | ||  || |
                     3'< TCTTCCAATAAAAAAATTCTTCATA <5'
   - - - - - - - - - - - - - - - - - - - - - - - - - - - - - - - - - - - - - - - -
        Min ΔG for C286 vs C286 is -4.78 kcal/mol.
    - - - - - - - - - - - - - - - - - - - - - - - - - - - - - - - - - - - - - - - -
      5'> AAGAAAATAGGTTTTTGGTTTTAAA >3'
                             ||||||
                         3'< AAATTTTGGTTTTTGGATAAAAGAA <5'
   - - - - - - - - - - - - - - - - - - - - - - - - - - - - - - - - - - - - - - - -
>>> - - - - - - - - - - - - - - - - - - - - - - - - - - - - - - - - - - - - - - - -  <<<
*****************************************************************************************
>>> - - - - - - - - - - - - - - - - - - - - - - - - - - - - - - - - - - - - - - - -  <<<
        Min ΔG for C287 vs C288 is -1.48 kcal/mol.
    - - - - - - - - - - - - - - - - - - - - - - - - - - - - - - - - - - - - - - - -
      5'> TTGTAAATGGTATGGAGATATATG >3'
                             || ||
                        3'< CTACACAATCTCTACTCATC <5'
   - - - - - - - - - - - - - - - - - - - - - - - - - - - - - - - - - - - - - - - -
        Min ΔG for C287 vs C287 is -1.9 kcal/mol.
    - - - - - - - - - - - - - - - - - - - - - - - - - - - - - - - - - - - - - - - -
      5'> TTGTAAATGGTATGGAGATATATG >3'
                           ||||||
                      3'< GTATATAGAGGTATGGTAAATGTT <5'
   - - - - - - - - - - - - - - - - - - - - - - - - - - - - - - - - - - - - - - - -
        Min ΔG for C288 vs C288 is 2.72 kcal/mol.
    - - - - - - - - - - - - - - - - - - - - - - - - - - - - - - - - - - - - - - - -
      5'> CTACTCATCTCTAACACATC >3'
                           ||
                      3'< CTACACAATCTCTACTCATC <5'
   - - - - - - - - - - - - - - - - - - - - - - - - - - - - - - - - - - - - - - - -
>>> - - - - - - - - - - - - - - - - - - - - - - - - - - - - - - - - - - - - - - - -  <<<
*****************************************************************************************
>>> - - - - - - - - - - - - - - - - - - - - - - - - - - - - - - - - - - - - - - - -  <<<
        Min ΔG for C289 vs C290 is -0.4 kcal/mol.
    - - - - - - - - - - - - - - - - - - - - - - - - - - - - - - - - - - - - - - - -
      5'> AGGGAGAGAAGAGTGGTAA >3'
            |     | |     |||
      3'< AACTAAATTACAATATATTAATTCCAT <5'
   - - - - - - - - - - - - - - - - - - - - - - - - - - - - - - - - - - - - - - - -
        Min ΔG for C289 vs C289 is 1.18 kcal/mol.
    - - - - - - - - - - - - - - - - - - - - - - - - - - - - - - - - - - - - - - - -
      5'> AGGGAGAGAAGAGTGGTAA >3'
                          ||
                     3'< AATGGTGAGAAGAGAGGGA <5'
   - - - - - - - - - - - - - - - - - - - - - - - - - - - - - - - - - - - - - - - -
        Min ΔG for C290 vs C290 is -0.0 kcal/mol.
    - - - - - - - - - - - - - - - - - - - - - - - - - - - - - - - - - - - - - - - -
      5'> TACCTTAATTATATAACATTAAATCAA >3'
              || |||  ||   ||  ||| ||
          3'< AACTAAATTACAATATATTAATTCCAT <5'
   - - - - - - - - - - - - - - - - - - - - - - - - - - - - - - - - - - - - - - - -
>>> - - - - - - - - - - - - - - - - - - - - - - - - - - - - - - - - - - - - - - - -  <<<
```

```
****************************************************************************************
>>> - - - - - - - - - - - - - - - - - - - - - - - - - - - - - - - - - - - - - - - -  <<<
        Min ΔG for C291 vs C292 is -1.26 kcal/mol.
    - - - - - - - - - - - - - - - - - - - - - - - - - - - - - - - - - - - - - - - -
      5'> AAAAAAAAAACTCCTACTTTTCCA >3'
                       || | ||  ||
                   3'< GAAGTAATTGTTGATAGATTTTTTG <5'
   - - - - - - - - - - - - - - - - - - - - - - - - - - - - - - - - - - - - - - - -
        Min ΔG for C291 vs C291 is 1.53 kcal/mol.
    - - - - - - - - - - - - - - - - - - - - - - - - - - - - - - - - - - - - - - - -
      5'> AAAAAAAAAACTCCTACTTTTCCA >3'
             ||||   |    |    ||||
      3'< ACCTTTTCATCCTCAAAAAAAAAA <5'
   - - - - - - - - - - - - - - - - - - - - - - - - - - - - - - - - - - - - - - - -
        Min ΔG for C292 vs C292 is 0.64 kcal/mol.
    - - - - - - - - - - - - - - - - - - - - - - - - - - - - - - - - - - - - - - - -
      5'> GTTTTTTAGATAGTTGTTAATGAAG >3'
           ||  |||    | |  |||   ||
      3'< GAAGTAATTGTTGATAGATTTTTTG <5'
   - - - - - - - - - - - - - - - - - - - - - - - - - - - - - - - - - - - - - - - -
>>> - - - - - - - - - - - - - - - - - - - - - - - - - - - - - - - - - - - - - - - -  <<<
****************************************************************************************
>>> - - - - - - - - - - - - - - - - - - - - - - - - - - - - - - - - - - - - - - - -  <<<
        Min ΔG for C293 vs C294 is -2.06 kcal/mol.
    - - - - - - - - - - - - - - - - - - - - - - - - - - - - - - - - - - - - - - - -
      5'> AAAATAGTAGAGAGAGGTGTAA >3'
                           || ||
                      3'< AACCTTTCTAAACTTCTTATAAAC <5'
   - - - - - - - - - - - - - - - - - - - - - - - - - - - - - - - - - - - - - - - -
        Min ΔG for C293 vs C293 is 1.18 kcal/mol.
    - - - - - - - - - - - - - - - - - - - - - - - - - - - - - - - - - - - - - - - -
      5'> AAAATAGTAGAGAGAGGTGTAA >3'
                             ||
                        3'< AATGTGGAGAGAGATGATAAAA <5'
   - - - - - - - - - - - - - - - - - - - - - - - - - - - - - - - - - - - - - - - -
        Min ΔG for C294 vs C294 is 0.84 kcal/mol.
    - - - - - - - - - - - - - - - - - - - - - - - - - - - - - - - - - - - - - - - -
      5'> CAAATATTCTTCAAATCTTTCCAA >3'
                            ||  ||
                        3'< AACCTTTCTAAACTTCTTATAAAC <5'
   - - - - - - - - - - - - - - - - - - - - - - - - - - - - - - - - - - - - - - - -
>>> - - - - - - - - - - - - - - - - - - - - - - - - - - - - - - - - - - - - - - - -  <<<
****************************************************************************************
>>> - - - - - - - - - - - - - - - - - - - - - - - - - - - - - - - - - - - - - - - -  <<<
        Min ΔG for C295 vs C296 is -5.21 kcal/mol.
    - - - - - - - - - - - - - - - - - - - - - - - - - - - - - - - - - - - - - - - -
      5'> GGTGGATTATGTTGAGGTAG >3'
                      |||| ||
                 3'< AACTACAAAAACAAAACAAAACCA <5'
   - - - - - - - - - - - - - - - - - - - - - - - - - - - - - - - - - - - - - - - -
        Min ΔG for C295 vs C295 is 0.64 kcal/mol.
    - - - - - - - - - - - - - - - - - - - - - - - - - - - - - - - - - - - - - - - -
      5'> GGTGGATTATGTTGAGGTAG >3'
                           ||
                      3'< GATGGAGTTGTATTAGGTGG <5'
   - - - - - - - - - - - - - - - - - - - - - - - - - - - - - - - - - - - - - - - -
        Min ΔG for C296 vs C296 is 3.04 kcal/mol.
    - - - - - - - - - - - - - - - - - - - - - - - - - - - - - - - - - - - - - - - -
      5'> ACCAAAACAAAACAAAAACATCAA >3'
                              |  |
                          3'< AACTACAAAAACAAAACAAAACCA <5'
   - - - - - - - - - - - - - - - - - - - - - - - - - - - - - - - - - - - - - - - -
>>> - - - - - - - - - - - - - - - - - - - - - - - - - - - - - - - - - - - - - - - -  <<<
****************************************************************************************
>>> - - - - - - - - - - - - - - - - - - - - - - - - - - - - - - - - - - - - - - - -  <<<
        Min ΔG for C297 vs C298 is -7.67 kcal/mol.
    - - - - - - - - - - - - - - - - - - - - - - - - - - - - - - - - - - - - - - - -
      5'> AATTAAAATTCATTTAAAACCATTTCAT >3'
                               |||||||
                        3'< GAATAAAGTATTGGATTGAAATTATA <5'
   - - - - - - - - - - - - - - - - - - - - - - - - - - - - - - - - - - - - - - - -
        Min ΔG for C297 vs C297 is -1.3 kcal/mol.
    - - - - - - - - - - - - - - - - - - - - - - - - - - - - - - - - - - - - - - - -
      5'> AATTAAAATTCATTTAAAACCATTTCAT >3'
                                    ||
                                3'< TACTTTACCAAAATTTACTTAAAATTAA <5'
   - - - - - - - - - - - - - - - - - - - - - - - - - - - - - - - - - - - - - - - -
        Min ΔG for C298 vs C298 is 0.49 kcal/mol.
    - - - - - - - - - - - - - - - - - - - - - - - - - - - - - - - - - - - - - - - -
      5'> ATATTAAAGTTAGGTTATGAAATAAG >3'
                        ||||   ||||
                   3'< GAATAAAGTATTGGATTGAAATTATA <5'
   - - - - - - - - - - - - - - - - - - - - - - - - - - - - - - - - - - - - - - - -
>>> - - - - - - - - - - - - - - - - - - - - - - - - - - - - - - - - - - - - - - - -  <<<
```

```
********************************************************************************
>>> - - - - - - - - - - - - - - - - - - - - - - - - - - - - - - - - - - -  <<<
        Min ΔG for C299 vs C300 is -3.03 kcal/mol.
    - - - - - - - - - - - - - - - - - - - - - - - - - - - - - - - - - - -
      5'> TAACATTTCAAACTTATTCTACCAA >3'
                  | | ||| |  ||| ||
             3'< GGGTGGAAAATTATGATTATTTATA <5'
   - - - - - - - - - - - - - - - - - - - - - - - - - - - - - - - - - - - -
        Min ΔG for C299 vs C299 is 2.56 kcal/mol.
    - - - - - - - - - - - - - - - - - - - - - - - - - - - - - - - - - - -
      5'> TAACATTTCAAACTTATTCTACCAA >3'
               ||   |   ||   |   ||
           3'< AACCATCTTATTCAAACTTTACAAT <5'
   - - - - - - - - - - - - - - - - - - - - - - - - - - - - - - - - - - - -
        Min ΔG for C300 vs C300 is 4.62 kcal/mol.
    - - - - - - - - - - - - - - - - - - - - - - - - - - - - - - - - - - -
      5'> ATATTTATTAGTATTAAAAGGTGGG >3'
                            |  |
                     3'< GGGTGGAAAATTATGATTATTTATA <5'
   - - - - - - - - - - - - - - - - - - - - - - - - - - - - - - - - - - - -
>>> - - - - - - - - - - - - - - - - - - - - - - - - - - - - - - - - - - -  <<<
********************************************************************************
>>> - - - - - - - - - - - - - - - - - - - - - - - - - - - - - - - - - - -  <<<
        Min ΔG for C301 vs C302 is -2.04 kcal/mol.
    - - - - - - - - - - - - - - - - - - - - - - - - - - - - - - - - - - -
      5'> ATTCAAAATTACTCATTTAAATTTCC >3'
            ||| |   |   |    ||| | |
       3'< TAGTATATTTTGTTTTTGTTATATGTTT <5'
   - - - - - - - - - - - - - - - - - - - - - - - - - - - - - - - - - - - -
        Min ΔG for C301 vs C301 is -1.61 kcal/mol.
    - - - - - - - - - - - - - - - - - - - - - - - - - - - - - - - - - - -
      5'> ATTCAAAATTACTCATTTAAATTTCC >3'
                            ||||||
                      3'< CCTTTAAATTTACTCATTAAAACTTA <5'
   - - - - - - - - - - - - - - - - - - - - - - - - - - - - - - - - - - - -
        Min ΔG for C302 vs C302 is -1.59 kcal/mol.
    - - - - - - - - - - - - - - - - - - - - - - - - - - - - - - - - - - -
      5'> TTTGTATATTGTTTTTGTTTTATATGAT >3'
                                 || ||
                             3'< TAGTATATTTTGTTTTTGTTATATGTTT <5'
   - - - - - - - - - - - - - - - - - - - - - - - - - - - - - - - - - - - -
>>> - - - - - - - - - - - - - - - - - - - - - - - - - - - - - - - - - - -  <<<
********************************************************************************
>>> - - - - - - - - - - - - - - - - - - - - - - - - - - - - - - - - - - -  <<<
        Min ΔG for C303 vs C304 is 0.38 kcal/mol.
    - - - - - - - - - - - - - - - - - - - - - - - - - - - - - - - - - - -
      5'> AAATAAATTAATTTTTAATCTTTATTCATC >3'
                          ||    || | |
                      3'< TTGTGTGTATGAATAAAATTATAAAATT <5'
   - - - - - - - - - - - - - - - - - - - - - - - - - - - - - - - - - - - -
        Min ΔG for C303 vs C303 is 1.77 kcal/mol.
    - - - - - - - - - - - - - - - - - - - - - - - - - - - - - - - - - - -
      5'> AAATAAATTAATTTTTAATCTTTATTCATC >3'
                || |||    | |    ||| ||
           3'< CTACTTATTTCTAATTTTTAATTAAATAAA <5'
   - - - - - - - - - - - - - - - - - - - - - - - - - - - - - - - - - - - -
        Min ΔG for C304 vs C304 is -0.28 kcal/mol.
    - - - - - - - - - - - - - - - - - - - - - - - - - - - - - - - - - - -
      5'> TTAAAATATTAAAATAAGTATGTGTGTT >3'
            || | |   |  ||  |   | | ||
        3'< TTGTGTGTATGAATAAAATTATAAAATT <5'
   - - - - - - - - - - - - - - - - - - - - - - - - - - - - - - - - - - - -
>>> - - - - - - - - - - - - - - - - - - - - - - - - - - - - - - - - - - -  <<<
********************************************************************************
################################################################################
#                                                                              #
#                                 End of Report                                #
#                                                                              #
################################################################################
```
